# Pan-genome and reverse vaccinology for a multi-epitope vaccine against circulating post-2022 Monkeypox virus strains

**DOI:** 10.1371/journal.pone.0351881

**Published:** 2026-06-22

**Authors:** Md Al Saber, Md. Hasan Jafre Shovon, Md. Imtiaz, Sadia Jannat Tauhida, Sowmitro Das, Md Mohaimenul Islam Tareq, Miss Ismoth Ara Tripty, Md Ridoy Hossain, Labib Shahriar Siam, Md Nazmul Hasan Zilani, Saifullah, S.M. Khaledur Rahman, Umama Khan, Md Nazmul Hasan

**Affiliations:** 1 Laboratory of Pharmaceutical Biotechnology and Bioinformatics, Department of Genetic Engineering and Biotechnology, Jashore University of Science and Technology, Jashore, Bangladesh; 2 Department of Pharmacy, Faculty of Biological Science and Technology, Jashore University of Science and Technology, Jashore, Bangladesh; Albert Einstein College of Medicine, UNITED STATES OF AMERICA

## Abstract

**Background:**

Since the 2022 outbreak, the number of Monkeypox cases worldwide has been increasing at an alarming rate. As of August 2024, approximately 99,000 people have been infected with the virus. The severity of this situation is further highlighted by the World Health Organization's (WHO) classification of the Mpox virus as a Public Health Emergency of International Concern (PHEIC) due to the increased fatality rate of approximately 3.6% in clade I.

**Objectives:**

Targeting the virus's membrane-bound, enveloped, and extracellular proteins, our goal was to computationally develop and assess a broad-spectrum multi-epitope vaccine that elicits humoral and adaptive immune responses against Monkeypox virus (MPXV) infection.

**Methods:**

During an outbreak, a pan-genome-based reverse vaccinology approach can offer rapid, practical solutions to enduring problems in experimental vaccine design. The method involved screening 16 monkeypox genomes to identify viral targets, from which viral proteins were selected based on their antigenicity, location, and solubility. Immunoinformatics methods and algorithms were used to extract the proteins’ putative T-cell and B-cell epitopes, which were combined to form several vaccine constructions. The tertiary structure of the chimeric vaccine construct's interaction with Toll-like receptor 4 (TLR4) was thoroughly assessed using the advanced techniques of molecular docking and molecular dynamics simulation.

**Results:**

A pan-genomic analysis identified 80 core genes, which were then screened for proteins suitable for epitope-based vaccine design. From four of these selected proteins, T-cell and B-cell epitopes were extracted to create four distinct vaccine constructs. Appropriate adjuvants and linkers were incorporated into each construct to enhance its potential efficacy. Stability and immunogenicity analyses of each vaccine design yielded promising results. These findings suggest that the vaccine constructs could be effective in preventing monkeypox, warranting further experimental validation and supporting the application of similar strategies to combat other viral illnesses.

## Introduction

Since the 2022 outbreak, the number of Monkeypox (MPXV) cases has steadily increased worldwide, reaching approximately 99,000 infections by August 2024. In response, the World Health Organization (WHO) designated the outbreak a Public Health Emergency of International Concern (PHEIC) [1]. This declaration was particularly driven by concerns over Clade I of the virus, which is associated with a higher case fatality rate of around 3.6% [2]. Although historically endemic to the Democratic Republic of Congo and Central Africa, the Monkeypox virus (MPXV) has spread globally, largely driven by human migration [3]. While Asian nations have generally reported low case numbers, sporadic occurrences have been documented in countries such as India, China, and Thailand, with India reporting 30 cases since the outbreak began [2]. MPXV is a double-stranded DNA (dsDNA) virus belonging to the *Orthopoxvirus* genus and the *Poxviridae* family. Its structure is characterized by an oval-shaped capsid that lacks clear helical or icosahedral symmetry and is enclosed by an outer lipid envelope [4].;spread Historical data indicate that the smallpox vaccine provided approximately 85% cross-protection against monkeypox. Therefore, the eradication of smallpox and the subsequent cessation of routine orthopoxvirus vaccination for over 40 years are thought to be contributing factors to the re-emergence of MPXV [4,5]. The genetic evolution of the virus is another significant factor in the re-emergence of monkeypox. For instance, four distinct viral lineages were identified from samples in the Democratic Republic of the Congo. Moreover, recent research on the evolution and epidemiology of MPXV in Nigeria and worldwide has cited gene loss/gain and recombination events as key drivers in its development [5].There is currently no vaccination specifically for MPXV, despite reports of cross-protection through vaccinia-viral vaccines (ACAM2000 and JYNNEOSTM) [3]. When prescribed for severe MPXV infections, high-dose antiviral treatments (cidofovir, tecovirimat, and brincidofovir) sometimes cause hepatic dysfunctions, whereas low-dose therapies increase the chance of recurrence [3–5]. The primary limitation of the current MPXV vaccines is their ability to induce a persistent immune response and their specificity, particularly considering the pathogen's changing nature [5]. The present study utilized a reverse vaccinology and pan-genomic approach to develop a multi-epitope vaccine designed to stimulate human immunity against MPXV. This vaccine was constructed by generating multiple epitopes from selected MPXV proteins to elicit both humoral and cellular immune responses while ensuring a high safety profile.

## 2. Method and materials

### 2.1 Sequence Retrieval and Pre-screening Phase

In our study, we systematically sourced and subsequently downloaded a total of 16 distinct whole genomic sequences, along with their corresponding proteome sequences, which collectively represent an impressive sample size of n = 16, all of which pertain to the MPXV and originate from 16 diverse geographical regions, sourced directly from the National Center for Biotechnology Information (NCBI) database, specifically to conduct a comprehensive pan-genomic analysis. To facilitate this intricate analysis, we first reannotated the retrieved genomes with Prokka v1.14.6, and then used two specialized bioinformatics software programs, namely ROARY (v3.13.0) and BPGA (version 1.3), for the pangenome analysis [6]. Roary was provided with input from the annotated genomes generated by Prokka. Roary created a multi-FASTA alignment of core genes using PRANK v.170427 to create the gene presence/absence matrix [7]. Furthermore, in the BPGA analysis, we established a sequence identity criterion for the USEARCH algorithms, judiciously set at 90%. This decision was made to effectively cluster the genomic sequences in a manner that maximizes the reliability of our results [7]. Moreover, in addition to the aforementioned analyses, BPGA facilitated the generation and accessibility of core, accessory, and distinct proteomic datasets, all of which are intricately linked to the genomic data, thereby providing a holistic view of the genetic and proteomic landscape of MPXV.

### 2.2 Subtractive Proteomics Filters

The fundamental core protein sequences of MPXV, obtained through comprehensive pan-genomic analysis, serve as the primary focus for the development of multi-epitope subunit vaccine models. Initially, we excluded all MPXV core proteins that exhibit homology to human proteins, retaining only those that lack substantial similarity to human counterparts. The exclusion of MPXV proteins that closely resemble human proteins was implemented to mitigate the risk ofautoimmunity or tolerance to foreign antigens. We employed the Non-redundant protein sequences database via blastP from NCBI to identify non-homologous proteins [8]. Subsequently, the non-homologous core proteins underwent evaluation for their subcellular localization. Proteins located in the cell membrane or in the extracellular region are prioritized as essential targets for potential vaccine candidates [9]. These membrane or extracellular proteins are accessible on the exterior of the viral cell and play a pivotal role in facilitating entry into the host cell through specific interactions. Herein, we employed the deepTMHMM server as a computational resource for the meticulous examination of the subcellular localization of various proteins [10]. Furthermore, we conducted a thorough analysis of the transmembrane helices of those proteins that were systematically filtered using the TMHMM 2.0 server, thereby ensuring the integrity of our findings [11]. Transmembrane helix proteins are predominantly situated within the lipid bilayer of cellular membranes and play a multitude of critical roles, encompassing essential processes such as cell signaling, transport mechanisms, and overall cellular communication [12]. It is noteworthy that transmembrane helices, which are significantly shielded by the surrounding membrane, exhibit reduced accessibility to antibodies, thereby resulting in a diminished immunogenic response when exposed to the immune system. Subsequently, we evaluated the antigenicity and allergenicity of the aforementioned filtered proteins using the VaxiJen v2.0 and AllerTOP v.2.0 servers, during which proteins were systematically excluded from further consideration if classified as non-antigenic or allergenic [13,14]. Lastly, we assessed the physicochemical properties of these selected proteins using the ProtParam tool on the ExPASy server, focusing on critical parameters such as molecular weight, instability index, aliphatic index, and grand average hydrophobicity, thereby providing a comprehensive characterization of these biomolecules [15].

### 2.3 Immune cell epitope prediction

#### 2.3.1 B Cell Epitope Prediction.

One particular antigenic region that interacts strongly with B cells is the B cell epitope [16]. B cells might then go on to develop memory cells and antibodies that are specific to that antigen [17]. Hydrophilicity, accessibility, and the β turn region are the three factors that split the B cell epitope [18]. The Immune Epitope Database (IEDB) website (http://tools.iedb.org/main/bcell/) provided the following approaches, which we used to predict B cell epitopes: BepiPred with a baseline threshold of 0.5. Finally, the Vaxijen v.2.0, AllerTOP v.2, and toxinpred servers exclude low-antigenic, allergenic, and toxic peptide sequences [19,20].

#### 2.3.2 MHC Class I and II Epitope Prediction.

The Immune Epitope Database (IEDB) web server (http://tools.iedb.org/main/bcell/) was used to predict MHC class I epitopes. Peptides in the top 1% with a percentile rank less than 0.5 were chosen [21]. The organism was set to human, and the specified alleles were HLA-A01:01, HLA-A02:01, HLA-A03:01, HLA-A11:01, and HLA-B35:01. The IEDB web server was also used to predict MHC class II epitopes, with a percentile rank of <0.5 selected. DRB101:01, DRB107:01, DRB103:01, DRB104:01, DRB108:01, DRB111:01, DRB115:01, and DRB1*13:01 were the DRB alleles that were defined. Lastly, the Vaxijen v2.0, AllerTOP v.2, and ToxinPred servers were used to assess the antigenicity, allergenicity, and toxicity of the peptide sequences, thereby excluding peptides with low antigenicity, allergenicity, and toxic characteristics [22].

### 2.4 Multi-epitope Vaccine Construction

The Cytotoxin and helper T cell epitopes are connected to the unique GPGPG glycine-proline linker sequence, whereas the B cell epitopes are related to the Kk linker sequence [23]. The Large Ribosomal Subunit Protein bL12 has made contact with the N-termini of the epitopes, and the EAAAK stiff linker has connected this adjuvant to the epitopes [24]. The physicochemical properties of the vaccine, including molecular weight (MW), theoretical pI, atomic and amino acid composition, extinction coefficient, estimated half-life, instability index, aliphatic index, and grand average of hydropathicity (GRAVY), were computationally assessed using the Protparam tool on the ExPASy server [25]. Furthermore, the developed multi-epitope vaccination was subjected to additional assessments of its antigenicity, allergenicity, and toxicity using the servers VaxiJen, AllerTOP v.2.0, and ToxinPred, respectively [26].

### 2.5 Secondary Structure and Tertiary Structure Prediction

The sophisticated tools PSIPRED and Alphafold 2 were expertly employed in our study to design and elucidate the vaccine's secondary and tertiary structural configurations, respectively [27,28].The PSIPRED server is an advanced protein structure prediction tool that uses the PSI–BLAST algorithm to analyze sequences. From high-quality primary sequence data, we generated a comprehensive two-dimensional model that accurately predicts a protein's secondary structure with remarkable precision. Using AlphaFold2, an advanced AI tool for protein structure prediction, we constructed a three-dimensional model of the protein, which enhanced our capacity for deep learning and pattern recognition. The model's local confidence was assessed using the predicted local distance difference test (pLDDT), a per-residue score ranging from 0 to 100, with higher values indicating greater confidence. A pLDDT above 90 indicates high accuracy in both backbone and side chains. At the same time, scores above 70 suggest reliable backbone prediction but potential side-chain misplacement, providing insight into the reliability of different protein regions.

### 2.6 Structure Refinement and Validation

We further engaged the GalaxyRefine server to refine the three-dimensional structure we had previously established with AlphaFold 2, thereby improving the quality and accuracy of our structural models.Galaxy Refine is a powerful web server that employs advanced techniques for protein refinement and structure prediction. By precisely refining key geometric features of the model, we optimized its structural configuration, ensuring proper folding and enhanced stability [29]. To assess the quality of the vaccine model, we employed the ProSA-web server, which is adept at analyzing and displaying scoring metrics for high-quality protein models [30]. This server also calculates the protein structure's energy and helps identify potential flaws or deficiencies in the model, thereby providing critical insights into its overall quality and reliability.In addition, we checked the Ramachandran Plot to validate the accuracy of the protein structure using the SAVES v.6.0 web application, which allowed us to rigorously examine whether our residues were located within the favored regions of the plot, thereby confirming the structural integrity of our model [31]. By analyzing the allowed and disallowed regions corresponding to phi and psi angles, this method significantly enhances our understanding of protein structure. It is fundamentally essential for both protein modeling and vaccine design, ensuring that the resulting models align with established principles of structural biology.

### 2.7 Disulfide Engineering and Codon Optimization

To enhance both stability and expression efficacy,we employed methodologies including disulfide engineering and codon optimization. For the disulfide engineering, we utilized the Disulfide by Design 2 (DbD2) computational server [32]. This tool systematically facilitates the introduction of disulfide bonds, thereby enhancing vaccine stability without compromising the integrity of the overall antigenic determinants. More specifically, it identifies cysteine residues within the vaccine and strategically introduces disulfide bonds as needed to enhance the vaccine's overall structural integrity. Codon optimization was performed using the VectorBuilder server, specifically to adapt the vaccine constructs for the E. coli K-12 strain [33]. We assessed the Codon Adaptation Index (CAI) value alongside the overall GC content of the engineered vaccine. Codon optimization is imperative for ensuring the high-level expression of vaccine proteins, achieved by aligning the DNA sequence with the codon usage preferences characteristic of the E. coli expression system. The VectorBuilder played a pivotal role by optimizing the vaccine DNA sequences to maximize translation efficiency. Subsequently, the modified sequences were synthesized and integrated into expression vectors specifically designed for E. coli K-12, a strain renowned for its robust protein expression capabilities.

### 2.8 Molecular Docking of the Protein with TLR-4

TLR4 plays a role in the innate immune response, particularly in activating pro-inflammatory cytokines during viral infections, and has been shown to recognize pathogen-associated molecular patterns (PAMPs). TLR4 is an excellent target for the development of vaccine candidates since previous studies have demonstrated its crucial role in regulating immune responses to a variety of viral diseases. This receptor was selected because of its vital function in initiating immunological signaling pathways that lead to protective immunity, even though other receptors may also be involved in viral immune responses [34]. To determine TLR-4 docking with the vaccine protein, the Cluspro server was utilized. The Protein Data Bank supplied the TLR-4 structural coordinates. To visualize the docked structures and assess the interaction between TLR-4 and the vaccine protein, UCSF Chimera and Ligplot software were utilized [35].

### 2.9 Molecular Dynamics Simulation

The Schrödinger suite's Desmond v3.6 was used to do molecular dynamics (MD) simulations. The OPLS3e force field was used to describe atomic interactions and replicate how the vaccine models would behave in a biological setting. To create a hydrated environment comparable to physiological conditions, each vaccine model was solvated in an orthorhombic periodic boundary box filled with TIP3P water molecules. The system was neutralized by adding the appropriate counterions (Na+ or Cl-) to balance the total charge. Using the steepest descent approach, which minimizes potential energy by removing high-energy configurations and steric clashes, the system was initializedfrom a stable configuration [36].

To enable the system to adapt to the simulated conditions, two equilibration phases were performed after minimization. Initially, the Nose-Hoover thermostat was used to apply an NVT ensemble (constant Number, Volume, and Temperature) for 50 PS to maintain 300 K During this stage, the system's volume remained constant while the temperature stabilized. The Martyna-Tobias-Klein barostat was then used to equilibrate the system for 50 PS utilizing NPT (constant Number, Pressure, and Temperature) [37]. This allowed the system's volume to fluctuate as needed to achieve equilibrium density while maintaining a pressure of 1 bar. A 50 ns manufacturing run with a 2-femtosecond (fs) time step was performed after equilibration to record atomic movements and interactions within the system. Atomic coordinates were captured at 0.05 ns intervals during this phase, providing a high temporal resolution for the dynamics of the vaccine models.Atomic coordinates were captured at 0.05 ns intervals during this phase, providing a high temporal resolution for the dynamics of the vaccine models [38]. The divergence of the protein's backbone and overall structure from its initial configuration was tracked using the Root Mean Square divergence (RMSD), which provides a measure of global stability. The flexibility of individual residues over time was evaluated using Root Mean Square Fluctuation (RMSF), which highlighted vaccination areas most susceptible to structural alterations or fluctuations [39].

Time-dependent changes in structure and interactions were visualized by post-simulation analysis using the Simulation Interaction Diagram (SID). Plotting the RMSD and RMSF data enabled a better understanding of the vaccination models’ dynamic behavior under physiological conditions, revealing their structural stability. This thorough MD simulation method ensured that the vaccine candidates maintained structural integrity in a physiologically relevant environment, providing vital information on their stability, flexibility, and overall behavior [40,41]. Afterward, the COCOMAPS 2.0 tool was used to conduct a thorough study and visualization of the contacts at the vaccine-TLR4 complex interface [42,43].

### 2.10 Immune Simulation Analysis

The ability to swiftly predict and evaluate immune responses to vaccine candidates makes in silico immune simulations essential for advancing vaccines. These simulations accelerate the development process and streamline the identification of viable vaccines for further scrutiny. This is accomplished by identifying optimal antigen components, dosages, and formulations [44]. The formulated vaccine was uploaded to the C-IMMSIM server in FASTA format to evaluate the anticipated immune response post-vaccination [45]. The analysis outcomes were then retrieved for detailed review. Three simulated injections were administered at 1, 84, and 168 hours, with each time step corresponding to 8 hours in reality.The default settings of the remaining server parameters were preserved.

## 3. Result

### 3.1 Pre-Screening Phase

**Fig 1:**
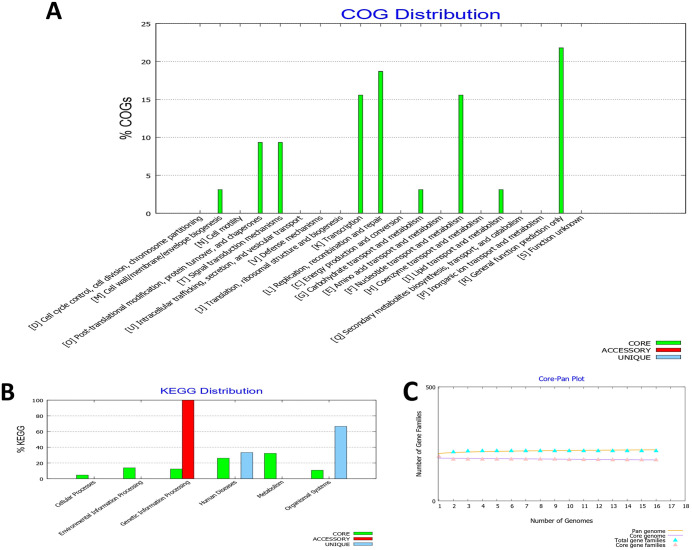
Visualization of the distributed proteomic data profiling of Monkeypox virus. Herein, (**A**) shows COG distribution, (**B**) shows KEGG distribution, and (**C**) shows the Core-pan plot of pan-genomic analysis.

**Fig 2:**
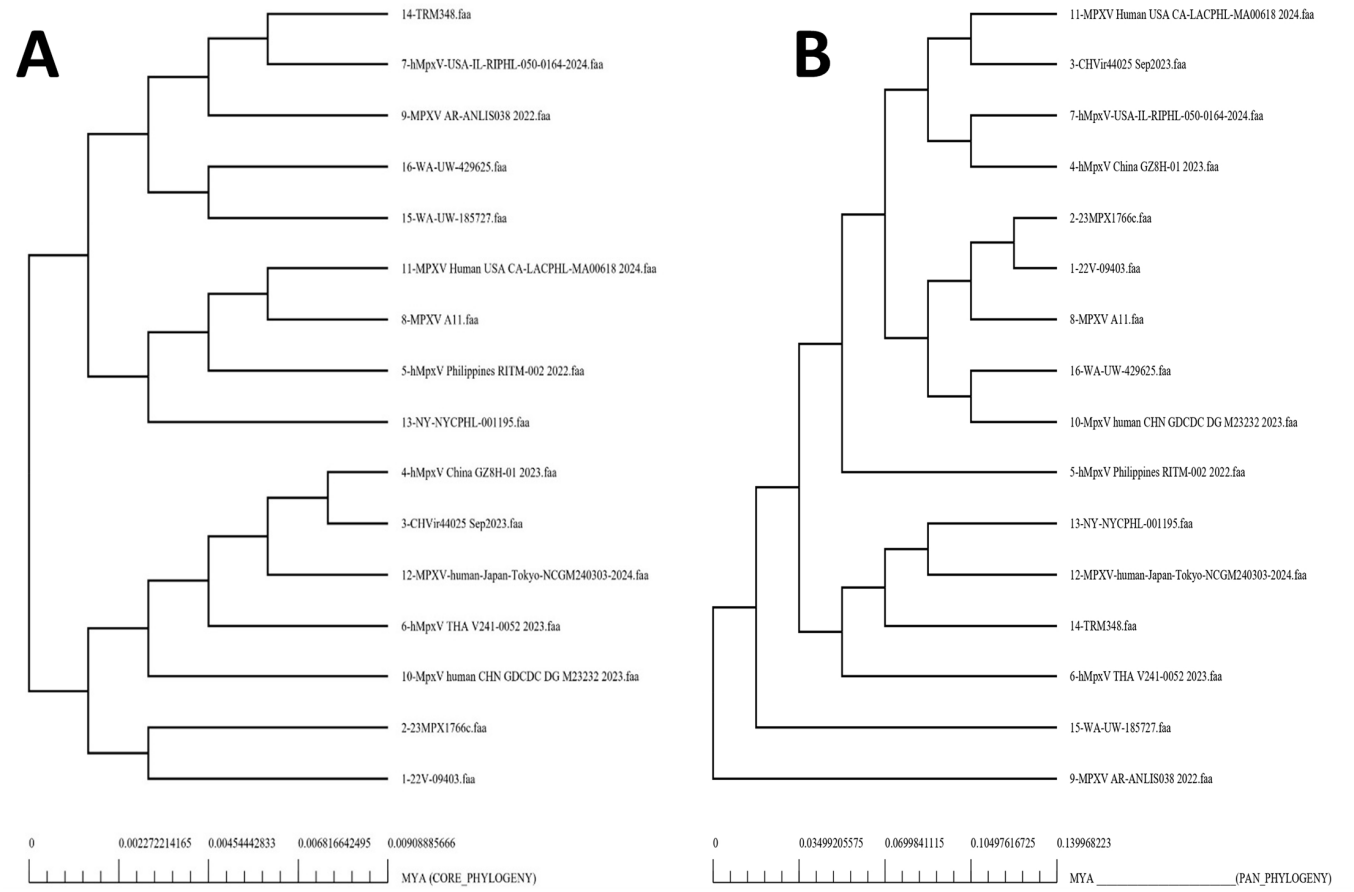
Visualization of (A) Core Proteins phylogenetic tree and (B) Phylogenetic tree of all the expressed proteins of Monkeypox virus.

To conduct a comprehensive pan-genome analysis that would facilitate a meticulous evaluation of the genetic diversity across all 16 strains of the retrieved MPXV, we employed both Roary and BPGA, thereby enabling a robust cross-comparison of the core genes identified within these genomes.Before implementing Roary for analysis, we meticulously annotated the genomes using Prokka, a widely recognized tool for genomic annotation that enhances understanding of gene functions and structures. Upon executing Roary, our analysis identified 170 core genes conserved across the assessed strains (S1 File).In comparison, a subsequent analysis using BPGA indicated a slightly higher count of 180 protein-encoding core genes, highlighting a noteworthy discrepancy between the two methodologies (S2 File).Beyond the mere provision of essential statistical data regarding the counts of core, accessory, and unique genes, BPGA further enriched our analysis by generating a plethora of informative distributions, which included, but were not limited to, the Clusters of Orthologous Groups (COG) analysis, histogram representations of gene distributions, as well as the Kyoto Encyclopedia of Genes and Genomes (KEGG) distribution analyses, alongside default visualizations of core pan plots, as depicted in Fig 1A, 1B, and 1C. Additionally, BPGA contributed to our understanding of phylogenetic relationships by generating both core and pan phylogenetic trees, which are visually represented in Fig 2A and 2B, thereby offering insights into the evolutionary dynamics of the MPXV strains. Given the significance of the core proteins identified, we strategically targeted them for further analytical efforts to develop a multi-epitope subunit vaccine specifically designed to combat MPXV, thereby addressing a critical gap in current preventive strategies against this viral pathogen.

### 3.2 Screening and Selection of Potential Vaccine Candidates

Among the 180 core proteins identified in our study, a total of 135 proteins were determined to be non-homologous to those found in the human genome, and critically, of these non-homologous proteins, a significant subset of 30 proteins was identified as residing within the membrane of the viral cell, indicating their potential role in viral pathology (S3 File). Upon conducting a thorough examination of the antigenicity of the remaining proteins, we isolated 16 proteins that exhibited antigenic properties, suggesting their relevance to immune response mechanisms (S4 File). In addition, we analyzed the transmembrane helices of the remaining proteins, systematically discarding those that scored above a threshold of 1, ultimately determining that all 16 proteins retained scores of 0 or 1, thereby qualifying them for further investigation (S4 File). Moreover, we undertook a comprehensive assessment of the physicochemical properties of these proteins, excluding those with a molecular weight exceeding 110 kDa and those deemed unstable or hydrophobic. Our analyses also included an evaluation of the aliphatic index, leading us to retain only those proteins that showed elevated scores in this metric, which serves as an indicator of protein stability and functionality. Following our thorough physicochemical properties analysis, we successfully identified six proteins as ideal candidates for targeting in the strategic design of a multi-epitope vaccine to combat the pathogenic effects of MPXV, thereby advancing vaccine development efforts against this viral threat (S4 File).

### 3.3 Epitope Prediction and Prioritization Phase

For epitope prediction, we used IEDB tools to assess the six initially selected proteins, evaluating their potential for B-cell epitopes and binding to MHC I and MHC II.Only four of six proteins demonstrated sufficient immunogenicity and binding affinity during this phase, and these were taken for further analysis. All the six proteins are namely CORE_REP Org2 Gene185# (**Soluble interferon-gamma receptor-like protein)**, CORE_REP Org4_Gene19# (**hypothetical protein)**, CORE_REP Org11_Gene13# (**Interleukin-18-binding protein)**, CORE_REP Org6 Gene199# (**Virulence protein)**, CORE_REP Org10 Gene113# (**Late transcription factor VLTF-2)**, and CORE_REP Org2 Gene150# (**Envelope protein A28 homolog)**. Among them, Org11_Gene13# and CORE_REP Org4_Gene19# were discarded due to fewer immunogenic epitopes, and the remainingfour proteins were utilized for further progress. In **CORE_REP Org2_Gene185#,** BepiPred predicted 11 B-cell epitopes at a threshold of 0.5, leading to the selection of 5 epitopes with high antigenicity scores. MHC I analysis identified peptides ranking in the top 1% for HLA-A01:01, HLA-A02:01, HLA-A03:01, HLA-A11:01, and HLA-B35:01, yielding 20 prioritized candidates. MHC II predictions revealed 11 epitopes demonstrating strong binding affinity to DRB1 alleles, particularly DRB101:01 and DRB107:01 **(CORE_REP Org2_Gene185#)** (supplementary 5). In CORE_REP Org6_Gene199#, the analysis yielded seven predicted B cell epitopes, of which four were selected based on antigenicity.MHC I analysis highlighted 13 epitopes exhibiting robust binding profiles, while MHC II predictions pointed to 13 epitopes with significant binding potential to DRB1 alleles **(CORE_REPOrg6_Gene199#)** (S5 File). Regarding CORE_REP Org10_Gene113**#**, 4 B cell epitopes were prioritized, while MHC I predictions identified 100 potential epitopes with high affinity to critical HLA alleles. MHC II predictions underscored 27 epitopes targeting DRB1 for optimal helper T cell activation **(CORE_REPOrg10_Gene113#)** (S5 File). Lastly, in Org2_Gene150#, analysis prioritized four high-scoring B-cell epitopes and identified 6 MHC I epitopes with strong HLA-binding affinity. MHC II analysis selected 13 epitopes with high binding affinity to DRB1 alleles (Org2_Gene150#) (S5 File). This comprehensive approach ensured the selection of epitopes with optimal immunogenic and binding properties derived from each protein.

### 3.4 Multi-epitope Vaccine Construction

Antigenicity, allergenicity, and toxicity evaluations were conducted on prioritized B cell, MHC I, and MHC II epitopes using Vaxijen v2.0, AllerTop v.2.0, and ToxinPred servers. Non-allergenic, non-toxic epitopes with high antigenic potential were selected for vaccine design. Vaccine model 1 included epitopes from CORE_REP Org2_Gene185# and consisted of two B cells and three MHC I and MHC II epitopes (Table 1). T cell epitopes were linked using a GPGPG linker to enhance immune response, while B cell epitopes were connected using a KK linker. The large ribosomal subunit protein bL12 served as an adjuvant, attached to the epitope construct via an EAAAK stiff linker. This configuration resulted in a construct of 255 amino acids. In Vaccine Model 2, epitopes collected from CORE_REP Org6_Gene199# followed similar antigenic selection criteria and linker configurations, which brought the total amino acid count to 261, as shown in Table 2. Model 3 utilized epitopes from CORE_REP Org10_Gene113# and included two B cell epitopes, one MHC I epitope, and three MHC II epitopes, totaling 257 amino acids (Table 3). Model 4 incorporated epitopes from Org2_Gene150# with the same linkage and adjuvant approach, yielding a construct of 293 amino acids (Table 4).

**Table 1:** MHC class I, class II, and B cell epitope predictions for Vaccine Model 1. The table includes Peptide sequences and their toxicity, allergenicity, and antigenicity.

Epitopes (CORE_REP Org2_Gene185#)	Peptides	Antigenicity	Allergenicity	Toxicity
B cell	GDYKVEEYCTG	0.9854	Non-Allergen	Non-Toxin
	LKYNYHFSDSSQITN	1.0409	Non-Allergen	Non-Toxin
MHC I	KIEWTGDGLY	1.2014	Non-Allergen	Non-Toxin
	SIHAKITSY	1.2853	Non-Allergen	Non-Toxin
	RSKEDVPNFK	0.6471	Non-Allergen	Non-Toxin
MHC II	KYNYHFSDSSQITNN	0.8948	Non-Allergen	Non-Toxin
	LKYNYHFSDSSQITN	1.0409	Non-Allergen	Non-Toxin
	YNYHFSDSSQITNNT	0.8236	Non-Allergen	Non-Toxin

**Table 2:** Predicted peptide sequences for MHC class I, class II, and B cell epitopes in Vaccine Model 2. The potential for antigenicity, allergenicity, and toxicity of each sequence was assessed.

Epitopes (CORE_REP Org6_Gene199#)	Peptides	Antigenicity	Allergenicity	Toxicity
B cell	DQKDYTVTSQFNNYTIG	1.0008	Non-Allergen	Non-Toxin
	PDGLDIPLT	0.6951	Non-Allergen	Non-Toxin
MHC I	VTMTYKNTK	1.7573	Non-Allergen	Non-Toxin
	SILGVSIECK	1.8785	Non-Allergen	Non-Toxin
	IPLTNITYW	0.9224	Non-Allergen	Non-Toxin
MHC II	ETDYTYLSNGGLP	0.8729	Non-Allergen	Non-Toxin
	TGETDYTYLSNGGLPA	0.8689	Non-Allergen	Non-Toxin
	GETDYTYLSNGGLP	0.8986	Non-Allergen	Non-Toxin

**Table 3:** B cell, MHC class I, and MHC class II epitopes were thoroughly analyzed for Vaccine Model 3. The antigenicity of the peptides is assessed, and evaluations of their toxicity and allergenicity are included to ensure the safety and effectiveness of vaccine development.

Epitopes (CORE_REP Org10_Gene113)	Peptides	Antigenicity	Allergenicity	Toxicity
B cell	TIALKHSGYSSELNDISIGLTPNDTIKE	1.233	Non-Allergen	Non-Toxin
	FKNKTDII	0.7983	Non-Allergen	Non-Toxin
MHC I	ELNDISIGL	2.5692	Non-Allergen	Non-Toxin
	TPFDVEDTF	1.4167	Non-Allergen	Non-Toxin
	LSFIGYMVK	1.1948	Non-Allergen	Non-Toxin
MHC II	FGYVPYVGYKCINH	1.3178	Non-Allergen	Non-Toxin
	GYVPYVGYKCINH	1.0597	Non-Allergen	Non-Toxin
	PYVGYKCINHVS	1.2610	Non-Allergen	Non-Toxin

**Table 4:** Peptide sequence predictions for Vaccine Model 4, displaying class I, class II, and B cell epitopes. To evaluate the immunogenic potential and safety of the vaccine candidate and to achieve a strong, non-allergic immune response, assessments of antigenicity, allergenicity, and toxicity are conducted.

Epitopes (Org2_Gene150#)	Peptides	Antigenicity	Allergenicity	Toxicity
B cell	YGNIKEFNATHAAFEYSKSIGGTPALDRRVQDVNDTISDVKQK	0.8069	Non-Allergen	Non-Toxin
	AEVGPNNTRSIRKFNTMRQC	0.1312	Non-Allergen	Non-Toxin
MHC I	SIFGFQAEV	0.4227	Non-Allergen	Non-Toxin
	ATHAAFEYK	0.8034	Non-Allergen	Non-Toxin
	FTFSDVINI	1.0007	Non-Allergen	Non-Toxin
MHC II	LSIFFIVVATAAVCLLFI	0.7240	Non-Allergen	Non-Toxin
	SIFFIVVATAAVCLLFIQ	0.7517	Non-Allergen	Non-Toxin
	AVCLLFIQSYSI	0.8065	Non-Allergen	Non-Toxin

### 3.5 Secondary structure and tertiary structure prediction

**Fig 3:**
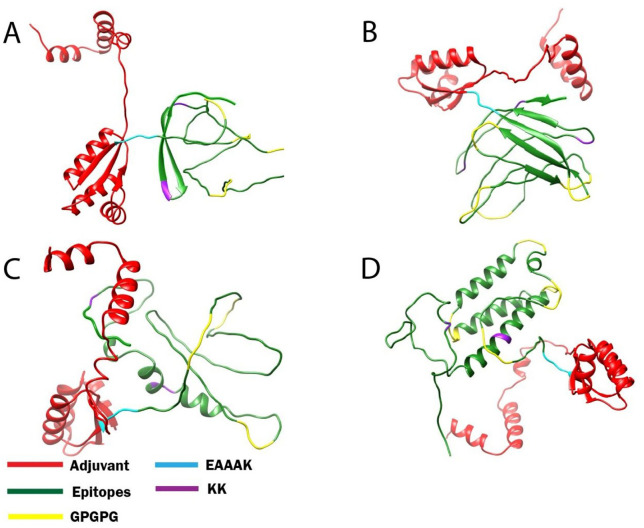
A representation of the vaccine structures for Vaccine Models 1-4. Models 1 to 4 are represented as **(A)**, **(B)**, **(C)**, and **(D)**. Important details are emphasized in various colors: Adjuvant areas appear in red, anticipated epitopes in forest green, GPGPG linker sequences in yellow, EAAAK linkers in cyan, and KK linkers in purple. The structural arrangement and composition of every vaccine model are illustrated by these color-coded properties.

The secondary structure of the candidate vaccine was predicted utilizing the advanced computational tool known as PSIPRED, which effectively identified several pivotal structural components integral to the molecular framework, including alpha-helices, beta-strands, and random coil regions. The alpha-helices and beta-strands are known to form stable regions that contribute to the overall rigidity and stability of the protein structure, whereas the coil regions serve a critical role as flexible linkers that facilitate the necessary conformational dynamics within the molecule (Table 5). The high confidence scores obtained during this predictive analysis significantly bolstered the credibility of these structural predictions, thereby establishing a solid and reliable foundation for subsequent analyses and investigations into the vaccine candidate’s properties. In the pursuit of elucidating the tertiary structure of the vaccine candidate, the cutting-edge software AlphaFold 2 was employed, which generated five distinct models of each vaccine that were subsequently ranked based on their respective confidence scores, specifically pLDDT values. We selected one model from each vaccine that exhibited high reliability, as evidenced by scores that consistently exceeded the pLDDT values threshold of 90. The 3D structure of those four designed vaccines was visualized using Chimera software and illustrated in (Fig 3A-3D).

**Table 5:** Percentage of Alpha helix, Beta sheet, and Random coil of designed vaccine models.

	Alpha helix	B-sheet	Random coil
Vaccine Model-1	33.33%	14.51%	52.16%
Vaccine Model-2	28.35%	19.16%	52.49%
Vaccine Model-3	36.58%	15.18%	48.25%
Vaccine Model-4	58.36%	9.56%	32.08%

### 3.6 Population Coverage of Predicted T-Cell Epitope

**Fig 4:**
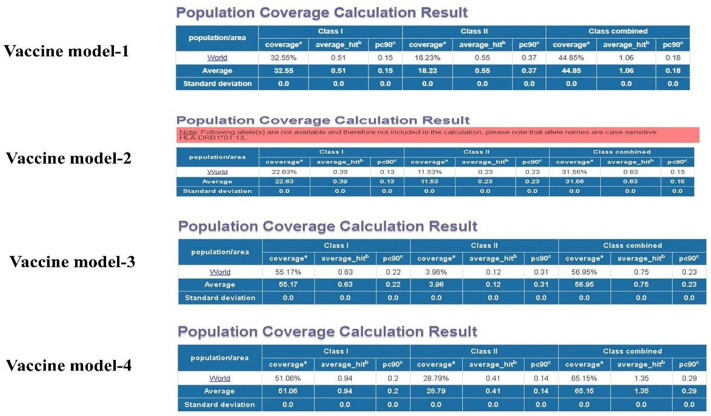
Population coverage results of all the designed vaccines.

In the context of natural biological systems, Major Histocompatibility Complex (MHC) molecules are found to be abundant and widely distributed across various communities and among multiple ethnic groups, highlighting their importance in the immune response. Consequently, the production of peptide-based vaccines is considered to be a highly effective strategy for the development of broad-spectrum vaccine candidates, particularly given the extensive array of MHC alleles present in diverse populations. The T cell epitopes identified in this comprehensive study revealed an average population coverage of 32.55% for MHC Class I, 18.23% for MHC Class II, and an impressive 44.85% when considering the combined coverage for the vaccine Model 1. In addition, the average population coverage for the vaccine Model 2 was calculated to be 22.63% for MHC Class I, 11.53% for MHC Class II, and 31.56% for combined coverage, thus indicating variability in the immune recognition patterns. Moreover, the vaccine Model 3 vaccine exhibited a remarkable average population coverage of 55.17% for MHC Class I, a considerably lower 3.96% for MHC Class II, and a combined coverage of 56.95%, underscoring the potential effectiveness of this vaccine model. Additionally, the vaccine Model 4 demonstrated an average population coverage of 51.06% for MHC Class I and a more substantial 28.79% for MHC Class II, culminating in an overall combined coverage of 65.15%, which suggests a robust ability to elicit an immune response across diverse populations. Fig 4 represents a comprehensive illustration of the known class I and class II HLA alleles alongside the percentage of epitope group coverage, thereby providing a visual representation of the findings and facilitating a deeper understanding of the potential efficacy of the vaccine candidates developed in this study.

### 3.7 Structure Refinement and Validation

**Fig 5:**
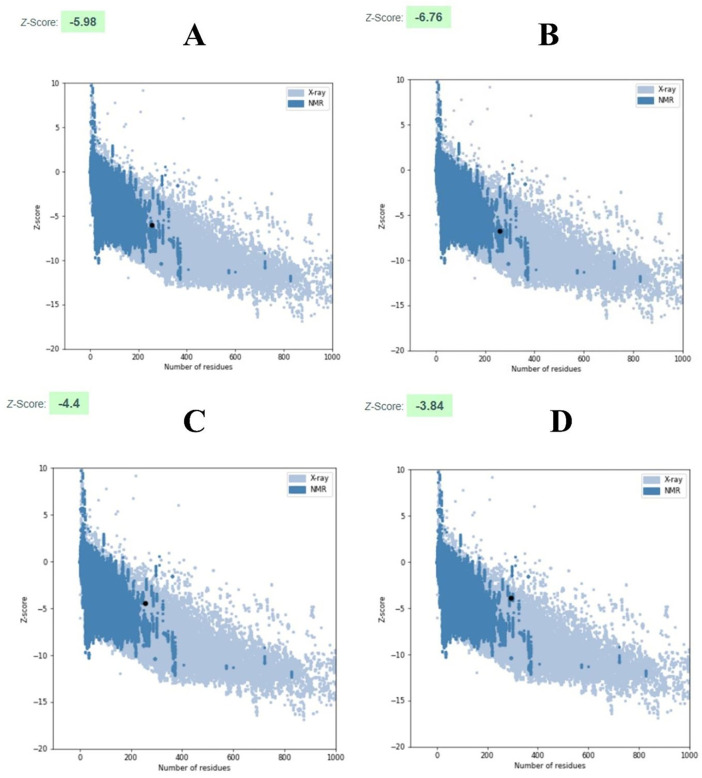
The generated result of all the Z scores for each vaccine model, where (A) represents Vaccine Model 1, (B) represents Vaccine Model 2, (C) represents Vaccine Model 3, and (D) represents Vaccine Model 4.

**Fig 6:**
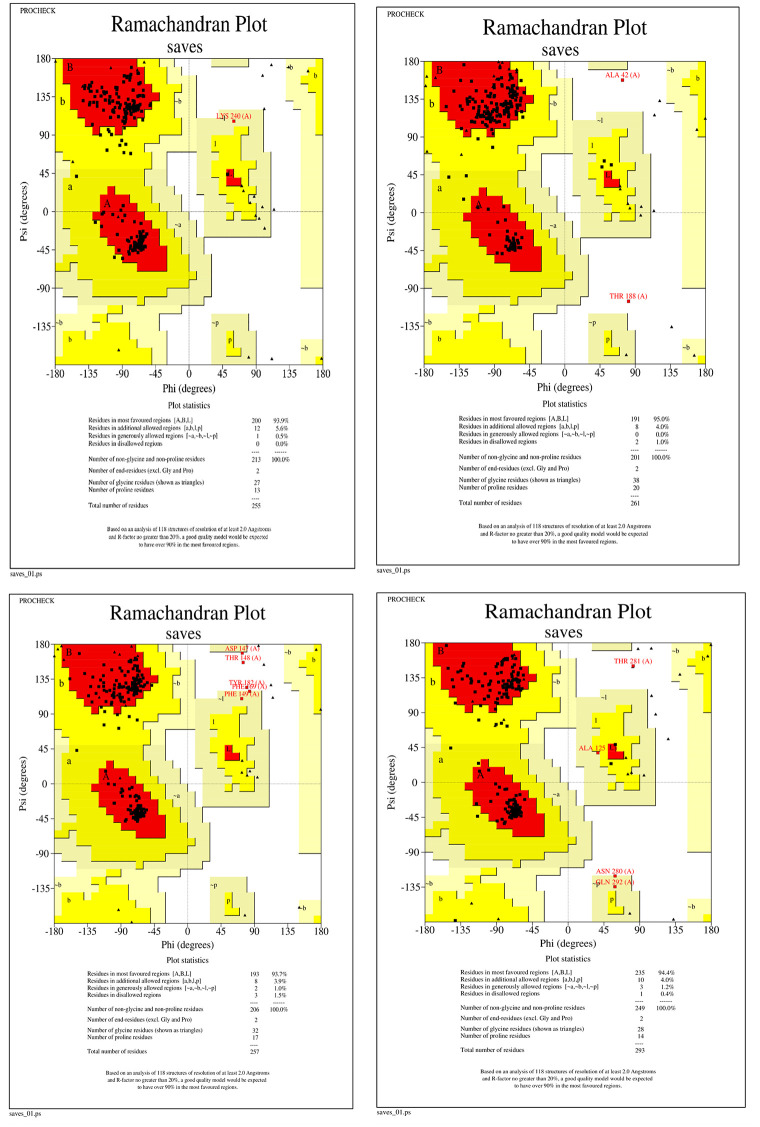
Graphical illustration of the Ramachandran plot of all the designed vaccine models. (**A**) represents Vaccine Model 1, (**B**) represents Vaccine Model 2, (**C**) represents Vaccine Model 3, and (**D**) represents Vaccine Model 4.

To enhance and refine the accuracy of the predicted three-dimensional model, the Galaxy Refine server was utilized, which is known for its ability to improve the precision of structural models by optimizing their geometric characteristics. The refinement process was particularly focused on enhancing the overall quality of the model, placing a significant emphasis on achieving proper folding and stabilization of the tertiary structure to ensure that it accurately reflects the natural conformations of proteins. Following this refinement, the model exhibited a marked improvement in accuracy, especially in regions that had initially been characterized by a lower degree of structural definition or stability. The refined three-dimensional model underwent a rigorous validation process using the ProSA-web tool, which provides z-scores indicative of the structural quality of the protein; the scores obtained were −5.98 for Vaccine model 1, −6.76 for Vaccine model 2, −4.4 for Vaccine model 3, and −3.84 for Vaccine model 4 each reflecting the overall quality and reliability of the protein structure (Fig 5A-5D). The z-scores achieved indicated that the refined structural model resided comfortably within the realm of typical high-quality protein models, thereby signifying that the predicted model was structurally sound and reliable for further investigations. Furthermore, the ProSA-web analysis revealed no significant energy anomalies within the structure, which serves as an additional confirmation of its stability and reliability. Lastly, the structural validation process was concluded utilizing the Ramachandran plot through the SAVES v.6.0 server, where more than 90% of the residues were found to reside within the favored regions of the plot in all the vaccine models, thus affirming the correctness of the phi and psi dihedral angles that are critical for proper protein folding (Fig 6A-6D). This validation process conclusively confirmed that the overall geometry of the model adhered to established structural norms and standards.

### 3.8 Disulphide Engineering and In-silico Codon Optimization

**Fig 7:**
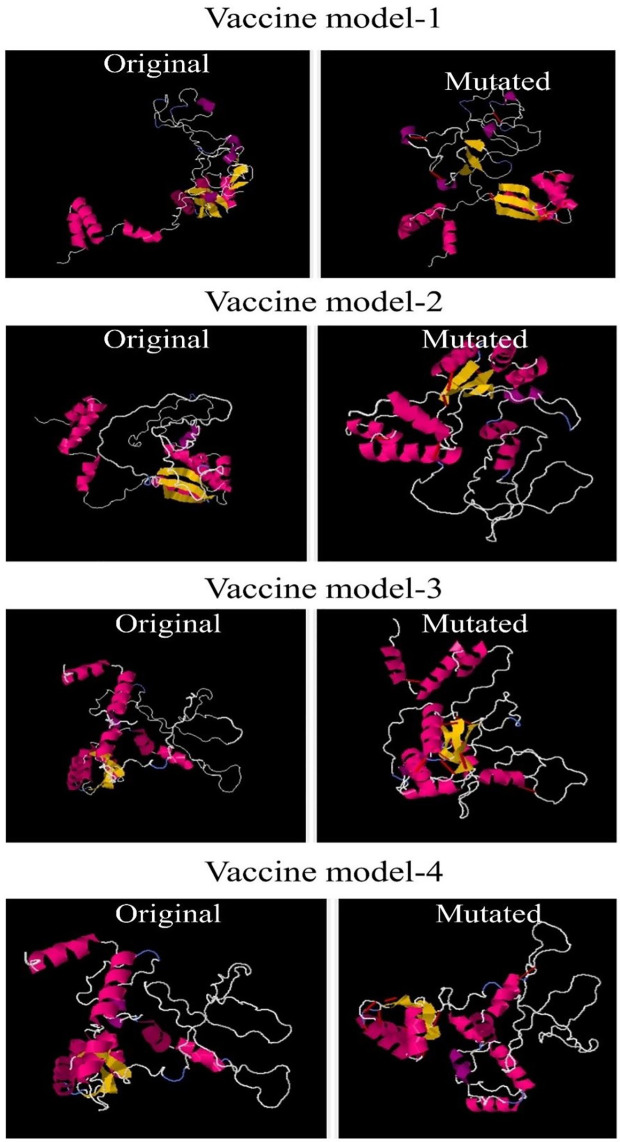
All the Left Panels show the designed vaccine models and all the right panels show a mutated version of the vaccine where red colored sticks represent newly added disulfide bonds.

The vaccine underwent disulfide engineering, which was meticulously designed to enhance and fortify the intermolecular bonding characteristics of the vaccine, thereby significantly improving the overall structural stability of the vaccine formulation. This intricate process was critical in ensuring that the more fragile segments of the vaccine were rendered more resilient against potential cellular degradation, consequently imparting a higher degree of conformational stability to the vaccine's architecture. Throughout the rigorous analytical assessment, only those residue pairs that exhibited a higher energy value, specifically those exceeding 0 kcal/mol, were selected for the mutation to cysteine, ensuring that only the most energetically favorable modifications were made (S6 File). The specific amino acid residues that underwent replacement by cysteine have been systematically tabulated for clarity and ease of understanding, while red sticks visually represent the resultant cysteine bonds formed as a result of this strategic alteration in the accompanying diagrams (Fig 7). This meticulous approach not only elucidates the importance of disulfide bonds in maintaining vaccine integrity but also underscores the intricate relationship between molecular structure and functional efficacy in vaccine design.

### 3.9. Molecular Docking Results of Vaccine Models with TLR-4

**Fig 8:**
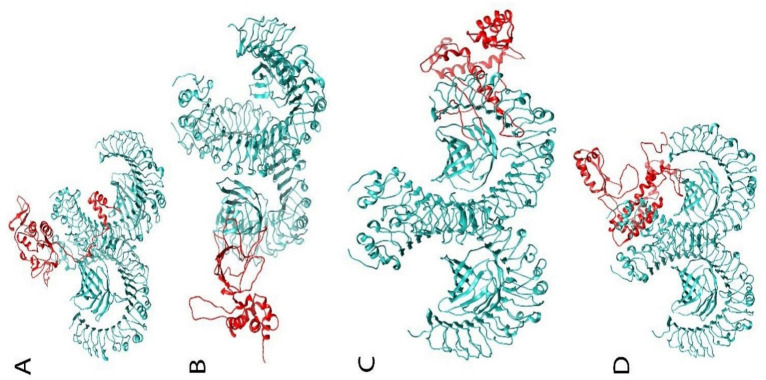
Vaccine-TLR4 receptor complex models for Vaccine Models 1–4. Models from 1 to 4 are represented by letters A to **D.** The TLR4 receptor is depicted in light sea green in each combination, whereas the vaccine is displayed in red. The spatial conformation of the complex is depicted, which show the 3D structure of the vaccine-receptor binding interaction.

The vaccine proteins’ interaction with TLR-4 was assessed using the Cluspro service to determine the possible immune response stimulation. Protein-protein docking simulations yielded 29 clusters for each vaccine model-TLR4 complex. The top-ranked docked clusters were chosen based on their cluster size and lowest energy scores, suggesting a better binding affinity and interaction stability between the vaccine models and TLR-4. According to the docking simulation, vaccine model 1 has a center energy and a lowest energy of −799 kcal/mol, which suggests moderate stability and potential for interaction with TLR-4 (Fig 8A, Table 6). With a center energy of −946.2 Kcal/mol and a lowest energy of −1123.3 Kcal/mol, vaccine model 2 appeared to have a higher binding affinity for TLR-4 than vaccine model 1(Fig 8B, Table 7). The docking findings for vaccine model 3 showed that its center energy was −1025.6 Kcal/mol, and its lowest energy was −1030.9 Kcal/mol. This indicates that vaccine model 3 has the highest binding affinity to TLR-4 and is the most stable interaction among the models examined (Fig 8C, Table 8). In comparison to vaccine model 3, vaccine model 4 showed a somewhat less robust center energy of −958.4 kcal/mol, with the lowest energy below −1068 kcal/mol, indicating a steady interaction (Fig 8D, Table 9).

**Table 6:** The interaction of hydrophilic and hydrophobic bonds between the TLR4 receptor and Vaccine model 1.

Types	TLR4(Chain B)	Vaccine model 1
Hydrophilic	Thr514, His458, Gln484, Arg460, Lys541, Ser482, rg606, Arg382, Thr359, Tyr403, Asp428, Asp405	Lys101, Ser34, Ala42, Asp102, Ala35, Ser16, Ile3, Met1, Ser2, Lys5
Hydrophobic	Pro513, Ser512, Gln505, Leu553, His431, His456, Ala462, Phe538, Glu484, Asp550, Ser552, Gln507, Asn575, Asn481, Gly480, Glu603, Phe408, Gln578, Ser360, Val338, Asp379, Thr357, His426	Glu51, Ile24, Val21, Met27, Vl33, Gly32, Ala38, Val46, Ala43, Gly44, Val39, Val39, Vl17, Phe31, Met18

**Table 7:** The interaction of hydrophilic and hydrophobic bonds between the TLR4 receptor and Vaccine model 2.

Types	TLR4(Chain C)	Vaccine model 2
Hydrophilic	Gln156, Gln41, Ser45, Ser27, Asn26, Lys58, Trp23, Lys20, Ser28, Tyr34	Thr188, Ser141, Tyr163, Thr191, Pro205, Tyr193, Thr162, Asn160, Glu190, Asn178
Hydrophobic	Met40, Lys39, Leu60, Gly59, Phe64, His62, Asn49, Pro50, Cys25, Val24, Pro43, Tyr22	Pro186, Pro139, Gly138, Pro137, Ala203, Asp192, Tyr175, Pro166, Gly167, Pro202, Leu201, Gly204, Gly179, Gly183, Leu181, Gly180, Hel142, Gly165, Gly189, Trp164

**Table 8:** The interaction of hydrophilic and hydrophobic bonds between TLR4 receptor and Vaccine model 3.

Types	TLR4(Chain C)	Vaccine model 3
Hydrophilic	Asn49, Asn47, Ser45, Val24, Trp23, Lys20	Pro167, Gly168, Phe169, Tyr171, Lys163, Tyr160, Asp147, Glu146
Hydrophobic	Asn114, Ser103, Thr115, Phe64, Ile44, Tyr22, Gln19, Lys39, Gln41, Met40	Gly203, Pro204, Tyr207, Pro173, Gly170, Met161, Gly164, Pro165, Thr148, Gly152, Val145, Gly150, Pro153

**Table 9:** The interaction of hydrophilic and hydrophobic bonds between the TLR4 receptor and Vaccine model 4.

Types	TLR4(Chain B)	Vaccine model 4.
Hydrophilic	Lys20, Gln21, Glu144, Pro142, Lys91, Glu136, Lys89	Lys5, Asp6, Arg257, Thr251, Gly250, Gly249
Hydrophobic	Glu143, Tyr75, Lys39, Met145, Ile138, Asn77, Tyr79	Ser247, Asp255, Ala253, Ile248

### 3.10 Molecular Dynamics Simulation

A potent computational method for examining the dynamic behavior and structural integrity of atoms and molecules at the atomic scale is molecular dynamics simulations, or MDS. When used in vaccine design, MD simulations offer vital information on the stability and binding affinity of a vaccine candidate as well as how it interacts with its target protein. The intensity and longevity of these interactions over time can be assessed by mimicking the vaccine's binding to the active site of a protein. MDS makes it possible to thoroughly examine how well a vaccine binds to its target protein. The complex's overall conformational stability and flexibility are measured using important measures such as root mean square deviation (RMSD) and root mean square fluctuation (RMSF). To determine whether the binding is stable or prone to dissociation, RMSD shows how the protein-ligand complex's structure changes from its starting state throughout the simulation. Conversely, RMSF identifies parts of the protein that have a great deal of flexibility and may impact ligand binding. Furthermore, assessing secondary structure elements (SSEs), which are essential to the structural stability of proteins and include α-helices, β-sheets, and loops, is a crucial component of MDS. It is possible to evaluate how vaccine binding impacts the protein's secondary structure and whether notable changes occur by monitoring the SSE during simulations. Strong ligand binding is frequently indicated by stability in these secondary structures, while instability or weak connections may be indicated by disruptions or unfolding.

**Fig 9:**
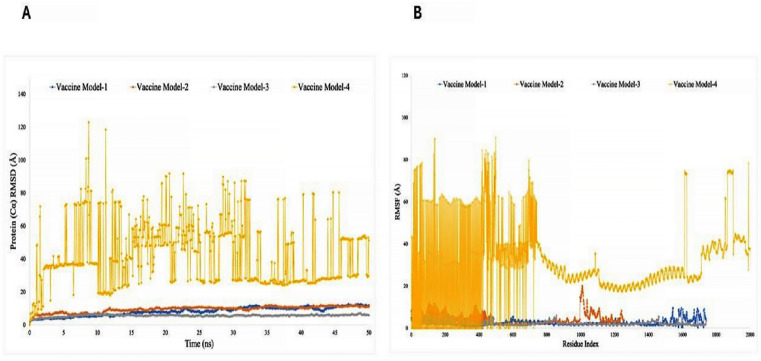
RMSD and RMSF of the vaccine model-1, Model-2, Model-3, and Model-4. Vaccine Models 1, 2, and 3 exhibit low, plateauing RMSD values (below 20 Å), indicating high structural stability and convergence. In contrast, Vaccine Model 4 (yellow) shows extreme fluctuations and high RMSD values, suggesting significant structural instability or unfolding during the simulation **(A)**. While Models 1-3 show minimal fluctuations across the residue index, Vaccine Model 4 demonstrates exceptionally high RMSF values, particularly in the N-terminal and internal loop regions, indicating highly mobile and disordered segments **(B)**.

During the 50 ns molecular dynamics simulation, the Root Mean Square Deviation (RMSD) is an essential metric for assessing the vaccination models’ structural integrity. An RMSD value between 1 and 5 Å is typically seen as a sign of high stability, indicating little structural variation from the original shape. Throughout the simulation, Vaccine Model 1 continuously kept its RMSD values between 1.5 Å and 2.5 Å. This narrow fluctuation range demonstrates the model's resilience and implies that it underwent few conformational changes, maintaining its structural integrity throughout time. Similar trends were seen in Vaccine Model 2, where RMSD values varied from 2 Å to 4 Å, occasionally surpassing the 3 Å threshold while being within stability-acceptable bounds. Although these variations point to minor variations, they are not substantial enough to imply structural instability. Vaccine Model 3 also displayed a steady RMSD profile, with values falling between 2 Å and 3.5 Å. This implies that during the simulation, Model 3, like Models 1 and 2, maintained its structural stability with little conformational changes. Model 3's RMSD values are within a stable range, albeit being marginally greater than Model 1's, suggesting that it is similarly stable. Moreover, Vaccine Model 4 also showed significant RMSD variations, which is also acceptable (Fig 9A-9B).

**Fig 10:**
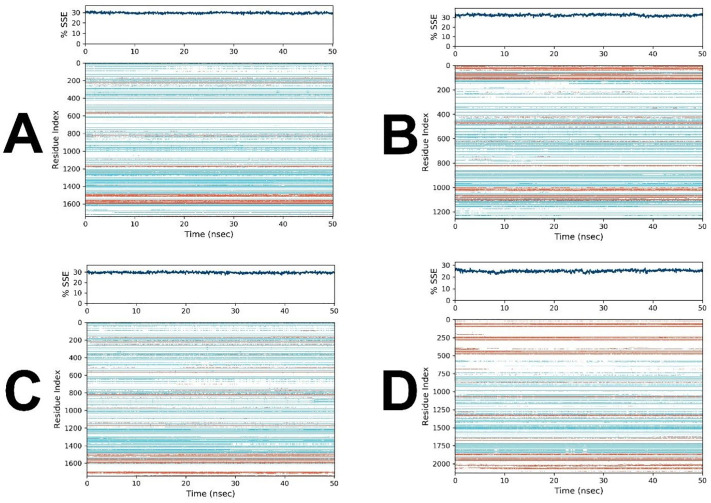
Secondary Structure Element analysis of A). vaccine model-1, B). vaccine Model-2, C). Vaccine Model-3, and **D).** Vaccine Model-4. Upper Panels (% SSE): Display the total percentage of secondary structural elements (helices and strands) maintained over the simulation time. All models show a steady percentage (approximately 25%–30%), indicating that the overall secondary structural composition remains globally consistent throughout the trajectory. Lower Panels (Heatmaps): Provide a per-residue breakdown of secondary structure over time. Residue Index (y-axis): Tracks specific amino acid positions within the construct. Typically, orange/red horizontal bands represent alpha-helices, while light blue/teal bands represent beta-strands. The persistence of solid horizontal bands across the 50 ns time scale (x-axis) confirms that the local folds and specific structural motifs of each vaccine candidate are stable and do not undergo significant denaturation or unfolding under the simulated physiological conditions.

During the simulation, the flexibility of each particular residue in the protein structure is measured by Root Mean Square Fluctuation (RMSF). Greater flexibility or instability is suggested by larger RMSF values, whereas more stiff parts are indicated by lower RMSF values (usually less than 1–2 Å). Vaccine Models 1 and 2 showed comparatively low changes across the majority of residues in the RMSF analysis for the vaccine models. The fact that most of the residues stayed below 2 Å suggests that these models had a rigid core structure with little room for flexibility. Small peaks surrounding residues in particular areas (maybe loop or surface-exposed regions) were more than 2 Å, but they did not exhibit significant variation and remained well within acceptable bounds for protein stability. These peaks most likely correspond to areas of flexible loops that preserve the models’ overall structural integrity. A similar pattern was seen in Vaccine Model 3, where the majority of the residues had RMSF values less than 2 Å. The general pattern indicates that Model 3 likewise maintains a decent level of structural stability, with only a few flexible locations contributing to higher RMSF values, even though some places displayed slightly larger fluctuations than Models 1 and 2. Vaccine Model 4 showed greater oscillations in some areas, with RMSF values surpassing 5 Å and in some areas even surpassing 8 Å. Particularly in areas that are probably exposed to the surface or engaged in dynamic interactions, these notable peaks may imply localized flexibility and structural instability. The overall conformation of the protein may not become unstable as a result of this degree of variation. Proteins’ total three-dimensional conformation depends on secondary structure elements (SSE), and their persistence over time is an indication of structural stability. With just minor alterations in the ratios of α-helices and β-sheets, Vaccine Model 1 continuously maintained more than 25% of its original secondary structure over the simulation. Similar to Model 1, Vaccine Model 2 retained more than 20% of its secondary structure, albeit with a few small variations, suggesting a minimal loss in structural integrity. Conversely, after 30 ns, Vaccine Model 3's SSE stability gradually decreased, indicating a noticeable loss of secondary structure. Due to its minor substantial RMSD and RMSF swings, Vaccine Model 4 showed a minor loss, with SSE retention falling below 10% early in the experiment. These findings demonstrate that Models 1, Model 2, Models 3, and Model 4 all are structurally stable and can turn out as stable candidate (Fig 10A-10D). Additionally, COCOMAPS 2.0 enables the examination and mapping of protein complex interfaces by identifying critical hot spot residues through intermolecular contact data. Both the RMSD results and the temporal interaction maps (S7, S8, S9, S10, S11, S12, S13, S14, and S15 File) demonstrate a consistent pattern of contact, showing that the interface remained robust throughout the simulation. Collectively, these results confirm that the binding between the vaccine construct and TLR4 is highly stable.

### 3.11 Immune Response Simulation

The experimental models of the immune response exhibited parallels to the genuine immunological occurrences triggered by specific pathogens, as illustrated in the S16 File. Notably, the secondary and tertiary immune responses of all the selected vaccine model candidates were significantly more vigorous than the primary immune response. The secondary and tertiary responses were defined by elevated levels of various antibody types (including IgG1 + IgG2, IgM, and IgG + IgM), which coincided with a reduction in the antigen load, thereby indicating the formation of memory cells and the subsequent enhancement of antigen elimination during subsequent exposures. Furthermore, an increased lifespan was noted in B-cells, cytotoxic T-cells, and helper T-cells, suggesting a transition among immune cells and the establishment of IgM memory. It was also clear that IFN, IL-4, and IL-10 were all overexpressed. It's interesting to note that compared to Th1-type immune reactions, Th0-type immune responses had lower percentages (%) and quantities (cells/mm3). Throughout the presentation, it was shown that although dendritic cell movement was determined to be predictable, macrophage mobility increased dramatically.

## 4. Discussion

The need for a suitable vaccination against the MPXV is urgent, given the increasing number of children and immunocompromised individuals who die from it worldwide. Because of its changing pathogenic characteristics, the lack of effective vaccinations or treatment alternatives against MPXV has created serious risks. This genetic evolution has been a significant driver of the recent outbreak, with phylogenomic studies confirming a substantial number of mutations in the core genome of newly emerged isolates [46]. The most effective and economical way to stop virus outbreaks is through vaccination. Inducing humoral and cell-mediated immunity against the target virally infected cells should be a feature of an effective vaccination [47]. This integrated “pan-genome and reverse vaccinology” pipeline aligns our work with a powerful and validated trend in modern vaccinology. This exact approach has been successfully leveraged to design promising vaccine candidates against other genetically complex viruses, including Human Cytomegalovirus (HCMV) [48], Herpes Simplex Virus type-1 (HSV-1) [49], and Epstein-Barr virus (EBV) [50], as well as other pathogens like the Nipah virus [51]. Hence, a genome-based reverse vaccinology approach was adopted to design extensively validated multi-epitopic vaccine candidates that can efficiently provide immunity against MPXV infections.

Numerous studies have explored the use of immunoinformatics as a first-line vaccine design technique, contributing to the development of effective multi-epitopic MPXV vaccines. But in our study, we utilized recently published complete sequence data derived from different parts of the world since the 2022 outbreak. Pan-genome-based screening removes the constraints (biases) of data obtained from secondary/curated sources and confirms the global homogeneity of vaccine targets in MPXV. Potential target proteins were selected from annotated complete genomes of MPXV based on their solubility, antigenicity, outer-membrane position, and heterology to human proteins. While eliciting immunological responses, the conservation qualities guaranteed little cross-reactivity. Pathogen surface proteins, of course, have a higher chance of interacting with the host's immune system and triggering an immunological response [52]. At the in vitro and in vivo levels, the vaccinia virus membrane proteins have previously been assessed as a target of neutralizing antibodies and as a component of the subunit vaccine [53]. To anticipate putative T-cell and B-cell epitopes and create a multi-peptide-based vaccination that can elicit a robust CD4+ and CD8 + T-cell-related immunological response, recent immunoinformatics research targeted on the E8L, B6R, and SERP2 proteins of MPXV [54,55]. However, other MPXV proteins that have an exposed position might potentially be viable options for vaccine development. To identify immunodominant T-cell and B-cell epitopes and create a multi-epitope vaccine that can cross-protect against different strains of the virus causing the current outbreak, we examined all membrane-bound, enveloped, and extracellular proteins of MPXV and chose 4 proteins using a reverse vaccinology pipeline. Consequently, MPXV's outer-membrane proteins were refined to extract MHC class I T-cell epitopes, MHC class II T-cell epitopes, and B-cell epitopes, which, upon binding, might elicit potent immunological responses. Several server-based estimates confirmed the same. To improve efficacy, the final vaccine constructs were created by aligning many epitopes and joining them with adjuvants, GPGPG, and KK linkers. The vaccine may stimulate and activate a more robust protective immune response in recipients due to the adjuvants used in its formulation. All these constructs were screened for antigenicity, allergenicity, and toxicity in silico to validate their safety profile leading to developmental efficacy. The computational modeling and structural predictions of the vaccine candidates gave us valuable insights into their stability and immunogenicity. Using AlphaFold, the 3D structures of these vaccines were generated and Chimera was used to visualize them. The predicted stable and well-folded structures of the selected vaccine models implied that these models were necessary to guarantee sufficient immune recognition. Population coverage of the selected epitopes revealed variation in immune response related to different MHC alleles, indicating the need for a vaccine design that can induce broad immune responses across diversified populations. Vaccine Model 1, combining the highest coverage for MHC Class I and II alleles, in particular, holds promise for effective immune response in a wide range of individuals. Together with other models, this gave a full framework for developing a vaccine that could conceivably protect against MPXV in numerous populations around the world. Molecular dynamics simulations were performed further to confirm the structural stability of the vaccine candidates. Indeed, stability was maintained for Models 1 and 2 in simulation, indicating that these might potentially withstand physiological conditions. By contrast, larger conformational changes were observed with Vaccine Model 4. The molecular docking of the vaccine models with TLR-4 showed that the vaccine candidates have promising interactions with this important receptor involved in the initiation of innate immune responses, thus increasing the likelihood of stimulating a strong immune response. Finally, disulfide engineering and in-silico codon optimization of the vaccine constructs further enhanced the structural stability and expression efficiency of the latter. In this respect, disulfide bonds were used to stabilize the proteins, and hence functional integrity upon expression was ensured. Codon optimization has been done to increase the expression of the vaccine in an appropriate host system, which may produce the vaccine in considerable amounts required for further preclinical and clinical trials.

Considering the developed vaccine contains core epitopes, which may provide broad-spectrum immune protection against a range of pathogen types, it has significant clinical promise. Its computational study also indicates that it has a good safety profile because it is non-toxic and non-allergic. However, to enhance its translational effectiveness, multiple limitations require to be addressed in further research. The most effective epitope combination inside the vaccine design that generates a potent and persistent immune response must initially be identified through experimental validation. Secondly, continuous improvements in MHC epitope prediction algorithms will enhance the selection of highly immunogenic epitopes by further improving antigen presentation accuracy. Finally, comprehensive in vitro and in vivo studies such as immunogenicity evaluations, animal challenge studies, and clinical trials, are required to validate the vaccine's long-term safety and protective effectiveness. It will be possible to develop a therapeutically feasible vaccine candidate with significant immunoprotective potential if these limitations are addressed.

## 5. Conclusions

The current study designs a multi-faceted approach for the development of a multi-epitope vaccine against MPXV. We combined the use of bioinformatics tools and experiments for the identification of potential vaccine candidates that could generate strong immune responses. Results obtained herein provide a basic contribution to the development of an effective vaccine against Monkeypox that could have a very crucial effect on this emergent and re-emergent global threat. Due to this fact, further testing of vaccine candidates in preclinical and clinical settings is imperative for confirmation of their safety and efficacy profile.

## Supporting information

S1 File Roary_CoreGenome_170Genes_Analysis. (CSV)

S2 File PanGenomeAnalysis_Roary_BPGA_CoreGeneComparison. (XLS)

S3 File CoreProteins_BPGA180_NonHuman135_Membrane30. (XLSX)

S4 File AntigenicProteins_BPGA16_ValidatedTMH. (XLSX)

S5 File EpitopePrediction_FinalCandidates4_BPGA. (RAR)

S6 File DisulfideEngineering_VaccineStabilityAnalysis. (XLSX)

S7 File Results_for_Vaccine_Model_1_chain_A_vs_X. (RAR)

S8 File Results_for_Vaccine_Model_1_chain_B_vs_X. (RAR)

S9 File Results_for_Vaccine_Model_1_chain_C_vs_X. (RAR)

S10 File Results_for_Vaccine_Model_1_chain_D_vs_X. (RAR)

S11 File Results_for_Vaccine_Model_2_chain_C_vs_X. (RAR)

S12 File Results_for_Vaccine_Model_3_chain_C_vs_A. (RAR)

S13 File Results_for_Vaccine_Model_4_chain_A_vs_X. (RAR)

S14 File Results_for_Vaccine_Model_4_chain_B_vs_X. (RAR)

S15 File Results_for_Vaccine_Model_4_chain_C_vs_X. (RAR)

S16 File ImmuneResponseSimulation_ParallelsToPathogens. (RAR)
