# Supplementary material for: Pan-genome and reverse vaccinology for a multi-epitope vaccine against circulating post-2022 Monkeypox virus strains
Source: PLoS One. 2026 Jun 22;21(6):e0351881. doi: 10.1371/journal.pone.0351881 (PMC13286271; doi:10.1371/journal.pone.0351881)
Supplement: Attachment — Submitted filename: PONE-D-25-33735.pdf [file pone.0351881.s017.pdf]

PLOS ONE

Pan-Genome and Reverse Vaccinology for a Multi-Epitope Vaccine Against Circulating Post-2022 Monkeypox Virus Strains

--Manuscript Draft--

**Manuscript Number:**	PONE-D-25-33735
**Article Type:**	Research Article
**Full Title:**	Pan-Genome and Reverse Vaccinology for a Multi-Epitope Vaccine Against Circulating Post-2022 Monkeypox Virus Strains
**Short Title:**	Pan-Genome and Reverse Vaccinology for a Multi-Epitope Vaccine Against Circulating Post-2022 MPOX strains
**Corresponding Author:**	Md. Nazmul Nazmul Hasan (Ph.D)<br>Jashore University of Science and Technology<br>Jashore, BANGLADESH
**Keywords:**	
**Abstract:**	Background<br><br>Since the 2022 outbreak, Monekypox's worldwide total cases have been increasing. As of August 2024, the viral infections have infected around 99,000 people overall. Particularly, the World Health Organization (WHO) categorized this Mpox virus as a Public Health Emergency of International Concern (PHEIC) based on the clade I of the virus, which have raised fatality rate (arround 3.6%).<br><br>Objectives<br><br>Our goal was to Computationally creat and evaluate a broad-spectrum multi-epitope vaccine that can elicit humoral and adaptive immune responses against MPXV infection, targeting the virus's membrane-bound, enveloped, and extracellular proteins.The tertiary structure of the chimeric vaccine construct's interaction with toll like receptor 4 (TLR4) was evaluated through the use of molecular docking and molecular dynamic modeling.<br><br>Methods<br><br>During an outbreak emergency, a pan-genome-based reverse vaccinology technique can offer quick and effective fixes for enduring problems with experimental vaccine design. In order to find viral targets, the method involved screening 16 monkeypox entire genomes. Viral proteins were selected among screened viral targets according to their antigenicity, location, and solubility. Immunoinformatics methods and algorithms were used to extract the proteins' putative T-cell and B-cell epitopes. The epitopes were combined to create several vaccine constructions. Using molecular docking and molecular dynamics simulation, the tertiary structure of the chimeric vaccine construct's interaction with Toll-like receptor 4 (TLR4) was assessed.<br><br>Results<br><br>80 core genes were identified by pan-genomic screening, and they were then further screened for appropriate epitope-designing proteins. We created four vaccination constructs by extracting T-cell and B-cell epitopes from four proteins. Adjuvants and linkers that were appropriate were added to the constructions. The stability and immunogenicity of every vaccine design were demonstrating encouraging outcomes. As a result, the vaccine designs might be effective in preventing monkeypox, which would support further testing and similar strategies to combat other viral illnesses.
**Order of Authors:**	Md Al Saber
	Md. Hasan Jafre Shovon
	Md. Imtiaz

Powered by Editorial Manager® and ProduXion Manager® from Aries Systems Corporation

	Sadia Jannat Tauhida
	Sowmitro Das
	Md Mohaimenul Islam Tareq
	Miss Ismoth Ara Tripty
	Md Ridoy Hossain
	Labib Shahriar Siam
	Saifullah Saifullah
	Md Nazmul Hasan Zilani
	Umama Khan
	S.M. Khaledur Rahman
	Md Nazmul Hasan
**Additional Information:**	
**Question**	**Response**
**Financial Disclosure**<br><br>Enter a financial disclosure statement that describes the sources of funding for the work included in this submission. Review the submission guidelines for detailed requirements. View published research articles from *PLOS ONE* for specific examples.<br><br>This statement is required for submission and **will appear in the published article** if the submission is accepted. Please make sure it is accurate.<br><br>**Funded studies**<br>Enter a statement with the following details:<br>• Initials of the authors who received each award<br>• Grant numbers awarded to each author<br>• The full name of each funder<br>• URL of each funder website<br>• Did the sponsors or funders play any role in the study design, data collection and analysis, decision to publish, or preparation of the manuscript?<br><br>Did you receive funding for this work?	The author(s) received no specific funding for this work.
**Competing Interests**<br><br>Use the instructions below to enter a	The authors have declared that no competing interests exist

competing interest statement for this submission. On behalf of all authors, disclose any competing interests that could be perceived to bias this work—acknowledging all financial support and any other relevant financial or non-financial competing interests.<br><br>This statement is required for submission and **will appear in the published article** if the submission is accepted. Please make sure it is accurate and that any funding sources listed in your Funding Information later in the submission form are also declared in your Financial Disclosure statement.<br><br>View published research articles from *PLOS ONE* for specific examples.<br><br>**NO authors have competing interests**<br><br>Enter: *The authors have declared that no competing interests exist.*<br><br>**Authors with competing interests**<br><br>Enter competing interest details beginning with this statement:<br><br>*I have read the journal's policy and the authors of this manuscript have the following competing interests: [insert competing interests here]*<br><br>* typeset	
**Ethics Statement**<br><br>Enter an ethics statement for this submission. This statement is required if the study involved:<br><br>• Human participants<br>• Human specimens or tissue<br>• Vertebrate animals or cephalopods<br>• Vertebrate embryos or tissues<br>• Field research	N/A

Write "N/A" if the submission does not require an ethics statement.

General guidance is provided below. Consult the submission guidelines for detailed instructions. **Make sure that all information entered here is included in the Methods section of the manuscript.**

Format for specific study types

Human Subject Research (involving human participants and/or tissue)

- Give the name of the institutional review board or ethics committee that approved the study
- Include the approval number and/or a statement indicating approval of this research
- Indicate the form of consent obtained (written/oral) or the reason that consent was not obtained (e.g. the data were analyzed anonymously)

Animal Research (involving vertebrate animals, embryos or tissues)

- Provide the name of the Institutional Animal Care and Use Committee (IACUC) or other relevant ethics board that reviewed the study protocol, and indicate whether they approved this research or granted a formal waiver of ethical approval
- Include an approval number if one was obtained
- If the study involved *non-human primates*, add *additional details* about animal welfare and steps taken to ameliorate suffering
- If anesthesia, euthanasia, or any kind of animal sacrifice is part of the study, include briefly which substances and/or methods were applied

Field Research

Include the following details if this study involves the collection of plant, animal, or other materials from a natural setting:

- Field permit number
- Name of the institution or relevant body that granted permission

**Data Availability**<br><br>Authors are required to make all data underlying the findings described fully available, without restriction, and from the time of publication. PLOS allows rare exceptions to address legal and ethical concerns. See the PLOS Data Policy and FAQ for detailed information.<br><br>A Data Availability Statement describing where the data can be found is required at submission. Your answers to this question constitute the Data Availability Statement and **will be published in the article**, if accepted.<br><br>**Important:** Stating 'data available on request from the author' is not sufficient. If your data are only available upon request, select 'No' for the first question and explain your exceptional situation in the text box.<br><br>Do the authors confirm that all data underlying the findings described in their manuscript are fully available without restriction?	Yes - all data are fully available without restriction
**Describe where the data may be found in full sentences. If you are copying our sample text, replace any instances of XXX with the appropriate details.**<br><br>• If the data are **held or will be held in a public repository**, include URLs, accession numbers or DOIs. If this information will only be available after acceptance, indicate this by ticking the box below. For example: *All XXX files are available from the XXX database (accession number(s) XXX, XXX.*).<br>• If the data are all contained **within the manuscript and/or Supporting Information files**, enter the following: *All relevant data are within the manuscript and its Supporting Information files.*<br>• If neither of these applies but you are able to provide **details of access elsewhere**, with or without limitations, please do so. For example:	All relevant data are within the manuscript and its Supporting Information files.

Data cannot be shared publicly because of [XXX]. Data are available from the XXX Institutional Data Access / Ethics Committee (contact via XXX) for researchers who meet the criteria for access to confidential data.<br><br>*The data underlying the results presented in the study are available from (include the name of the third party and contact information or URL).*<br>• This text is appropriate if the data are owned by a third party and authors do not have permission to share the data.<br><br>* typeset	
Additional data availability information:	

Powered by Editorial Manager® and ProduXion Manager® from Aries Systems Corporation

Manuscript
Click here to access/download;Manuscript;correction_manuscript - Final.docx

Pan-Genome and Reverse Vaccinology for a Multi-Epitope Vaccine Against Circulating Post-2022 Monkeypox Virus Strains

Md Al Saber[1], Md. Hasan Jafre Shovon[1], Md. Imtiaz[1], Sadia Jannat Tauhida[1], Sowmitro Das[1], Md Mohaimenul Islam Tareq[1], Miss Ismoth Ara Tripty[1], Md Ridoy Hossain[1], Labib Shahriar Siam[1], Md Nazmul Hasan Zilani[2], Saifullah[1], S.M. Khaledur Rahman[1], Umama Khan[1], Md Nazmul Hasan[1]

[1]Laboratory of Pharmaceutical Biotechnology and Bioinformatics, Department of Genetic Engineering and Biotechnology, Jashore University of Science and Technology, Jashore 7408, Bangladesh.

[2]Department of Pharmacy, Faculty of Biological Science and Technology, Jashore University of Science and Technology, Jashore 7408, Bangladesh.

***Correspondence:** mn.hasan@just.edu.bd (Md. Nazmul Hasan)

Abstract:

Background

Since the 2022 outbreak, the total cases of Monkeypox worldwide have been increasing. As of August 2024, viral infections have infected around 99,000 people overall. Particularly, the World Health Organization (WHO) categorized this Mpox virus as a Public Health Emergency of International Concern (PHEIC) based on the clade I of the virus, which has raised the fatality rate (around 3.6%).

Objectives

Targeting the membrane-bound, enveloped, and extracellular proteins of the virus, our goal was to computationally develop and assess a broad-spectrum multi-epitope vaccine that can elicit humoral and adaptive immune responses against Monkeypox virus (MPXV) infection.

Methods

During an outbreak emergency, a pan-genome-based reverse vaccinology technique can offer quick and effective fixes for enduring problems with experimental vaccine design. To find viral

targets, the method involved screening 16 monkeypox entire genomes. Viral proteins were selected among screened viral targets according to their antigenicity, location, and solubility. Immunoinformatics methods and algorithms were used to extract the proteins' putative T-cell and B-cell epitopes. The epitopes were combined to create several vaccine constructions. Using molecular docking and molecular dynamics simulation, the tertiary structure of the chimeric vaccine construct's interaction with Toll-like receptor 4 (TLR4) was assessed.

Results

80 core genes were identified by pan-genomic screening, and they were then further screened for appropriate epitope-designing proteins. We created four vaccination constructs by extracting T-cell and B-cell epitopes from four proteins. Adjuvants and linkers that were appropriate were added to the constructions. The stability and immunogenicity of every vaccine design demonstrated encouraging outcomes. As a result, the vaccine designs might be effective in preventing monkeypox, which would support further testing and similar strategies to combat other viral illnesses.

Introduction:

Since the 2022 outbreak, the total cases of Monkeypox worldwide have been increasing. As of August 2024, viral infections have infected around 99,000 people overall. Particularly, the World Health Organization (WHO) categorized this Mpox virus as a Public Health Emergency of International Concern (PHEIC) based on the clade I of the virus, which has raised the fatality rate (around 3.6%) [1]. Asian nations have recorded few cases, nevertheless, India, China, and Thailand have recorded some rare occurrences. India has recorded 30 cases since the epidemic started [2]. Though this virus was endemic to the Democratic Republic of Congo and central Africa, migration of the population has been a major cause of the spreading of the virus throughout the world [3]. MPXV is a double-stranded DNA (dsDNA) containing virus with an oval-shaped capsid which makes it in the family of Orthopoxvirus genus and Poxviridae family. The capsid structure has no clear symmetry like helical or icosahedral and the capsid is further developed with an outer lipid envelope [4]. Historical research shows that smallpox immunization provided around 85% protection against monkeypox. However, the eradication of smallpox and subsequent stopping of the orthopoxvirus vaccination effort for more than 40 years may have had a role in MPXV's reappearance [4], [5]. Another important reason for the return of monkeypox is its genetic evolution; for example, four separate lineages of the virus were discovered from region-specific samples in the Democratic Republic of the Congo. Recent research explored the development and epidemiology of MPXV in Nigeria and globally, citing gene loss/gain and recombination events in the advancement of MPXV [5]. There is currently no vaccination specifically for MPXV, despite reports of cross-protection through vaccinia-viral vaccines (ACAM2000 and JYNNEOSTM) [6]. When prescribed for severe MPXV infections, high-dose antiviral treatments (cidofovir, tecovirimat, and brincidofovir) sometimes cause hepatic dysfunctions, whereas low-dose therapies increase the chance of recurrence [6], [7], [8]. The primary limitation of the current MPXV vaccines is their ability to induce a persistent immune response and their specificity, particularly considering the pathogen's changing nature [8]. The current study developed a multi-epitope vaccine to stimulate human immunity against MPXV using the pan-genome and reverse vaccination technique. The process involves generating multiple epitopes from appropriate MPXV-proteins to elicit humoral and cellular immune responses without posing any health risks at the same time.

2.1 Sequence Retrieval and Pre-screening Phase

In our study, we systematically sourced and subsequently downloaded a total of 16 distinct whole genomic sequences, along with their corresponding proteome sequences, which collectively represent an impressive sample size of n = 16, all of which pertain to the MPXV and originate from 16 diverse geographical regions, sourced directly from the National Center for Biotechnology Information (NCBI) database, specifically to conduct a comprehensive pan-genomic analysis. To facilitate this intricate analysis, first, we reannotated the retrieved genomes with Prokka v1.14.6, and then two specialized bioinformatics software programs, namely ROARY (v3.13.0) and BPGA (version 1.3) were utilized for the pangenome analysis[9][10]. Roary was given input to the annotated genomes that were acquired via Prokka. Roary created a multi-FASTA alignment of core genes using PRANK v.170427 to create the gene presence/absence matrix [11]. Furthermore, in the BPGA analysis, we established a sequence identity criterion for the USEARCH algorithms, which was judiciously set at a threshold of 90%, a decision made to effectively cluster the genomic sequences in a manner that maximizes the reliability of our results [11]. Moreover, in addition to the aforementioned analyses, BPGA also facilitated the generation and accessibility of core, accessory, and distinct proteomic files, all of which are intricately linked to the genomic data, thus providing a holistic view of the genetic and proteomic landscape of the MPXV.

2.2 Subtractive Proteomics Filters

The fundamental core protein sequences of MPXV, obtained through comprehensive pan-genomic analysis, serve as the primary focus for the development of multi-epitope subunit vaccine models. Initially, we excluded all MPXV core proteins that exhibit homology to human proteins, retaining only those core proteins that lack any substantial similarities with human counterparts. The exclusion of MPXV proteins that closely resemble human proteins was implemented to mitigate the risks associated with autoimmunity or tolerance to foreign antigens. We employed the Non-redundant protein sequences database via blastP from NCBI to identify non-homologous proteins [12]. Subsequently, the non-homologous core proteins underwent evaluation for their subcellular localization. Proteins located within the cell membrane or extracellular regions are prioritized as

essential targets for potential vaccine candidates [13]. These membrane or extracellular proteins are accessible on the exterior of the viral cell and play a pivotal role in facilitating entry into the host cell through specific interactions. Herein, we employed the deepTMHMM server as a computational resource for the meticulous examination of the subcellular localization of various proteins [14]. Furthermore, we conducted a thorough analysis of the transmembrane helices associated with those proteins that had been systematically filtered through the TMHMM 2.0 server, thereby ensuring the integrity of our findings [15]. Transmembrane helices proteins are predominantly situated within the lipid bilayer of cellular membranes and play a multitude of critical roles, encompassing essential processes such as cell signaling, transport mechanisms, and overall cellular communication [16]. It is noteworthy that transmembrane helices, which are significantly shielded by the surrounding membrane structure, exhibit reduced accessibility to antibodies, consequently resulting in a diminished immunogenic response when exposed to the immune system. Subsequently, we undertook an evaluation of the antigenicity and allergenicity of the aforementioned filtered proteins utilizing the VaxiJen v2.0 and AllerTOP v.2.0 servers, during which proteins classified as non-antigenic and allergenic were systematically excluded from further consideration [17], [18]. Lastly, we assessed the physicochemical properties of these selected proteins through the ProtParam tool available on the ExPASy server, where we specifically focused our analysis on critical parameters including molecular weight, instability index, aliphatic index, and grand average hydrophobicity, thus providing a comprehensive characterization of these biomolecules [19].

2.3 Immune Cell Epitope Prediction

2.3.1 B Cell Epitope Prediction

One particular antigen area that interacts highly with B cell lymphocytes is the B cell epitope [20]. B cells might then go on to develop memory cells and antibodies that are specific to that antigen [21]. Hydrophilicity, accessibility, and the β turn region are the three factors that split the B cell epitope [22]. The immune epitope database (IEDB) website (http://tools.iedb.org/main/bcell/) presented us with the following approaches, which we utilized to predict the B cell epitope:

BepiPred, using baseline 0.5 thresholds. Finally, the Vaxijen v.2.0, AllerTOP v.2, and toxinpred servers exclude low antigenic, allergic, and toxic peptide sequences [23], [24].

2.3.2 MHC Class I and II Epitope Prediction

The Immune Epitope Database (IEDB) web server (http://tools.iedb.org/main/bcell/) was used to predict MHC class I epitopes. Peptides in the top 1% with a percentile rank less than 0.5 were chosen [25]. The organism was set to human, and the specified alleles were HLA-A01:01, HLA-A02:01, HLA-A03:01, HLA-A11:01, and HLA-B35:01. The IEDB web server was also utilized to predict MHC class II epitopes, with a percentile rank of less than 0.5 chosen. DRB101:01, DRB107:01, DRB103:01, DRB104:01, DRB108:01, DRB111:01, DRB115:01, and DRB1*13:01 were the DRB alleles that were defined. Lastly, the Vaxijen v2.0, AllerTOP v.2, and ToxinPred servers were used to assess the antigenicity, allergenicity, and toxicity of the peptide sequences to exclude peptides with low antigenicity, allergic, and toxic levels characteristics [26].

2.4 Multi-epitope Vaccine Construction

The Cytotoxin and helper T cell epitopes are connected to the unique GPGPG glycine-proline linker sequence, whereas the B cell epitopes are related to the Kk linker sequence[27]. The Large Ribosomal Subunit Protein bL12 has made contact with the N-termini of the epitopes, and the EAAAK stiff linker has connected this adjuvant to the epitopes [28]. The physicochemical properties of the vaccine, such as its molecular weight (MW), theoretical pI, atomic and amino acid composition, extinction coefficient, estimated half-life, instability index, aliphatic index, and grand average of hydropathicity (GRAVY), were computationally examined using Protparam's Expasy tool [29]. Furthermore, the developed multi-epitope vaccination was subjected to additional assessments of its antigenicity, allergenicity, and toxicity using the servers VaxiJen, AllerTOP v.2.0, and ToxinPred respectively [30].

2.5 Secondary Structure and Tertiary Structure Prediction

The sophisticated tools known as PSIPRED and Alphafold 2 were expertly employed in our study to design and elucidate the vaccine's secondary and tertiary structural configurations under investigation, respectively [31], [32]. PSIPRED server is an advanced protein structure investigation technique that utilizes the PSI-blast algorithm to analyze sequences. Through this, we were able to generate a comprehensive two-dimensional model that accurately predicts the secondary structure of a protein with remarkable precision based on high-quality primary sequence data. Using AlphaFold2, an advanced AI tool for protein structure prediction, we constructed a three-dimensional model of the protein, which enhanced our capacity for deep learning and pattern recognition. The model's local confidence was assessed using the predicted local distance difference test (pLDDT), a per-residue score scaled from 0 to 100, where higher values indicate higher confidence. A pLDDT above 90 reflects high accuracy in both backbone and side chains, while scores above 70 suggest reliable backbone prediction but potential side chain misplacement, providing insight into the reliability of different protein regions.

2.6 Structure Refinement and Validation

We further engaged the GalaxyRefine server to enhance and refine the three-dimensional structure that we had previously established using Alpha fold 2, thereby seeking to improve the quality and accuracy of our structural models. Galaxy Refine is a powerful web server that employs advanced techniques for protein refinement and structure prediction. By precisely refining key geometric features of the model, we optimized its structural configuration, ensuring proper folding and enhanced stability [33]. To assess the quality of the vaccine model, we employed the ProSA-web server which is adept at analyzing and displaying scoring metrics for high-quality protein models [34]. This server also calculates the energy of the protein structure and aids in identifying potential

flaws or deficiencies within the model, thereby providing critical insights into its overall quality and reliability. In addition to this, we checked the Ramachandran Plot to validate the accuracy of the protein structure through the SAVES v.6.0 web application which allowed us to rigorously examine whether our residues were located within the favored regions of the plot, thereby confirming the structural integrity of our model [35]. By analyzing the allowed and disallowed regions corresponding to the phi and psi angles, this method significantly enhances our comprehension of protein structure and is fundamentally essential for both the modeling of proteins and the design of effective vaccines, thus ensuring that the resulting models align with established structural biology principles.

2.7 Disulfide Engineering and Codon Optimization

To enhance both the stability and the expression efficacy of the vaccine, we employed methodologies encompassing disulfide engineering and codon optimization. For the disulfide engineering, we utilized the Disulfide by Design 2 (DbD2) computational server [36]. This tool systematically facilitates the introduction of disulfide bonds, thereby enhancing the stability of the vaccine without compromising the integrity of the overall antigenic determinants. More specifically, it identifies the cysteine residues present within the vaccine and strategically introduces disulfide bonds as necessary to augment the overall structural integrity of the vaccine. Codon optimization was conducted utilizing the VectorBuilder server, specifically focusing on the adaptation of the vaccine constructs for the E. coli K-12 strain [37]. We assessed the Codon Adaptation Index (CAI) value alongside the overall GC content of the engineered vaccine. Codon optimization is imperative for ensuring the high-level expression of vaccine proteins, achieved by aligning the DNA sequence with the codon usage preferences characteristic of the E. coli expression system. The VectorBuilder played a pivotal role by optimizing the sequences associated with the vaccine DNA to facilitate maximal translation efficiency. Subsequently, the modified sequences were synthesized and integrated into expression vectors specifically designed for E. coli K-12, a strain renowned for its robust protein expression capabilities.

2.8 Molecular Docking of the Protein with TLR-4

TLR4 plays a role in the innate immune response, particularly in the activation of pro-inflammatory cytokines during viral infections, and has been shown to identify pathogen-associated molecular patterns (PAMPs). TLR4 is an excellent target for the development of vaccine candidates since previous studies have demonstrated its crucial role in regulating immune responses to a variety of viral diseases. This receptor was selected because of its vital function in initiating immunological signaling pathways that lead to protective immunity, even though other receptors may also be involved in viral immune responses [38]. To determine TLR-4 docking with the vaccine protein, the Cluspro server was utilized. The Protein Data Bank supplied the TLR-4 structural coordinates. To visualize the docked structures and assess the interaction between TLR-4 and the vaccine protein, UCSF Chimera and Ligplot software were utilized [39].

2.9 Molecular Dynamic Simulation

The Schrödinger suite's Desmond v3.6 was used to do molecular dynamics (MD) simulations. The OPLS3e force field was used to describe atomic interactions and replicate how the vaccine models would behave in a biological setting. To create a hydrated environment that was comparable to physiological settings, each vaccine model was solvated in an orthorhombic periodic border box that was filled with TIP3P water molecules. The system was neutralized by adding the proper counter ions (Na+ or Cl-) to balance the total charge. Using the steepest descent approach, which minimizes potential energy by removing high-energy configurations and steric conflicts, the system was made to start from a stable configuration [40].

To enable the system to adapt to the simulated conditions, two equilibration phases were carried out following minimization. Initially, the Nose-Hoover thermostat was used to apply an NVT ensemble (constant Number, Volume, and Temperature) for 50 PS to keep the temperature at 300 K. During this stage, the system's volume remained constant while the temperature stabilized. The Martyna-Tobias-Klein barostat was then used to equilibrate the system for 50 PS utilizing NPT (constant Number, Pressure, and Temperature) [41]. This allowed the system's volume to fluctuate as necessary to obtain equilibrium density while stabilizing the system's pressure at 1 bar.

A 50 ns manufacturing run with a 2 femtosecond (fs) time step was carried out after equilibration to record atomic movements and interactions inside the system. Atomic coordinates were captured at intervals of 0.05 ns during this phase, giving a thorough temporal resolution of the dynamics of the vaccine models. To assess the vaccination models' flexibility and stability, two important structural studies were carried out [42]. The divergence of the protein's backbone and overall structure from its initial configuration was tracked using the Root Mean Square divergence (RMSD), which provides a measure of global stability. The flexibility of individual residues over time was evaluated using Root Mean Square Fluctuation (RMSF), which highlighted vaccination areas most susceptible to structural alterations or fluctuations [43].

Time-dependent changes in structure and interactions were visualized by post-simulation analysis using the Simulation Interaction Diagram (SID). Plotting the RMSD and RMSF data allowed for a better comprehension of the vaccination models' dynamic behavior under physiological settings by revealing information about their structural stability. This thorough MD simulation method ensured that the vaccine candidates preserved structural integrity in a physiologically relevant environment by providing vital information on their stability, flexibility, and general behavior [44], [45].

2.10 Immune Simulation Analysis

The capacity to swiftly predict and evaluate immune reactions to vaccine candidates renders in silico immune simulations essential for the advancement of vaccines. These simulations facilitate the acceleration of the development process and streamline the identification of viable vaccines for further scrutiny. This is accomplished through the identification of optimal antigen components, dosages, and formulations [10]. The formulated vaccine was uploaded to the C-IMMSIM server in fasta format to evaluate the anticipated immune response post-vaccination [46]. The outcomes of the analysis were then retrieved for detailed review. In this specific instance, a minimum interval of 28 days between doses was mandated. Three simulated injections were administered at time intervals of 1, 84, and 168, with each time step corresponding to 8 hours in reality. The default settings of the remaining server parameters were preserved.

Result

3.1 Pre-Screening Phase

To conduct a comprehensive pan-genome analysis that would facilitate a meticulous evaluation of the genetic diversity present across all 16 strains of the retrieved MPXV, we employed both the Roary and BPGA software tools, thereby enabling a robust cross-comparison of the core genes identified within these genomes. Before the implementation of Roary for the analysis, we meticulously annotated the genomes using Prokka, a widely recognized tool for genomic annotation that enhances the understanding of gene functions and structures. Upon executing Roary, our analysis revealed the existence of 170 core genes that are conserved across the assessed strains (**Supplementary file 1**). In comparison, a subsequent analysis utilizing BPGA indicated a slightly higher count of 180 protein-encoding core genes, thus highlighting a noteworthy discrepancy between the two methodologies employed (**Supplementary file 2**). Beyond the mere provision of essential statistical data regarding the counts of core, accessory, and unique genes, BPGA further enriched our analysis by generating a plethora of informative distributions, which included, but were not limited to, the Clusters of Orthologous Groups (COG) analysis, histogram representations of gene distributions, as well as the Kyoto Encyclopedia of Genes and Genomes (KEGG) distribution analyses, alongside default visualizations of core pan plots, as depicted in **figure 1A**, **1B**, and **1C**. Additionally, BPGA contributed to our understanding of phylogenetic relationships by generating both core and pan phylogenetic trees, which are visually represented in **Figures 2A** and **2B**, thereby offering insights into the evolutionary dynamics of the MPXV strains. Given the significance of the core proteins identified, we strategically targeted these proteins for further analytical endeavors to develop a multi-epitope subunit vaccine specifically designed to combat the MPXV, thereby addressing a critical gap in current preventive strategies against this viral pathogen.

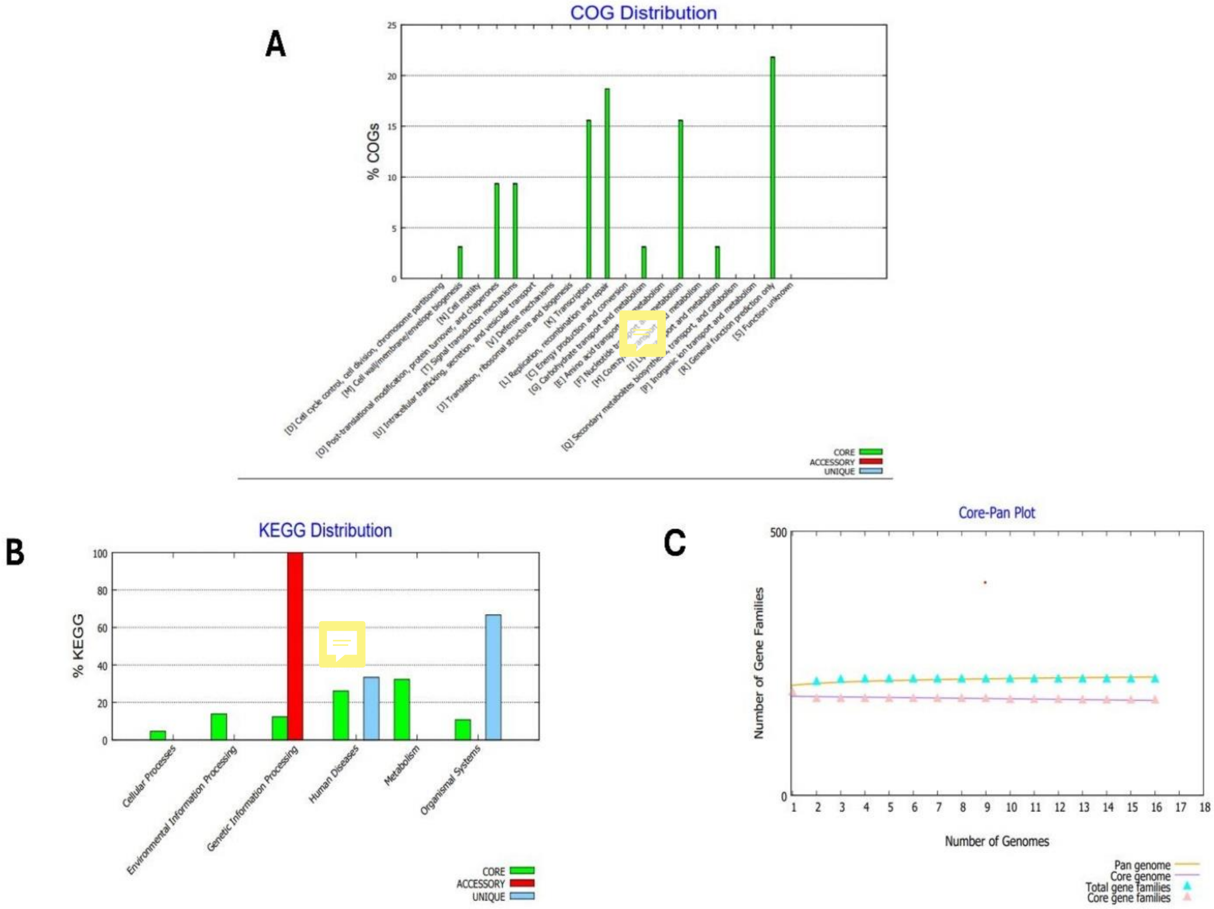

Figure 1: Visualization of the distributed proteomic data profiling of Monkeypox virus. Herein, (**A**) shows COG distribution, (**B**) shows KEGG distribution, and (**C**) shows the Core-pan plot of pan-genomic analysis.

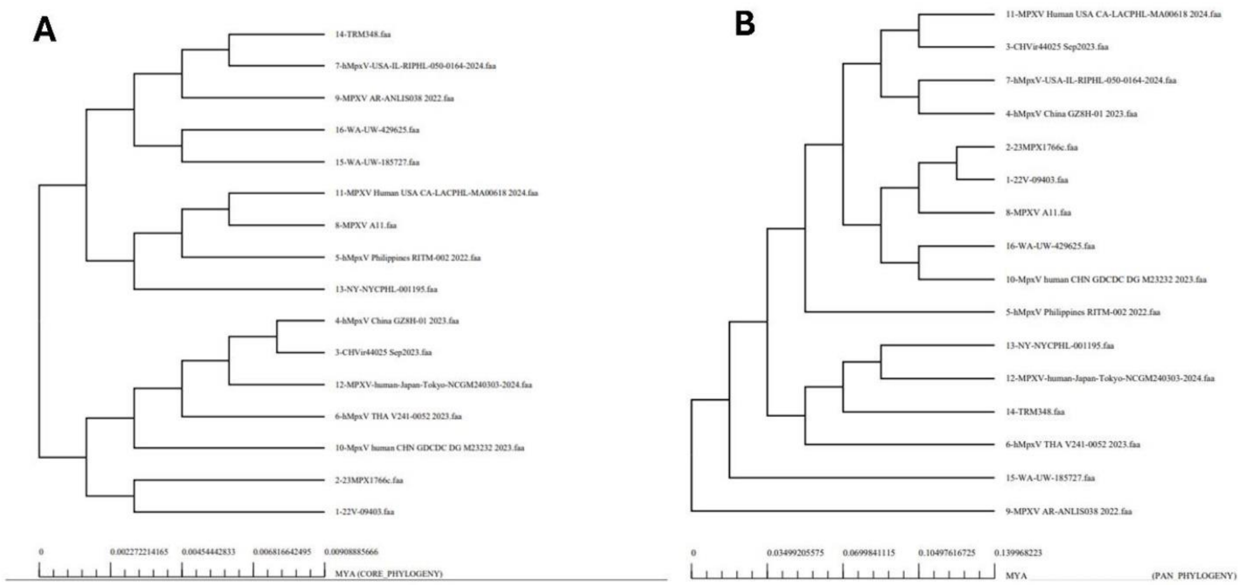

Figure 2: Visualization of (**A**) Core Proteins phylogenetic tree and (**B**) Phylogenetic tree of all the expressed proteins of Monkeypox virus.

3.2 Screening and Selection of Potential Vaccine Candidates

Among the 180 core proteins identified in our study, a total of 135 proteins were determined to be non-homologous to those found in the human genome, and critically, of these non-homologous proteins, a significant subset of 30 proteins was identified as residing within the membrane of the viral cell, indicating their potential role in viral pathology (**supplementary 3**). Upon conducting a thorough examination of the antigenicity associated with the remaining proteins, we were able to isolate 16 specific proteins that exhibited antigenic properties, suggesting their relevance in immune response mechanisms (**supplementary 4**). In addition to this, we proceeded to analyze the transmembrane helices of the remaining proteins, systematically discarding those proteins that scored above a threshold of 1, ultimately determining that all 16 proteins retained scores of either 0 or 1, thereby qualifying them for further investigation (**supplementary 4**). Moreover, we undertook a comprehensive assessment of the physiochemical properties inherent to these proteins, whereby we excluded any proteins that exceeded a molecular weight of 110 kD, along with those deemed unstable and hydrophobic. Our analyses also encompassed an evaluation of the aliphatic

index, leading us to retain only those proteins that demonstrated elevated scores in this particular metric, which serves as an indicator of protein stability and functionality. Following our thorough physiochemical properties analysis, we successfully identified a total of 6 proteins that present as ideal candidates for targeting in the strategic design of a multi-epitope vaccine aimed at combating the pathogenic effects of MPXV, thereby contributing to the advancement of vaccine development efforts against this viral threat (**supplementary 4**).

3.3 Epitope Prediction and Prioritization Phase

For epitope prediction, we utilized IEDB tools to assess the six initially selected proteins, evaluating their potential for B cell epitopes, MHC I, and MHC II binding. Only four of six proteins demonstrated sufficient immunogenicity and binding affinity during this phase, and these were taken for further analysis. All the six proteins are namely CORE_REP Org2 Gene185# (**Soluble interferon-gamma receptor-like protein)**, CORE_REP Org4_Gene19# (**hypothetical protein)**, CORE_REP Org11_Gene13# (**Interleukin-18-binding protein)**, CORE_REP Org6 Gene199# (**Virulence protein)**, CORE_REP Org10 Gene113# (**Late transcription factor VLTF-2)**, and CORE_REP Org2 Gene150# (**Envelope protein A28 homolog)**. Among them Org11_Gene13# and CORE_REP Org4_Gene19# were discarded due to less immunogenic epitopes and the rest of the four proteins were utilized for further progressions. In **CORE_REP Org2_Gene185#,** BepiPred predicted 11 B cell epitopes at a threshold of 0.5, leading to the selection of 5 epitopes with high antigenicity scores. MHC I analysis identified peptides ranking in the top 1% relevant to HLA-A01:01, HLA-A02:01, HLA-A03:01, HLA-A11:01, and HLA-B35:01, culminating in 20 prioritized candidates. MHC II predictions revealed 11 epitopes demonstrating strong binding affinity to DRB1 alleles, particularly DRB101:01 and DRB107:01 **(CORE_REP Org2_Gene185#)** (supplementary 5). In CORE_REP Org6_Gene199#, the analysis yielded 7 predicted B cell epitopes, with 4 selected based on antigenicity. MHC I analysis highlighted 13 epitopes exhibiting robust binding profiles, while MHC II predictions pointed to 13 epitopes with significant binding potential to DRB1 alleles **(CORE_REPOrg6_Gene199#)** (**supplementary 5**). Regarding CORE_REP Org10_Gene113#, 4 B cell epitopes were prioritized, while MHC I predictions identified 100 potential epitopes with high affinity to critical HLA alleles. MHC II predictions underscored 27 epitopes targeting DRB1 for optimal helper T cell activation

(CORE_REPOrg10_Gene113#) (**supplementary 5**). Lastly, in Org2_Gene150#, analysis prioritized 4 high-scoring B cell epitopes and identified 6 MHC I epitopes known for their strong HLA binding affinity. MHC II analysis selected 13 epitopes with high binding affinity to DRB1 alleles **(Org2_Gene150#)** (**supplementary 5**). This comprehensive approach ensured the selection of epitopes with optimal immunogenic and binding properties derived from each protein.

3.4 Multi-epitope Vaccine Construction

Antigenicity, allergenicity, and toxicity evaluations were conducted on prioritized B cell, MHC I, and MHC II epitopes using Vaxijen v2.0, AllerTop v.2.0, and ToxinPred servers. Non-allergenic, non-toxic epitopes with high antigenic potential were selected for vaccine design. Vaccine model 1 included epitopes from CORE_REP Org2_Gene185# and consisted of two B cells and three MHC I and MHC II epitopes (**Table 1**). T cell epitopes were linked using a GPGPG linker to enhance immune response, while B cell epitopes were connected using a KK linker. The large ribosomal subunit protein bL12 was an adjuvant, attached to the epitope construct with an EAAAK stiff linker. This configuration resulted in a construct of 255 amino acids. In Vaccine Model 2, epitopes collected from CORE_REP Org6_Gene199#, followed similar antigenic selection criteria and linker configurations, which brought the total amino acid count to 261, as shown in, **Table 2**. Model 3 utilized epitopes from CORE_REP Org10_Gene113# and included two B cell epitopes, one MHC I epitope, and three MHC II epitopes, totaling 257 amino acids (**Table 3**). Model 4 incorporated epitopes from Org2_Gene150# with the same linkage and adjuvant approach, resulting in a construct size of 293 amino acids, as represented in **Table 4**.

Table 1: MHC class I, class II, and B cell epitope predictions for Vaccine Model 1. The table includes Peptide sequences and their toxicity, allergenicity, and antigenicity.

Epitopes (CORE_REP Org2_Gene185#)	Peptides	Antigenicity	Allergenicity	Toxicity
B cell	GDYKVEEYCTG	0.9854	Non-Allergen	Non-Toxin
	LKYNYHFSDSSQITN	1.0409	Non-Allergen	Non-Toxin
MHC I	KIEWTGDGLY	1.2014	Non-Allergen	Non-Toxin
	SIHAKITSY	1.2853	Non-Allergen	Non-Toxin
	RSKEDVPNFK	0.6471	Non-Allergen	Non-Toxin
MHC II	KYNYHFSDSSQITNN	0.8948	Non-Allergen	Non-Toxin
	LKYNYHFSDSSQITN	1.0409	Non-Allergen	Non-Toxin
	YNYHFSDSSQITNNT	0.8236	Non-Allergen	Non-Toxin

Table 2: Predicted peptide sequences for MHC class I, class II, and B cell epitopes in Vaccine Model 2. The potential for antigenicity, allergenicity, and toxicity of each sequence were assessed.

Epitopes (CORE_REP Org6_Gene199#)	Peptides	Antigenicity	Allergenicity	Toxicity
B cell	DQKDYTVTSQFNNYTIG	1.0008	Non-Allergen	Non-Toxin
	PDGLDIPLT	0.6951	Non-Allergen	Non-Toxin
MHC I	VTMTYKNTK	1.7573	Non-Allergen	Non-Toxin
	SILGVSIECK	1.8785	Non-Allergen	Non-Toxin
	IPLTNITYW	0.9224	Non-Allergen	Non-Toxin
MHC II	ETDYTYLSNGGLP	0.8729	Non-Allergen	Non-Toxin
	TGETDYTYLSNGGLPA	0.8689	Non-Allergen	Non-Toxin
	GETDYTYLSNGGLP	0.8986	Non-Allergen	Non-Toxin

Table 3: B cell, MHC class I, and MHC class II epitopes were thoroughly analyzed for Vaccine Model 3. The antigenicity of the peptides is rated, and evaluations of their toxicity and allergenicity are included to guarantee vaccine development's safety and effectiveness.

Epitopes (CORE_REP Org10_Gene113)	Peptides	Antigenicity	Allergenicity	Toxicity
B cell	TIALKHSGYSSELNDISIGLTPNDTIKE	1.233	Non-Allergen	Non-Toxin
	FKNKTDII	0.7983	Non-Allergen	Non-Toxin
MHC I	ELNDISIGL	2.5692	Non-Allergen	Non-Toxin
	TPFDVEDTF	1.4167	Non-Allergen	Non-Toxin
	LSFIGYMVK	1.1948	Non-Allergen	Non-Toxin
MHC II	FGYVPYVGYKCINH	1.3178	Non-Allergen	Non-Toxin
	GYVPYVGYKCINH	1.0597	Non-Allergen	Non-Toxin
	PYVGYKCINHVS	1.2610	Non-Allergen	Non-Toxin

Table 4: Peptide sequence predictions for Vaccine Model 4, displaying class I, class II, and B cell epitopes. To evaluate the immunogenic potential and safety of the vaccine candidate and strive for a strong and non-allergic immune response, assessments of antigenicity, allergenicity, and toxicity are given.

Epitopes (Org2_Gene150#)	Peptides	Antigenicity	Allergenicity	Toxicity
B cell	YGNIKEFNATHAAFEYSKSIGGTPALDRRVQDVNDTISDVKQK	0.8069	Non-Allergen	Non-Toxin
	AEVGPNNTRSIRKFNTMRQC	0.1312	Non-Allergen	Non-Toxin
	SIFGFQAEV	0.4227	Non-Allergen	Non-Toxin

MHC I	ATHAAFEYK	0.8034	Non-Allergen	Non-Toxin
	FTFSDVINI	1.0007	Non-Allergen	Non-Toxin
MHC II	LSIFFIVVATAAVCLLFI	0.7240	Non-Allergen	Non-Toxin
	SIFFIVVATAAVCLLFIQ	0.7517	Non-Allergen	Non-Toxin
	AVCLLFIQSYSI	0.8065	Non-Allergen	Non-Toxin

3.5 Secondary Structure and Tertiary Structure Prediction

The secondary structure of the candidate vaccine was predicted utilizing the advanced computational tool known as PSIPRED, which effectively identified several pivotal structural components integral to the molecular framework, including alpha-helices, beta-strands, and random coil regions. The alpha-helices and beta-strands are known to form stable regions that contribute to the overall rigidity and stability of the protein structure, whereas the coil regions serve a critical role as flexible linkers that facilitate the necessary conformational dynamics within the molecule (**Table 5**). The high confidence scores obtained during this predictive analysis significantly bolstered the credibility of these structural predictions, thereby establishing a solid and reliable foundation for subsequent analyses and investigations into the vaccine candidate's properties. In the pursuit of elucidating the tertiary structure of the vaccine candidate, the cutting-edge software AlphaFold 2 was employed, which generated five distinct models of each vaccine that were subsequently ranked based on their respective confidence scores, specifically pLDDT values. We selected one model from each vaccine that exhibited high reliability, as evidenced by scores that consistently exceeded the pLDDT values threshold of 90. The 3D structure of those four designed vaccines was visualized using Chimera software and illustrated in (**Figure 4A-4D**).

Table 5: Percentage of Alpha helix, Beta sheet, and Random coil of designed vaccine models

	**Alpha helix**	**B-sheet**	**Random coil**
Vaccine Model-1	33.33%	14.51%	52.16%
Vaccine Model-2	28.35%	19.16%	52.49%
Vaccine Model-3	36.58%	15.18%	48.25%
Vaccine Model-4	58.36%	9.56%	32.08%

3.6 Population Coverage of Predicted T-Cell Epitope

In the context of natural biological systems, Major Histocompatibility Complex (MHC) molecules are found to be abundant and widely distributed across various communities and among multiple ethnic groups, highlighting their importance in the immune response. Consequently, the production of peptide-based vaccines is considered to be a highly effective strategy for the development of broad-spectrum vaccine candidates, particularly given the extensive array of MHC alleles present in diverse populations. The T cell epitopes identified in this comprehensive study revealed an average population coverage of 32.55% for MHC Class I, 18.23% for MHC Class II, and an impressive 44.85% when considering the combined coverage for the vaccine Model 1. In addition, the average population coverage for the vaccine Model 2 was calculated to be 22.63% for MHC Class I, 11.53% for MHC Class II, and 31.56% for combined coverage, thus indicating variability in the immune recognition patterns. Moreover, the vaccine Model 3 vaccine exhibited a remarkable average population coverage of 55.17% for MHC Class I, a considerably lower 3.96% for MHC Class II, and a combined coverage of 56.95%, underscoring the potential effectiveness of this vaccine model. Additionally, the vaccine Model 4 demonstrated an average population coverage of 51.06% for MHC Class I and a more substantial 28.79% for MHC Class II, culminating in an overall combined coverage of 65.15%, which suggests a robust ability to elicit an immune response across diverse populations. **Figure 3** represents a comprehensive illustration of the known class I and class II HLA alleles alongside the percentage of epitope group coverage, thereby providing a

visual representation of the findings and facilitating a deeper understanding of the potential efficacy of the vaccine candidates developed in this study.

Vaccine model-1

Population Coverage Calculation Result

population/area	Class I			Class II			Class combined		
	coverage[a]	average_hit[b]	pc90[c]	coverage[a]	average_hit[b]	pc90[c]	coverage[a]	average_hit[b]	pc90[c]
World	32.55%	0.51	0.15	18.23%	0.55	0.37	44.85%	1.06	0.18
Average	32.55	0.51	0.15	18.23	0.55	0.37	44.85	1.06	0.18
Standard deviation	0.0	0.0	0.0	0.0	0.0	0.0	0.0	0.0	0.0

Vaccine model-2

Population Coverage Calculation Result

Note: Following allele(s) are not available and therefore not included in the calculation, please note that allele names are case sensitive. HLA-DRB1*01:13,

population/area	Class I			Class II			Class combined		
	coverage[a]	average_hit[b]	pc90[c]	coverage[a]	average_hit[b]	pc90[c]	coverage[a]	average_hit[b]	pc90[c]
World	22.63%	0.39	0.13	11.53%	0.23	0.23	31.56%	0.63	0.15
Average	22.63	0.39	0.13	11.53	0.23	0.23	31.66	0.63	0.16
Standard deviation	0.0	0.0	0.0	0.0	0.0	0.0	0.0	0.0	0.0

Vaccine model-3

Population Coverage Calculation Result

population/area	Class I			Class II			Class combined		
	coverage[a]	average_hit[b]	pc90[c]	coverage[a]	average_hit[b]	pc90[c]	coverage[a]	average_hit[b]	pc90[c]
World	55.17%	0.63	0.22	3.96%	0.12	0.31	56.95%	0.75	0.23
Average	55.17	0.63	0.22	3.96	0.12	0.31	56.95	0.75	0.23
Standard deviation	0.0	0.0	0.0	0.0	0.0	0.0	0.0	0.0	0.0

Vaccine model-4

Population Coverage Calculation Result

population/area	Class I			Class II			Class combined		
	coverage[a]	average_hit[b]	pc90[c]	coverage[a]	average_hit[b]	pc90[c]	coverage[a]	average_hit[b]	pc90[c]
World	51.06%	0.94	0.2	28.79%	0.41	0.14	65.15%	1.35	0.29
Average	51.06	0.94	0.2	28.79	0.41	0.14	65.15	1.35	0.29
Standard deviation	0.0	0.0	0.0	0.0	0.0	0.0	0.0	0.0	0.0

Figure 3: Population coverage results of all the designed vaccines.

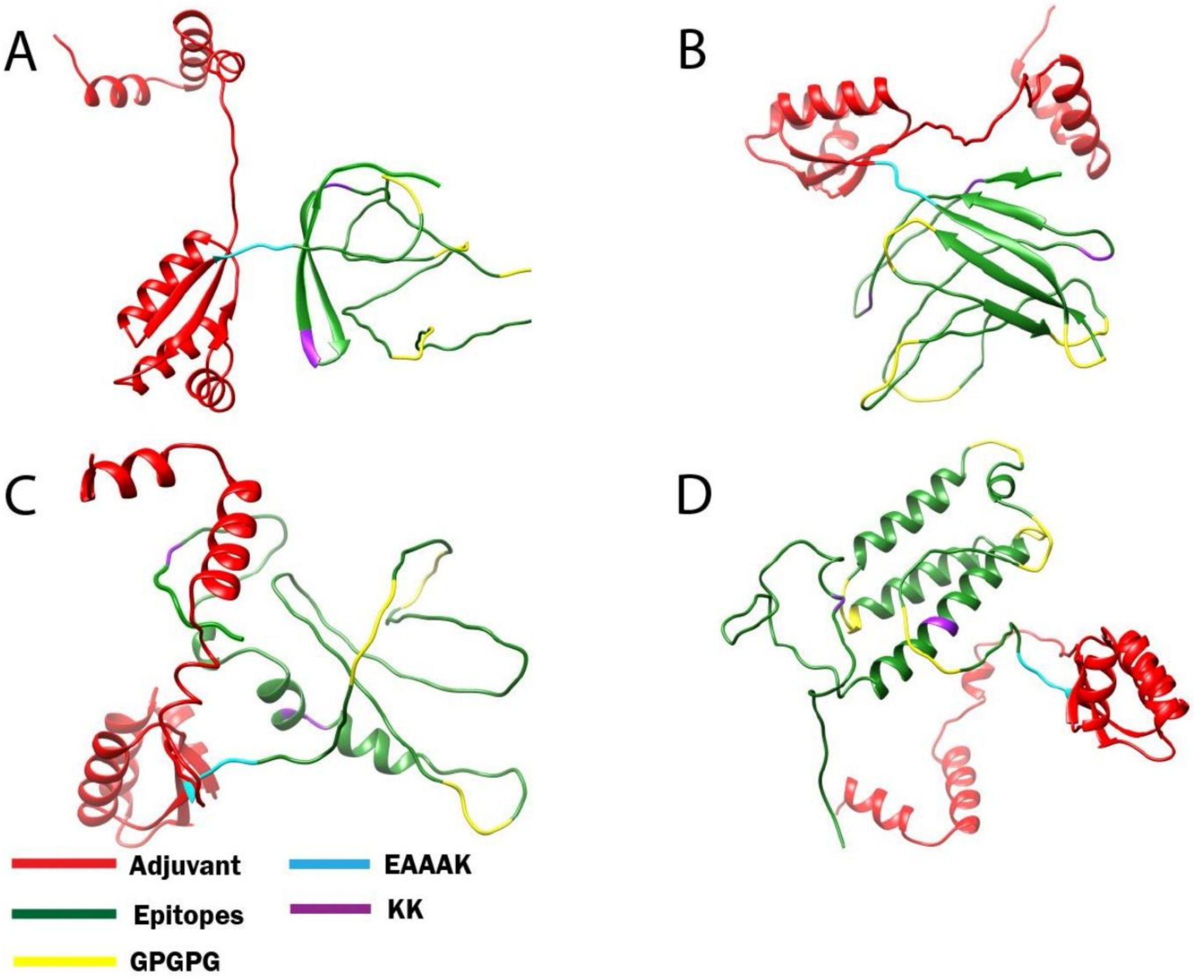

Figure 4: A representation of the vaccine structures for Vaccine Models 1-4. Models 1 to 4 are represented as (**A**), (**B**), (**C**), and (**D**). Important details are emphasized in various colors: Adjuvant areas appear in red, anticipated epitopes in forest green, GPGPG linker sequences in yellow, EAAAK linkers in cyan, and KK linkers in purple. The structural arrangement and composition of every vaccine model are illustrated by these color-coded properties

3.7 Structure Refinement and Validation

To enhance and refine the accuracy of the predicted three-dimensional model, the Galaxy Refine server was utilized, which is known for its ability to improve the precision of structural models by optimizing their geometric characteristics. The refinement process was particularly focused on enhancing the overall quality of the model, placing a significant emphasis on achieving proper folding and stabilization of the tertiary structure to ensure that it accurately reflects the natural

conformations of proteins. Following this refinement, the model exhibited a marked improvement in accuracy, especially in regions that had initially been characterized by a lower degree of structural definition or stability. The refined three-dimensional model underwent a rigorous validation process using the ProSA-web tool, which provides z-scores indicative of the structural quality of the protein; the scores obtained were -5.98 for Vaccine model 1, -6.76 for Vaccine model 2, -4.4 for Vaccine model 3, and -3.84 for Vaccine model 4 each reflecting the overall quality and reliability of the protein structure (**Figure 5A**-**5D**). The z-scores achieved indicated that the refined structural model resided comfortably within the realm of typical high-quality protein models, thereby signifying that the predicted model was structurally sound and reliable for further investigations. Furthermore, the ProSA-web analysis revealed no significant energy anomalies within the structure, which serves as an additional confirmation of its stability and reliability. Lastly, the structural validation process was concluded utilizing the Ramachandran plot through the SAVES v.6.0 server, where more than 90% of the residues were found to reside within the favored regions of the plot in all the vaccine models, thus affirming the correctness of the phi and psi dihedral angles that are critical for proper protein folding (**Figure 6A**-**6D**). This validation process conclusively confirmed that the overall geometry of the model adhered to established structural norms and standards.

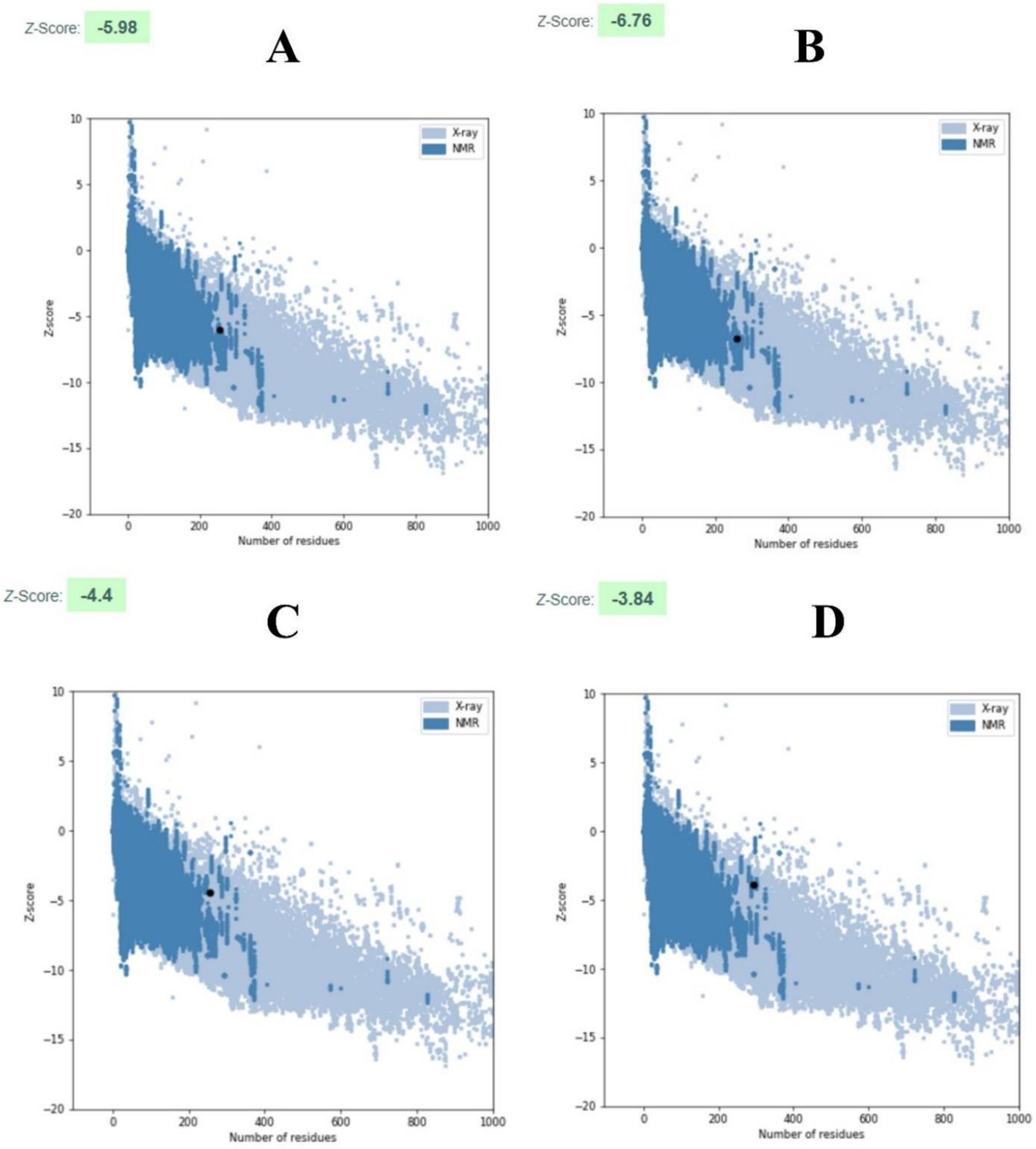

Figure 5: The generated result of all the Z scores for each vaccine model where (**A**) represents Vaccine Model 1, (**B**) represents Vaccine Model 2, (**C**) represents Vaccine Model 3, and, (**D**) represents Vaccine Model 4.

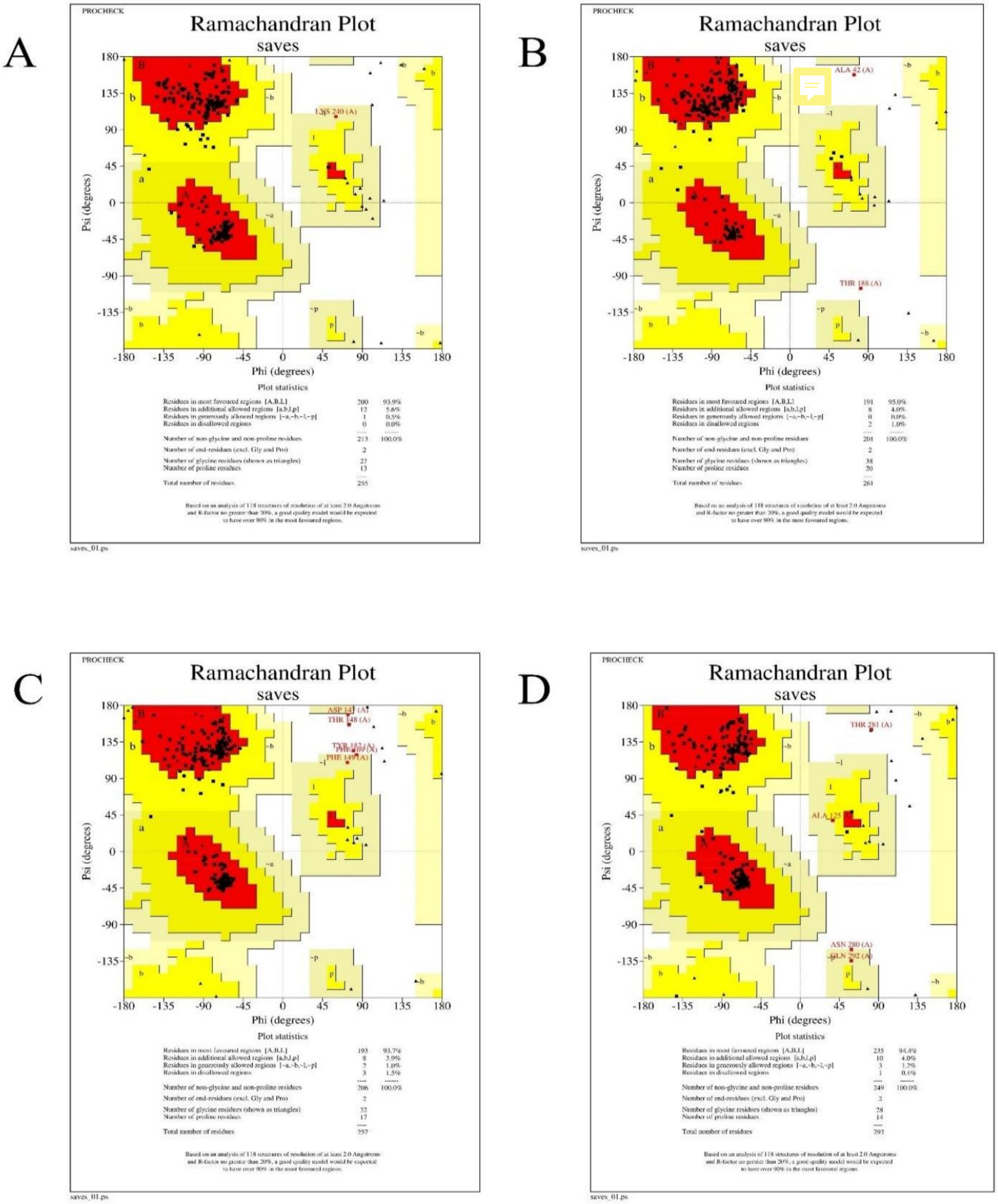

Figure 6: Graphical illustration of Ramachandran plot of all the designed vaccine models. (**A**) represents Vaccine Model 1, (**B**) represents Vaccine Model 2, (**C**) represents Vaccine Model 3, and, (**D**) represents Vaccine Model 4

3.8 Disulphide Engineering and In-silico Codon Optimization

The vaccine underwent disulfide engineering, which was meticulously designed to enhance and fortify the intermolecular bonding characteristics of the vaccine, thereby significantly improving the overall structural stability of the vaccine formulation. This intricate process was critical in ensuring that the more fragile segments of the vaccine were rendered more resilient against potential cellular degradation, consequently imparting a higher degree of conformational stability to the vaccine's architecture. Throughout the rigorous analytical assessment, only those residue pairs that exhibited a higher energy value, specifically those exceeding 0 kcal/mol, were selected for the mutation to cysteine, ensuring that only the most energetically favorable modifications were made (**Supplementary file 6**). The specific amino acid residues that underwent replacement by cysteine have been systematically tabulated for clarity and ease of understanding, while red sticks visually represent the resultant cysteine bonds formed as a result of this strategic alteration in the accompanying diagrams (**Figure 7**). This meticulous approach not only elucidates the importance of disulfide bonds in maintaining vaccine integrity but also underscores the intricate relationship between molecular structure and functional efficacy in vaccine design.

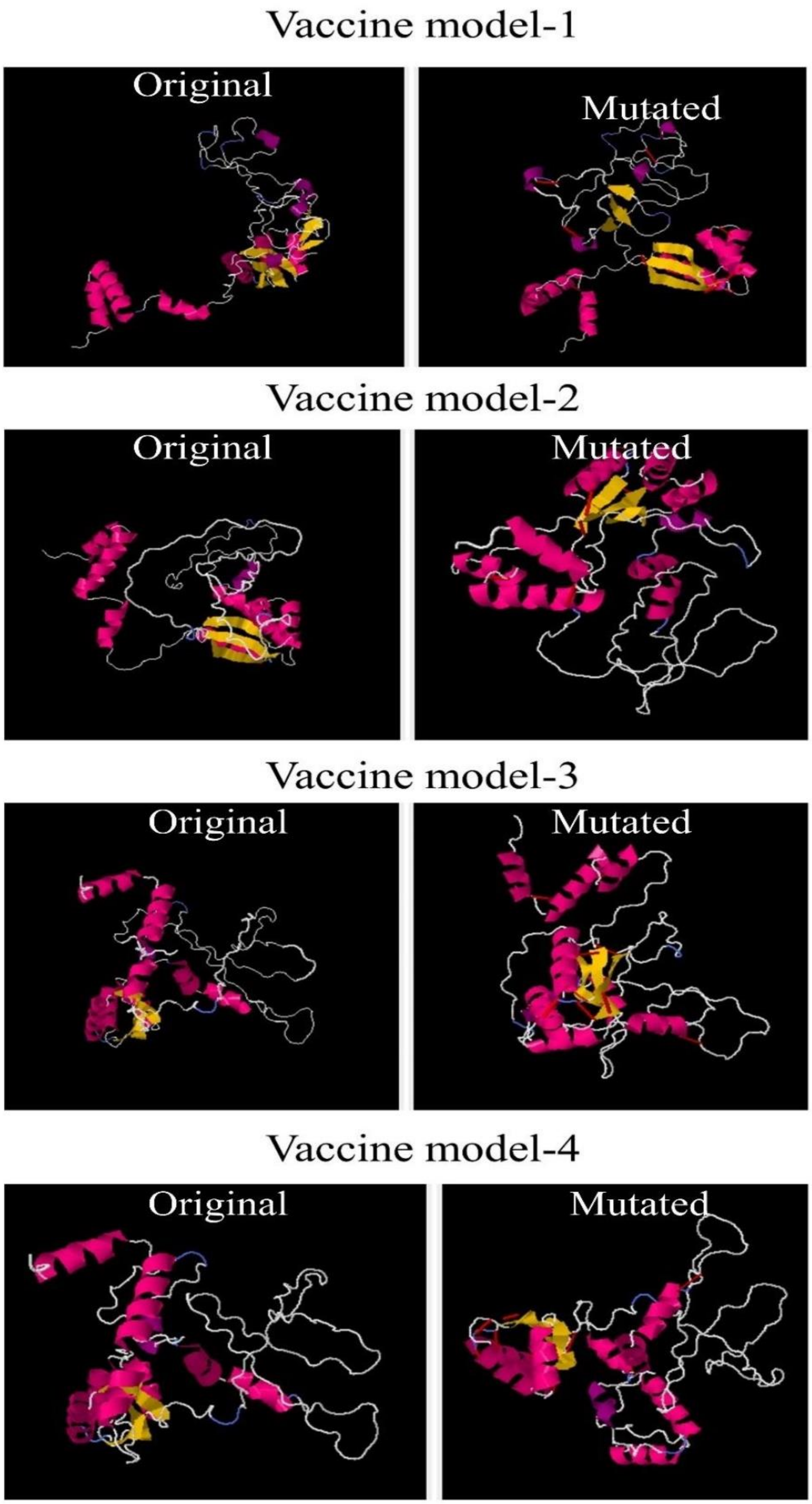

Figure 7: All the Left Panels show the designed vaccine models and all the right panels show a mutated version of the vaccine where red colored sticks represent newly added disulfide bonds.

3.9 Molecular Docking Results of Vaccine Models with TLR-4

The vaccine proteins' interaction with TLR-4 was assessed using the Cluspro service to determine the possible immune response stimulation. Protein-protein docking simulations yielded 29 clusters for each vaccine model-TLR4 complex. The top-ranked docked clusters were chosen based on their cluster size and lowest energy scores, suggesting a better binding affinity and interaction stability between the vaccine models and TLR-4. According to the docking simulation, vaccine model 1 has a center energy and a lowest energy of -799 kcal/mol, which suggests moderate stability and potential for interaction with TLR-4 (**Figure 8A**, **Table 6**). With a center energy of -946.2 Kcal/mol and a lowest energy of -1123.3 Kcal/mol, vaccine model 2 appeared to have a higher binding affinity for TLR-4 than vaccine model 1(**Figure 8B**, **Table 7**). The docking findings for vaccine model 3 showed that its center energy was -1025.6 Kcal/mol, and its lowest energy was -1030.9 Kcal/mol. This indicates that vaccine model 3 has the highest binding affinity to TLR-4 and is the most stable interaction among the models examined (**Figure 8C**, **Table 8**). In comparison to vaccine model 3, vaccine model 4 showed a somewhat less robust center energy of -958.4 kcal/mol, with the lowest energy below -1068 kcal/mol, indicating a steady interaction (**Figure 8D**, **Table 9**).

Table 6: The interaction of hydrophilic and hydrophobic bonds between TLR4 receptor and Vaccine model 1.

**Types**	**TLR4(Chain B)**	**Vaccine model 1**
Hydrophilic	Thr514, His458, Gln484, Arg460, Lys541, Ser482, rg606, Arg382, Thr359, Tyr403, Asp428, Asp405	Lys101, Ser34, Ala42, Asp102, Ala35, Ser16, Ile3, Met1, Ser2, Lys5
Hydrophobic	Pro513, Ser512, Gln505, Leu553, His431, His456, Ala462, Phe538, Glu484, Asp550, Ser552, Gln507, Asn575, Asn481, Gly480, Glu603, Phe408, Gln578, Ser360, Val338, Asp379, Thr357, His426	Glu51, Ile24, Val21, Met27, Vl33, Gly32, Ala38, Val46, Ala43, Gly44, Val39, Val39, Vl17, Phe31, Met18

Table 7: The interaction of hydrophilic and hydrophobic bonds between TLR4 receptor and Vaccine model 2.

**Types**	**TLR4(Chain C)**	**Vaccine model 2**

Hydrophilic	Gln156, Gln41, Ser45, Ser27, Asn26, Lys58, Trp23, Lys20, Ser28, Tyr34	Thr188, Ser141, Tyr163, Thr191, Pro205, Tyr193, Thr162, Asn160, Glu190, Asn178
Hydrophobic	Met40, Lys39, Leu60, Gly59, Phe64, His62, Asn49, Pro50, Cys25, Val24, Pro43, Tyr22	Pro186, Pro139, Gly138, Pro137, Ala203, Asp192, Tyr175, Pro166, Gly167, Pro202, Leu201, Gly204, Gly179, Gly183, Leu181, Gly180, Hel142, Gly165, Gly189, Trp164

Table 8: The interaction of hydrophilic and hydrophobic bonds between TLR4 receptor and Vaccine model 3.

**Types**	**TLR4(Chain C)**	**Vaccine model 3**
Hydrophilic	Asn49, Asn47, Ser45, Val24, Trp23, Lys20	Pro167, Gly168, Phe169, Tyr171, Lys163, Tyr160, Asp147, Glu146
Hydrophobic	Asn114, Ser103, Thr115, Phe64, Ile44, Tyr22, Gln19, Lys39, Gln41, Met40	Gly203, Pro204, Tyr207, Pro173, Gly170, Met161, Gly164, Pro165, Thr148, Gly152, Val145, Gly150, Pro153

Table 9: The interaction of hydrophilic and hydrophobic bonds between TLR4 receptor and Vaccine model 4.

**Types**	**TLR4(Chain B)**	**Vaccine model 4.**
Hydrophilic	Lys20, Gln21, Glu144, Pro142, Lys91, Glu136, Lys89	Lys5, Asp6, Arg257, Thr251, Gly250, Gly249
Hydrophobic	Glu143, Tyr75, Lys39, Met145, Ile138, Asn77, Tyr79	Ser247, Asp255, Ala253, Ile248

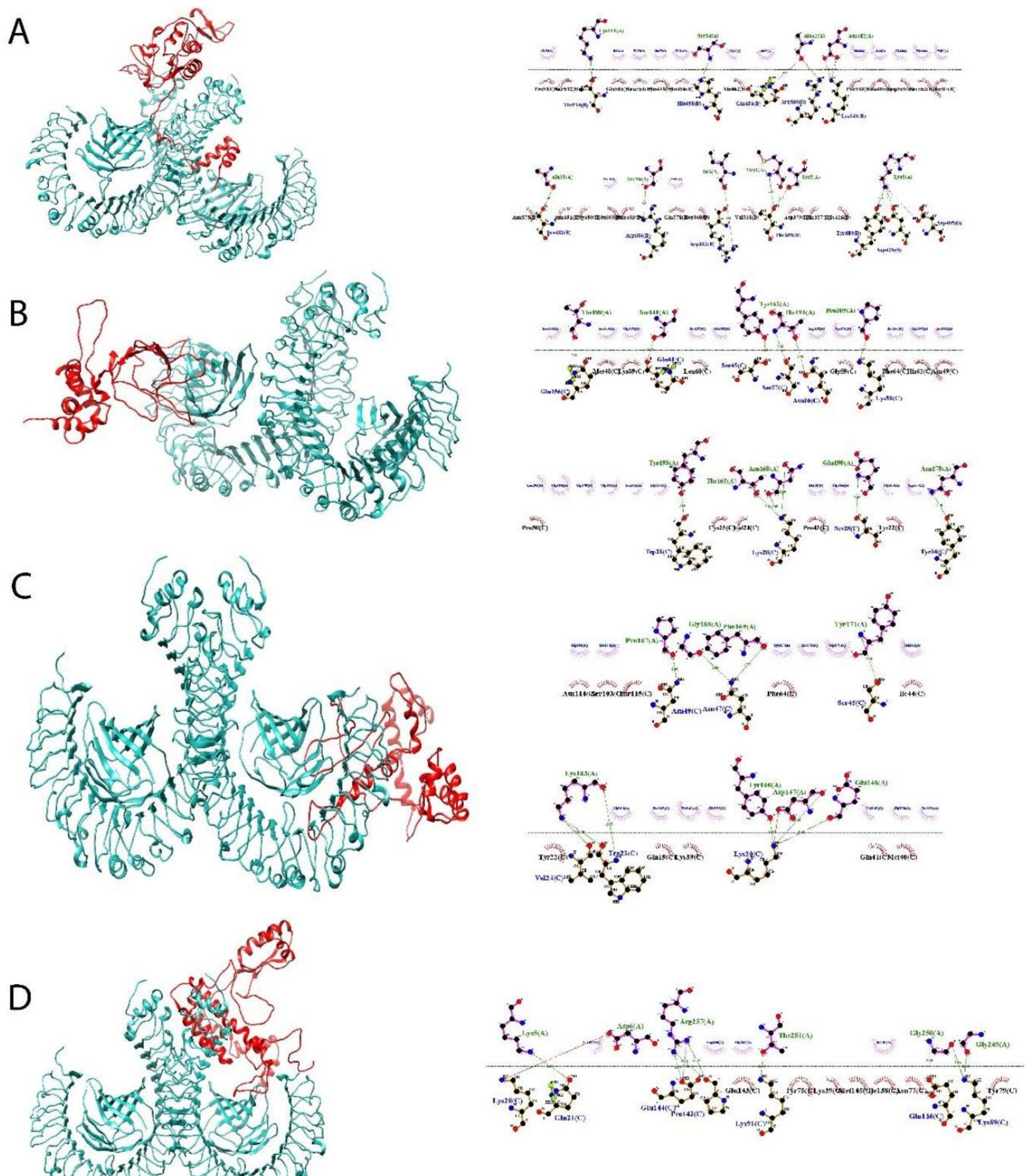

Figure 8: Vaccine-TLR4 receptor complex models for Vaccine Models 1–4. Models from 1 to 4 are represented by letters A to D. The TLR4 receptor is depicted in light sea green in each combination, whereas the vaccine is displayed in red. The spatial conformation of the complex is depicted in the left panels, which show the 3D structure of the vaccine-receptor binding interaction. A simplified version of the vaccine-TLR4 binding interface is presented in the right panels, which provide a 2D model of the interaction

3.10 Molecular Dynamic Simulation

A potent computational method for examining the dynamic behavior and structural integrity of atoms and molecules at the atomic scale is molecular dynamics simulations or MDS. When used in vaccine design, MD simulations offer vital information on the stability and binding affinity of a vaccine candidate as well as how it interacts with its target protein. The intensity and longevity of these interactions over time can be assessed by mimicking the vaccine's binding to the active site of a protein. MDS makes it possible to thoroughly examine how well a vaccine binds to its target protein. The complex's overall conformational stability and flexibility are measured using important measures such as root mean square deviation (RMSD) and root mean square fluctuation (RMSF). To determine whether the binding is stable or prone to dissociation, RMSD shows how the protein-ligand complex's structure changes from its starting state throughout the simulation. Conversely, RMSF identifies parts of the protein that have a great deal of flexibility and may impact ligand binding. Furthermore, assessing secondary structure elements (SSEs), which are essential to the structural stability of proteins and include α-helices, β-sheets, and loops, is a crucial component of MDS. It is possible to evaluate how vaccine binding impacts the protein's secondary structure and whether notable changes occur by monitoring the SSE during simulations. Strong ligand binding is frequently indicated by stability in these secondary structures, while instability or weak connections may be indicated by disruptions or unfolding.

During the 50 ns molecular dynamics simulation, the Root Mean Square Deviation (RMSD) is an essential metric for assessing the vaccination models' structural integrity. An RMSD value between 1 and 5 Å is typically seen as a sign of high stability, indicating little structural variation from the original shape. Throughout the simulation, Vaccine Model 1 continuously kept its RMSD values between 1.5 Å and 2.5 Å. This narrow fluctuation range demonstrates the model's resilience and implies that it underwent few conformational changes, maintaining its structural integrity throughout time. Similar trends were seen in Vaccine Model 2, where RMSD values varied from 2 Å to 4 Å, occasionally surpassing the 3 Å threshold while being within stability-acceptable bounds. Although these variations point to minor variations, they are not substantial enough to imply structural instability. Vaccine Model 3 also displayed a steady RMSD profile, with values falling between 2 Å and 3.5 Å. This implies that during the simulation, Model 3, like Models 1 and 2, maintained its structural stability with little conformational changes. Model 3's RMSD

values are within a stable range, albeit being marginally greater than Model 1's, suggesting that it is similarly stable. Moreover, Vaccine Model 4 also showed significant RMSD variations, which is also acceptable (**Figure 9A-9D**).

During the simulation, the flexibility of each particular residue in the protein structure is measured by Root Mean Square Fluctuation (RMSF). Greater flexibility or instability is suggested by larger RMSF values, whereas more stiff parts are indicated by lower RMSF values (usually less than 1-2 Å). Vaccine Models 1 and 2 showed comparatively low changes across the majority of residues in the RMSF analysis for the vaccine models. The fact that most of the residues stayed below 2 Å suggests that these models had a rigid core structure with little room for flexibility. Small peaks surrounding residues in particular areas (maybe loop or surface-exposed regions) were more than 2 Å, but they did not exhibit significant variation and remained well within acceptable bounds for protein stability. These peaks most likely correspond to areas of flexible loops that preserve the models' overall structural integrity. A similar pattern was seen in Vaccine Model 3, where the majority of the residues had RMSF values less than 2 Å. The general pattern indicates that Model 3 likewise maintains a decent level of structural stability, with only a few flexible locations contributing to higher RMSF values, even though some places displayed slightly larger fluctuations than Models 1 and 2. Vaccine Model 4 showed greater oscillations in some areas, with RMSF values surpassing 5 Å and in some areas even surpassing 8 Å. Particularly in areas that are probably exposed to the surface or engaged in dynamic interactions, these notable peaks may imply localized flexibility and structural instability. The overall conformation of the protein may not become unstable as a result of this degree of variation. Proteins' total three-dimensional conformation depends on secondary structure elements (SSE), and their persistence over time is an indication of structural stability. With just minor alterations in the ratios of α-helices and β-sheets, Vaccine Model 1 continuously maintained more than 25% of its original secondary structure over the simulation. Similar to Model 1, Vaccine Model 2 retained more than 20% of its secondary structure, albeit with a few small variations, suggesting a minimal loss in structural integrity. Conversely, after 30 ns, Vaccine Model 3's SSE stability gradually decreased, indicating a noticeable loss of secondary structure. Due to its minor substantial RMSD and RMSF swings, Vaccine Model 4 showed a minor loss, with SSE retention falling below 10% early in the

experiment. These findings demonstrate that Models 1, Model 2, Models 3, and Model 4 all are structurally stable and can turn out as stable candidate (**Figure 10A-10D**).

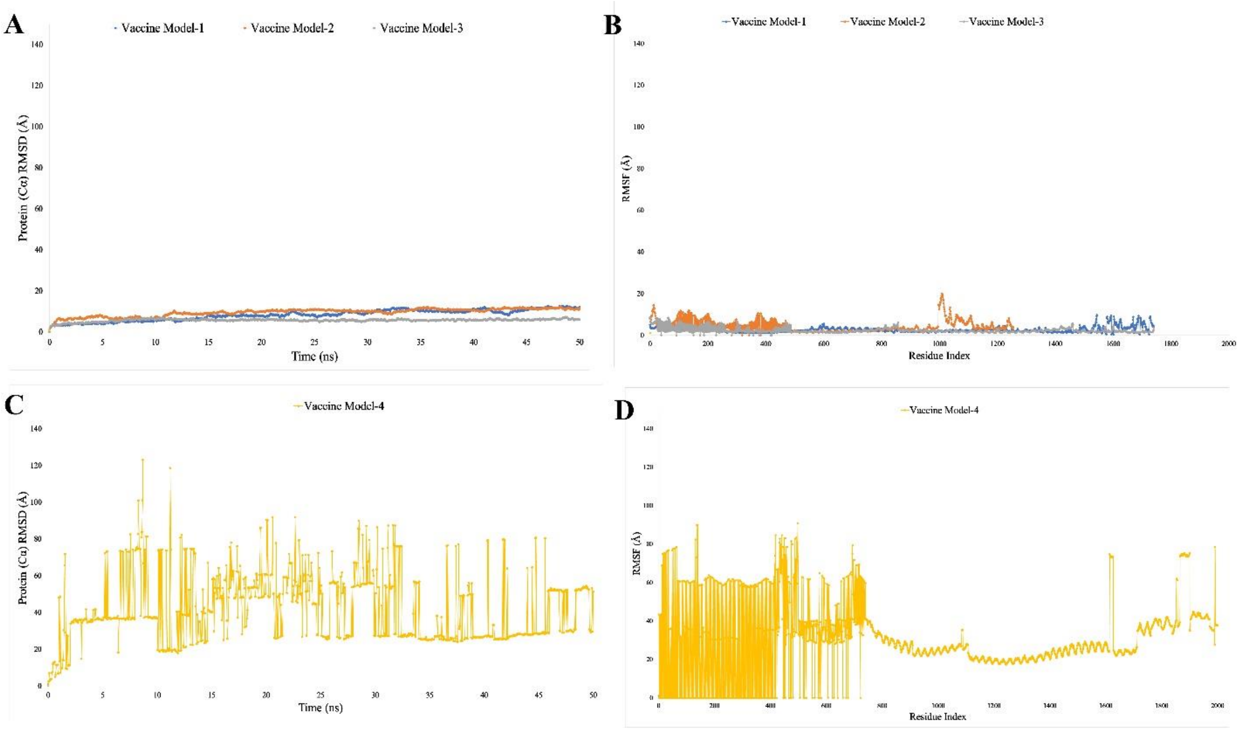

Figure 9: RMSD and RMSF of the vaccine model-1, Model-2, Model-3, and Model-4

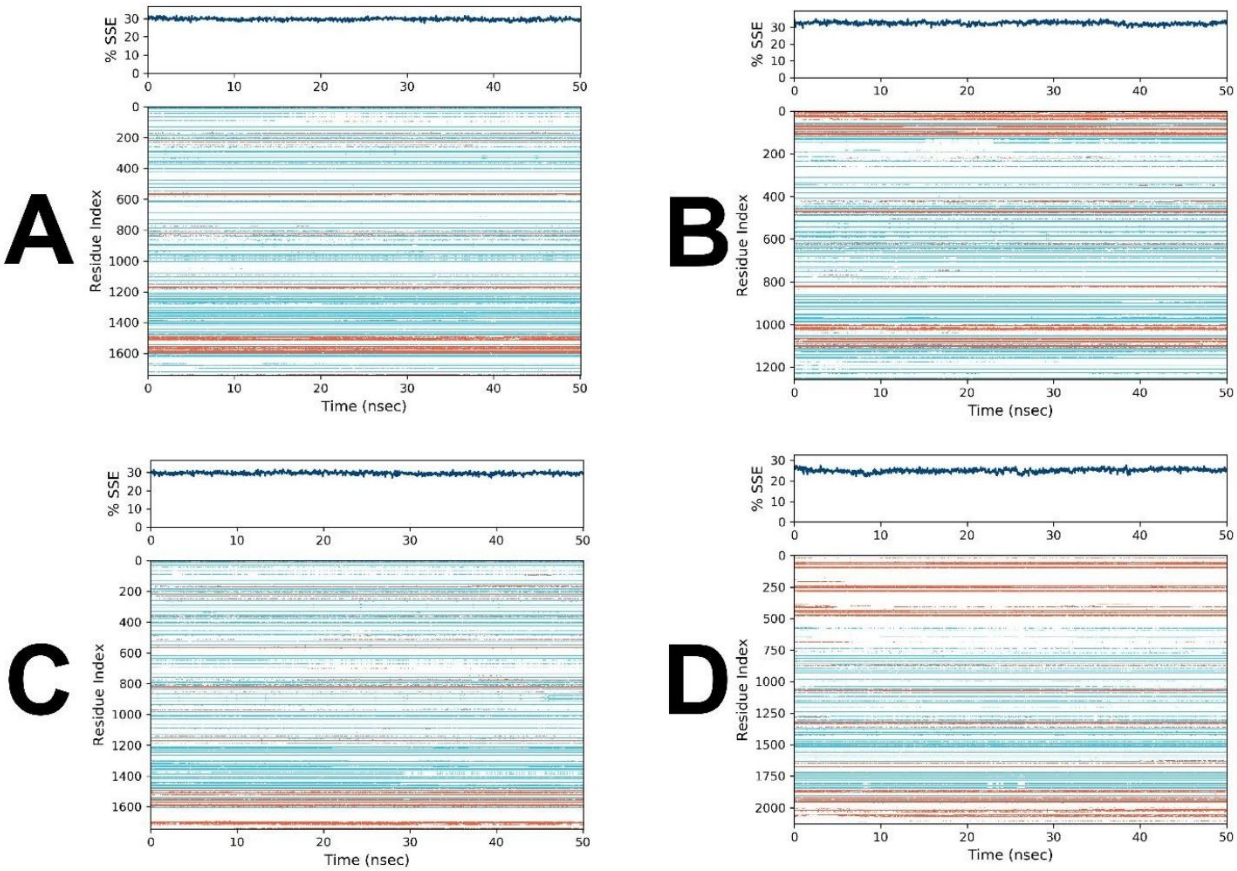

Figure 10: Secondary Structure Element analysis of **A)**. vaccine model-1, **B)**. vaccine Model-2, **C).** Vaccine Model-3, and **D).** Vaccine Model-4**.**

3.11 Immune Response Simulation

The experimental models of the immune response exhibited parallels to the genuine immunological occurrences triggered by specific pathogens, as illustrated in the **supplementary file 7**. Notably, the secondary and tertiary immune responses of all the selected vaccine model candidates were significantly more vigorous than the primary immune response. The secondary and tertiary responses were defined by elevated levels of various antibody types (including IgG1 + IgG2, IgM, and IgG + IgM), which coincided with a reduction in the antigen load, thereby indicating the formation of memory cells and the subsequent enhancement of antigen elimination during subsequent exposures. Furthermore, an increased lifespan was noted in B-cells, cytotoxic T-cells, and helper T-cells, suggesting a transition among immune cells and the establishment of IgM memory. It was also clear that IFN, IL-4, and IL-10 were all overexpressed. It's interesting to

note that compared to Th1-type immune reactions, Th0-type immune responses had lower percentages (%) and quantities (cells/mm3). Throughout the presentation, it was shown that although dendritic cell movement was determined to be predictable, macrophage mobility increased dramatically.

4. Discussion

The need for a suitable vaccination against the MPXV is urgent given the increasing number of children and immunocompromised individuals who die from it worldwide. Because of its changing pathogenic characteristics, the lack of effective vaccinations or treatment alternatives against MPXV has created serious risks. The most effective and economical way to stop virus outbreaks is by using vaccines. Inducing humoral and cell-mediated immunity against the target virally infected cells should be a feature of an effective vaccination [3]. Hence, a genome-based reverse vaccinology approach was adopted to design extensively validated multi-epitopic vaccine candidates that can efficiently provide immunity against MPXV infections.

Numerous studies have explored the use of immunoinformatics as a first-line vaccine design technique, contributing to the development of effective multi-epitopic MPXV vaccines. But in our study, we utilized recently published complete sequence data derived from different parts of the world since the 2022 outbreak. Pan-genome-based screening removes the constraints (biases) of data obtained from secondary/curated sources and confirms the global homogeneity of vaccine targets in MPXV. Potential target proteins were selected from annotated complete genomes of MPXV based on their solubility, antigenicity, outer-membrane position, and heterology to human proteins. While eliciting immunological responses, the conservation qualities guaranteed little cross-reactivity. Pathogen surface proteins, of course, have a higher chance of interacting with the host's immune system and triggering an immunological response [47]. At the in vitro and in vivo levels, the vaccinia virus membrane proteins have previously been assessed as a target of neutralizing antibodies and as a component of the subunit vaccine [48]. To anticipate putative T-cell and B-cell epitopes and create a multi-peptide-based vaccination that can elicit a robust CD4+ and CD8+ T-cell-related immunological response, recent immunoinformatics research targeted on the E8L, B6R, and SERP2 proteins of MPXV [49], [50]. However, other MPXV proteins that have an exposed position might potentially be viable options for vaccine development. To identify

immunodominant T-cell and B-cell epitopes and create a multi-epitope vaccine that can cross-protect against different strains of the virus causing the current outbreak, we examined all membrane-bound, enveloped, and extracellular proteins of MPXV and chose 4 proteins using a reverse vaccinology pipeline. Consequently, MPXV's outer-membrane proteins were refined to extract MHC class I T-cell epitopes, MHC class II T-cell epitopes, and B-cell epitopes, which, upon binding, might elicit potent immunological responses. Several server-based estimates confirmed the same. To improve efficacy, the final vaccine constructs were created by aligning many epitopes and joining them with adjuvants, GPGPG, and KK linkers. The vaccine may stimulate and activate a more robust protective immune response in recipients due to the adjuvants used in its formulation. All these constructs were screened for antigenicity, allergenicity, and toxicity in silico to validate their safety profile leading to developmental efficacy. The computational modeling and structural predictions of the vaccine candidates gave us valuable insights into their stability and immunogenicity. Using AlphaFold, the 3D structures of these vaccines were generated and Chimera was used to visualize them. The predicted stable and well-folded structures of the selected vaccine models implied that these models were necessary to guarantee sufficient immune recognition. Population coverage of the selected epitopes showed variation in immune response concerning different MHC alleles, and this variation indicates the need for a vaccine design that can induce broad immune responses within diversified populations. Vaccine Model 1, combining the highest coverage for MHC Class I and II alleles, in particular, holds promise for effective immune response in a wide range of individuals. Together with other models, this gave a full framework for developing a vaccine that could conceivably protect against MPXV in numerous populations around the world. Molecular dynamics simulations were performed to further confirm the structural stability of the vaccine candidates. Indeed, stability was maintained for Models 1 and 2 in simulation, indicating that these might potentially withstand physiological conditions. By contrast, larger conformational changes were observed with Vaccine Model 4. The molecular docking of the vaccine models with TLR-4 showed that the vaccine candidates have promising interactions with this important receptor involved in the initiation of innate immune responses, thus increasing the likelihood of stimulating a strong immune response. Finally, disulfide engineering and in-silico codon optimization of the vaccine constructs further enhanced the structural stability and expression efficiency of the latter. In this respect, disulfide bonds were used to stabilize the proteins, and hence functional integrity upon expression was

ensured. Codon optimization has been done to increase the expression of the vaccine in an appropriate host system, which may produce the vaccine in considerable amounts required for further preclinical and clinical trials.

Considering the developed vaccine contains core epitopes, which may provide broad-spectrum immune protection against a range of pathogen types, it has significant clinical promise. Its computational study also indicates that it has a good safety profile because it is non-toxic and non-allergic. However, to enhance its translational effectiveness, multiple limitations require to be addressed in further research. The most effective epitope combination inside the vaccine design that generates a potent and persistent immune response must initially be identified through experimental validation. Secondly, continuous improvements in MHC epitope prediction algorithms will enhance the selection of highly immunogenic epitopes by further improving antigen presentation accuracy. Finally, comprehensive in vitro and in vivo studies such as immunogenicity evaluations, animal challenge studies, and clinical trials, are required to validate the vaccine's long-term safety and protective effectiveness. It will be possible to develop a therapeutically feasible vaccine candidate with significant immunoprotective potential if these limitations are addressed.

5. Conclusions

The current study designs a multi-faceted approach for the development of a multi-epitope vaccine against MPXV. We combined the use of bioinformatics tools and experiments for the identification of potential vaccine candidates that could generate strong immune responses. Results obtained herein provide a basic contribution to the development of an effective vaccine against Monkeypox that could have a very crucial effect on this emergent and re-emergent global threat. Due to this fact, further testing of vaccine candidates in preclinical and clinical settings is imperative for confirmation of their safety and efficacy profile.

References

[1] "Mpox," WHO. [Online]. Available: https://www.who.int/news-room/questions-and-answers/item/mpox

[2] T. E. Times, "Monkey Pox becomes a global threat: is India at risk and what is the current status," 2024. [Online]. Available: https://economictimes.indiatimes.com/news/india/monkey-pox-becomes-a-global-threat-is-india-at-risk-and-what-is-the-current-status/articleshow/112643726.cms?from=mdr

[3] E. M. Bunge *et al.*, "The changing epidemiology of human monkeypox—A potential threat? A systematic review," *PLoS Negl. Trop. Dis.*, vol. 16, no. 2, p. e0010141, 2022.

[4] E. Petersen *et al.*, "Human monkeypox: epidemiologic and clinical characteristics, diagnosis, and prevention," *Infect. Dis. Clin.*, vol. 33, no. 4, pp. 1027–1043, 2019.

[5] E. Alakunle, U. Moens, G. Nchinda, and M. I. Okeke, "Monkeypox virus in Nigeria: infection biology, epidemiology, and evolution," *Viruses*, vol. 12, no. 11, p. 1257, 2020.

[6] J. G. Rizk, G. Lippi, B. M. Henry, D. N. Forthal, and Y. Rizk, "Prevention and treatment of monkeypox," *Drugs*, vol. 82, no. 9, pp. 957–963, 2022.

[7] C. Wattal and S. Datta, "Monkey pox arrives in India," 2022, *Elsevier*.

[8] H. Adler *et al.*, "NHS England High Consequence Infectious Diseases (Airborne) Network. Clinical features and management of human monkeypox: a retrospective observational study in the UK," *Lancet Infect Dis*, vol. 22, no. 8, pp. 1153–1162, 2022.

[9] A. J. Page *et al.*, "Roary: rapid large-scale prokaryote pan genome analysis," *Bioinformatics*, vol. 31, no. 22, pp. 3691–3693, 2015.

[10] M. H. J. Shovon, M. Imtiaz, P. Biswas, M. M. I. Tareq, M. N. H. Zilani, and M. N. Hasan, "A pan-genomic analysis based multi-epitope vaccine development by targeting Stenotrophomonas maltophilia using reverse vaccinology method: an in-silico approach," *Silico Pharmacol.*, vol. 12, no. 2, pp. 1–25, 2024.

[11] R. Edgar, "Usearch," Lawrence Berkeley National Lab.(LBNL), Berkeley, CA (United States), 2010.

[12] R. Lavigne, D. Seto, P. Mahadevan, H.-W. Ackermann, and A. M. Kropinski, "Unifying classical and molecular taxonomic classification: analysis of the Podoviridae using BLASTP-based tools," *Res. Microbiol.*, vol. 159, no. 5, pp. 406–414, 2008.

[13] K. Rawal *et al.*, "Identification of vaccine targets in pathogens and design of a vaccine using computational approaches," *Sci. Rep.*, vol. 11, no. 1, p. 17626, 2021.

[14] J. Hallgren *et al.*, "DeepTMHMM predicts alpha and beta transmembrane proteins using deep neural networks," *BioRxiv*, pp. 2004–2022, 2022.

[15] S. Möller, M. D. R. Croning, and R. Apweiler, "Evaluation of methods for the prediction of membrane spanning regions," *Bioinformatics*, vol. 17, no. 7, pp. 646–653, 2001.

[16] J. Dai *et al.*, "The Role of SF1 and SF2 Helicases in Biotechnological Applications," *Appl. Biochem. Biotechnol.*, pp. 1–21, 2024.

[17] I. A. Doytchinova and D. R. Flower, "VaxiJen: A server for prediction of protective antigens, tumour antigens and subunit vaccines," *BMC Bioinformatics*, vol. 8, pp. 1–7, 2007, doi: 10.1186/1471-2105-8-4.

[18] I. Dimitrov, I. Bangov, D. R. Flower, and I. Doytchinova, "AllerTOP v.2 - A server for in silico prediction of allergens," *J. Mol. Model.*, vol. 20, no. 6, 2014, doi: 10.1007/s00894-014-2278-5.

[19] E. Gasteiger, "Protein identification and analysis tools on the ExPASy server," *proteomics Protoc. handbook/Human Press Inc*, 2005.

[20] W. Hoffman, F. G. Lakkis, and G. Chalasani, "B Cells, Antibodies, and More," *Clin. J. Am. Soc. Nephrol.*, vol. 11, no. 1, 2016.

[21] J. A. James and J. B. Harley, "B-cell epitope spreading in autoimmunity," *Immunol. Rev.*, vol. 164, no. 1, pp. 185–200, 1998.

[22] M. C. Jespersen, S. Mahajan, B. Peters, M. Nielsen, and P. Marcatili, "Antibody specific B-cell epitope predictions: leveraging information from antibody-antigen protein complexes," *Front. Immunol.*, vol. 10, p. 298, 2019.

[23] A. Grifoni, J. Sidney, Y. Zhang, R. H. Scheuermann, B. Peters, and A. Sette, "A sequence

homology and bioinformatic approach can predict candidate targets for immune responses to SARS-CoV-2," *Cell Host Microbe*, vol. 27, no. 4, pp. 671–680, 2020.

[24] A. Grifoni, J. Sidney, Y. Zhang, R. H. Scheuermann, B. Peters, and A. Sette, "Candidate targets for immune responses to 2019-Novel Coronavirus (nCoV): sequence homology- and bioinformatic-based predictions," *Soc. Sci. Res. Netw.*, 2020.

[25] A. S. S. Girija *et al.*, "Prediction of Putative Epitope Peptides against BaeR Associated with TCS Adaptation in Acinetobacter baumannii Using an In Silico Approach," *Medicina (B. Aires).*, vol. 59, no. 2, p. 343, 2023.

[26] K. S. Lata, V. Vaghasia, S. Bhairappanvar, S. Patel, and J. Das, "Vaccine design against leptospirosis using an immunoinformatic approach," *Immunoinformatics*, pp. 173–184, 2020.

[27] H. A. Dar *et al.*, "Immunoinformatics-aided design and evaluation of a potential multi-epitope vaccine against Klebsiella pneumoniae," *Vaccines*, vol. 7, no. 3, p. 88, 2019.

[28] N. Ban *et al.*, "A new system for naming ribosomal proteins," *Curr. Opin. Struct. Biol.*, vol. 24, pp. 165–169, 2014.

[29] M. Naveed *et al.*, "Execution and design of an anti HPIV-1 vaccine with multiple epitopes triggering innate and adaptive immune responses: an immunoinformatic approach," *Vaccines*, vol. 10, no. 6, p. 869, 2022.

[30] M. M. Rahman *et al.*, "An immunoinformatics prediction of novel multi-epitope vaccines candidate against surface antigens of Nipah Virus," *Int. J. Pept. Res. Ther.*, vol. 28, no. 4, p. 123, 2022.

[31] J. Jumper *et al.*, "Highly accurate protein structure prediction with AlphaFold," *Nature*, vol. 596, no. 7873, pp. 583–589, 2021.

[32] L. J. McGuffin, K. Bryson, and D. T. Jones, "The PSIPRED protein structure prediction server," *Bioinformatics*, vol. 16, no. 4, pp. 404–405, 2000.

[33] L. Heo, H. Park, and C. Seok, "GalaxyRefine: Protein structure refinement driven by side-chain repacking," *Nucleic Acids Res.*, vol. 41, no. W1, pp. W384–W388, 2013.

[34] M. Wiederstein and M. J. Sippl, “ProSA-web: Interactive web service for the recognition of errors in three-dimensional structures of proteins,” *Nucleic Acids Res.*, vol. 35, no. SUPPL.2, pp. 407–410, 2007, doi: 10.1093/nar/gkm290.

[35] C. Colovos and T. O. Yeates, “Verification of protein structures: patterns of nonbonded atomic interactions,” *Protein Sci.*, vol. 2, no. 9, pp. 1511–1519, 1993.

[36] D. B. Craig and A. A. Dombkowski, “Disulfide by Design 2.0: A web-based tool for disulfide engineering in proteins,” *BMC Bioinformatics*, vol. 14, no. 1, pp. 0–6, 2013, doi: 10.1186/1471-2105-14-346.

[37] L. J. de Oliveira Andrade, G. C. M. de Oliveira, L. C. M. de Oliveira, and L. M. de Oliveira, “Homologies between Chloroplast, plasma membrane, plastid, chloroplast envelope, putative [Theobroma cacao] and PEG10 protein [Homo sapiens]: a starting point for to deliver drugs to cells?,” 2024.

[38] E. J. Mifsud, A. C. L. Tan, and D. C. Jackson, “TLR agonists as modulators of the innate immune response and their potential as agents against infectious disease,” *Front. Immunol.*, vol. 5, p. 79, 2014.

[39] M. O. Rafi, K. Al-Khafaji, M. T. Sarker, T. Taskin-Tok, A. S. Rana, and M. S. Rahman, “Design of a multi-epitope vaccine against SARS-CoV-2: immunoinformatic and computational methods,” *RSC Adv.*, vol. 12, no. 7, pp. 4288–4310, 2022.

[40] A. M. da Fonseca *et al.*, “Screening of potential inhibitors targeting the main protease structure of SARS-CoV-2 via molecular docking, and approach with molecular dynamics, RMSD, RMSF, H-bond, SASA and MMGBSA,” *Mol. Biotechnol.*, vol. 66, no. 8, pp. 1919–1933, 2024.

[41] S. Ghahremanian, M. M. Rashidi, K. Raeisi, and D. Toghraie, “Molecular dynamics simulation approach for discovering potential inhibitors against SARS-CoV-2: A structural review,” *J. Mol. Liq.*, vol. 354, p. 118901, 2022.

[42] D.-D. Li *et al.*, “Molecular dynamics analysis of binding sites of epidermal growth factor receptor kinase inhibitors,” *ACS omega*, vol. 5, no. 26, pp. 16307–16314, 2020.

[43] N. Michaud-Agrawal, E. J. Denning, T. B. Woolf, and O. Beckstein, “MDAnalysis: a

toolkit for the analysis of molecular dynamics simulations," *J. Comput. Chem.*, vol. 32, no. 10, pp. 2319–2327, 2011.

[44] A. Samad, N. S. Meghla, Z. Nain, T. M. Karpiński, and M. S. Rahman, "Immune epitopes identification and designing of a multi-epitope vaccine against bovine leukemia virus: a molecular dynamics and immune simulation approaches," *Cancer Immunol. Immunother.*, vol. 71, no. 10, pp. 2535–2548, 2022.

[45] C. J. Oomen *et al.*, "Immunogenicity of peptide-vaccine candidates predicted by molecular dynamics simulations," *J. Mol. Biol.*, vol. 328, no. 5, pp. 1083–1089, 2003.

[46] N. Rapin, O. Lund, M. Bernaschi, and F. Castiglione, "Computational immunology meets bioinformatics: the use of prediction tools for molecular binding in the simulation of the immune system," *PLoS One*, vol. 5, no. 4, p. e9862, 2010.

[47] R. Sanches *et al.*, "Immunoinformatics Design of Multi-Epitope Peptide-Based Vaccine Against Schistosoma mansoni Using Transmembrane Proteins as a Target," *Front. Immunol.*, vol. 12, p. 621706, Mar. 2021, doi: 10.3389/fimmu.2021.621706.

[48] J. E. Moyron-Quiroz, M. M. McCausland, R. Kageyama, A. Sette, and S. Crotty, "The smallpox vaccine induces an early neutralizing IgM response.," *Vaccine*, vol. 28, no. 1, pp. 140–147, Dec. 2009, doi: 10.1016/j.vaccine.2009.09.086.

[49] M. Yousaf, S. Ismail, A. Ullah, and S. Bibi, "Immuno-informatics profiling of monkeypox virus cell surface binding protein for designing a next generation multi-valent peptide-based vaccine.," *Front. Immunol.*, vol. 13, p. 1035924, 2022, doi: 10.3389/fimmu.2022.1035924.

[50] S. Aiman *et al.*, "Multi-epitope chimeric vaccine design against emerging Monkeypox virus via reverse vaccinology techniques- a bioinformatics and immunoinformatics approach.," *Front. Immunol.*, vol. 13, p. 985450, 2022, doi: 10.3389/fimmu.2022.985450.
