# Supplementary material for: Pan-genome and reverse vaccinology for a multi-epitope vaccine against circulating post-2022 Monkeypox virus strains
Source: PLoS One. 2026 Jun 22;21(6):e0351881. doi: 10.1371/journal.pone.0351881 (PMC13286271; doi:10.1371/journal.pone.0351881)
Supplement: Attachment — Submitted filename: Response to Reviewers.docx [file pone.0351881.s019.docx]

Reviewer #1:

Thank you for the opportunity to revise our manuscript. we appreciate the reviewers' thoughtful comments and suggestions, which have helped us improve the quality and clarity of our work. We have addressed each comment in the point-by-point response below and updated the manuscript accordingly.

1. When the authors are aiming to construct a vaccine, they should include more number of strains to avoid any biasness. Exactly how many strains were considered in this study?

Thank you for your feedback. The study included 16 different strains based on different locations retrieved from the NCBI database, which were downloaded unbiasedly.

2. What is the cytoplasmic location of the core protein which are non-homologous to human proteins?

The cytoplasmic location of the core proteins that are non-homologous to human proteins was given in the supplementary file 3.

3. Why does the authors considered only TLR-4 for docking with the different vaccine construct?

Specifically, TLR-4 is chosen because it recognizes lipopolysaccharides and other microbial components, making it a key trigger for innate immunity and an ideal target to predict vaccine-induced immune activation.

4. Why particularly OPLS3e force field has been used during MD simulation?

The OPLS3e force field is used during MD simulation because it provides highly accurate and optimized parameterization for both proteins and ligands, ensuring reliable modelling of biomolecular interactions and conformational stability.

5. 50ns MD simulation is not sufficient to prove the stability of the vaccine construct. The authors are advised to extend the simulation to at least 200ns and do it in triplicates.

We appreciate the reviewer’s insightful comment. The vaccine model and the receptor protein used in our study are large biomolecules composed of multiple interacting subunits, and therefore, extending the molecular dynamics simulation (MDS) to 200 ns would require substantial computational resources. Since the vaccine-receptor complex simulation in this study primarily aims to evaluate the potential immune response and overall stability of the construct, a 50 ns simulation was initially considered adequate.

6. The authors should also perform the MD simulation of the vaccine-target protein complex to check the stability of the complex.

Thank you for the suggestion; we appreciate the reviewer’s comment and would like to note that the MD simulation of the vaccine-target protein complex has already been performed to evaluate and confirm the stability of the complex

7. The authors should provide the figures for immune simulation result in main text.

We thank the reviewer for this valuable suggestion. The immune simulation result figures are currently included in the supplementary file; however, we will include them in the main text to improve the clarity and accessibility of the findings.

8. What are the next steps for experimental validation, and have any specific animal models or immunoassays been considered or recommended?

The next steps for experimental validation include obtaining funding to synthesize the vaccine construct, followed by in vitro immunoassays to assess immunogenicity, and subsequently conducting animal trials to evaluate immune response and safety in an appropriate model.

9. Authors are advised to rewrite the discussion and make the findings of your research clear to the reader.

We appreciate the reviewer’s suggestion and will revise the discussion to clearly highlight and interpret the key findings of our research for the reader.

10. Authors should improve the figure quality and provide a schematic representation of the vaccine construct.

We thank the reviewer for the suggestion and will improve the figure quality in our revised manuscript.

11. The discussion section is not satisfactory. The authors should use other studies that have been done in this field in this section and compare their results with the results of those studies. It is suggested that you use the following studies in this section:

<https://doi.org/10.1016/j.biologicals.2024.101782>
<https://doi.org/10.1007/s13205-024-04022-6>
<https://doi.org/10.3390/v14112504>
<https://doi.org/10.1007/s12026-023-09403-2>
<https://doi.org/10.1016/j.vacun.2024.04.003>

We appreciate the reviewer’s suggestion to incorporate relevant studies into our discussion. We have reviewed the following articles and will cite those findings into our revised discussion.

12. The manuscript has many typographical and grammatical errors. Please improve the manuscript by someone who is a native English speaker.

We thank the reviewer for this comment and will have the manuscript carefully proofread to correct all typographical and grammatical errors.

**Reviewer #2:**

1. Novelty and Literature Context:

Several studies have employed immunoinformatics approaches for developing multi-epitope vaccines against monkeypox virus, such as the recent publication [refer DOI: 10.3390/v14112374]. The authors should clearly articulate the novelty and unique contributions of this work relative to prior research. This includes specifying innovative aspects of epitope selection, vaccine construct design, adjuvant/linker usage, molecular docking strategies, or molecular dynamics parameters used.

**Answer:**

Most existing studies, including Kar et al. (2022), were conducted using either a single reference genome (NC_063383.1) or early outbreak sequences from mid-2022. In contrast, our study utilizes 16 distinct whole genomic sequences sourced from diverse geographical regions, specifically including isolates from the ongoing post-2022 outbreak up to August 2024. This ensures that our vaccine targets are based on the most current evolutionary trajectory of the virus. Unlike standard reverse vaccinology that relies on a single proteome, we employed a dual-tool pan-genome approach using Roary and BPGA to identify 80-170 core genes conserved across 100% of the selected strains. This methodology "removes the constraints and biases" of secondary/curated data and ensures the global homogeneity of our vaccine targets, making the resulting vaccine potentially effective against all circulating MPXV clades.

2. Language and Grammar:

The manuscript requires thorough proofreading and correction of spelling and grammatical errors. For instance, the sentence “Our goal was to Computationally creat and evaluate…” should be revised to “Our goal was to computationally create and evaluate…”. Ensure consistent use of capitalization and verb forms throughout the text.

**Answer:**

We thank the reviewer for this comment and will have the manuscript carefully proofread to correct all typographical and grammatical errors.

3. Consistency in Terminology:

The receptor name should be uniformly presented as either “TLR4” or “TLR-4” throughout the manuscript.

**Answer:**

We have standardized the terminology and used **“TLR4”** consistently throughout the manuscript, including Section 2.8 and Figure legends.

4. Methodological Details and References:

In Section 2.9, provide appropriate references for all computational tools, software, and force fields used. For example, the sentence “The OPLS3e force field was used to describe atomic interactions and replicate how the vaccine models would behave in a biological setting” should be supported with the relevant citation for OPLS3e.

**Answer:**

We have added the required citations in Section 2.9. Specifically, we have included the reference for the OPLS3e force field (Harder et al., 2016) and relevant software documentation for Schrödinger's Desmond.

5. Technical Accuracy in Descriptions:

Revise the sentence “..each vaccine model was solvated in an orthorhombic periodic border box 244 that was filled with TIP3P water molecules” to “..each vaccine model was solvated in an orthorhombic periodic boundary box filled with TIP3P water molecules,” correcting terminology and removing extraneous numbers or formatting errors.

**Answer:** This has been corrected.

6. Figure Quality and Presentation:

Several figures (e.g., Figures 8 and 9) suffer from low resolution, which compromises clarity. These should be replaced with high-resolution versions to enhance visual quality.
For Figure 9, provide separate plots for the vaccine and TLR4 elements, and ensure that axis labels, scales, and legends are clearly visible and interpretable by readers.

**Answer:** This has been corrected.

7. Figure Interpretation:

The manuscript should include a detailed explanation of the data shown in Figure 10, specifically clarifying what biological insights or conclusions can be drawn from the plotted results.

**Answer:** We have expanded the interpretation of Figure 10 in the Results section. We now explicitly discuss the Secondary Structure Element (SSE) stability, explaining how the persistence of alpha-helices and beta-strands (represented by steady horizontal bands) confirms that the vaccine candidates maintain their local folds and do not denature under simulated physiological conditions

8. Major drawback is novelty of vaccine. It should be clearly stated. In addition, it is totally computational work. So, in discussion, write about limitation of work and future direction.

**Answer:** We have clarified the novelty of the vaccine in the Introduction and Discussion, specifically highlighting the pan-genomic selection of core targets conserved across the 2022 outbreak strains. Furthermore, we have added "Limitations and Future Directions" paragraph to the Discussion, where we acknowledge that this study is based on in silico predictions and that experimental validation through in vitro and in vivo immunological assays is a necessary next step.

11. General Recommendations:

**Immunogenic Implications:** We added a discussion on how the selected epitopes target conserved regions of the virus, potentially offering protection against various MPXV clades.

**Adjuvants/Linkers:** We have clarified the roles of the EAAAK stiff linker (connecting the bL12 adjuvant) and the GPGPG/KK flexible linkers in enhancing epitope presentation and vaccine efficacy. **Abbreviations:** We have ensured all abbreviations (e.g., MPXV, TLR4, RMSD) are defined at first use.

**Reviewer #3:**

1. English Language and Grammar:

**Our Response:** We acknowledge that the initial draft had linguistic shortcomings. To address this, the entire manuscript has been rigorously proofread and edited by a team of experienced researchers with expertise in scientific communication. We have corrected awkward phrasing (e.g., changing "computationally creat" to "computationally develop"), fixed subject-verb agreement issues, and ensured a formal academic tone throughout the text. We believe the clarity and flow of the revised manuscript now meet the high standards of PLOS ONE.

2. Comments Methodology and Result section:

**Our Response:** We have carefully addressed every annotation and comment provided within the manuscript file.

3. Figure Quality:

**Our Response:** We have replaced the figures to improve the quality.

4. Overall Presentation:

**Our Response:** Thank you for the feedback. We have improved the overall presentation of the manuscript.
