# Supplementary material for: Pan-genome and reverse vaccinology for a multi-epitope vaccine against circulating post-2022 Monkeypox virus strains
Source: PLoS One. 2026 Jun 22;21(6):e0351881. doi: 10.1371/journal.pone.0351881 (PMC13286271; doi:10.1371/journal.pone.0351881)
Supplement: Attachment — Submitted filename: Response_to_Reviewers_auresp_2.docx [file pone.0351881.s020.docx]

7^th^ May 2026

To

Emily Chenette

Editor-in-Chief,

PLOS One,

Dear Editor,

Special gratitude to you for allowing us to resubmit a revised version of our manuscript entitled as **“Pan-Genome and Reverse Vaccinology for a Multi-Epitope Vaccine Against Circulating Post-2022 Monkeypox Virus Strains”**, (Manuscript ID: PONE-D-25-33735R1) to the PLOS One as a research article. We also appreciate the time and efforts of you and your reviewers have given for this meaningful feedback, which we believe has greatly helped us to enhance the clarity of our article significantly. We have made changes based on your and the reviewers' suggestions, and the revised sections are highlighted in yellow color throughout the revised manuscript. We hope that our response, which is included below, will deal successfully with each reviewer's specific points. Please let us know if you have any additional questions and comments.

Therefore, we want to express our special gratitude to you for your consideration of this manuscript.

**Sincerely Yours,**

Md. Nazmul Hasan, PhD,

Professor,

Department of Genetic Engineering and Biotechnology,

Jashore University of Science and Technology,

Jashore-7408, Bangladesh.

E-mail: [mn.hasan@just.edu.bd](mailto:mn.hasan@just.edu.bd)

Google Scholar Profile: <https://scholar.google.com/citations?user=SP1f9JEAAAAJ&hl=en>

ResearchGate Profile: <https://www.researchgate.net/profile/Dr-Md-Nazmul-Hasan>

ORCID ID: <https://orcid.org/0000-0002-9122-1129>

**Responses of Reviewer Comments**

**Comments of Reviewer 1:**

Comment 1). The authors have addressed all the comments appropriately and revised the manuscript accordingly. The manuscript can be accepted and recommended for publication.

**>>Response:** We thank the reviewer for their careful evaluation of our revised manuscript and for recommending it for publication. We appreciate the constructive feedback provided during the review process

**Comments of Reviewer 2:**

Comment 1). The authors have successfully addressed my previous comments. However, the figure quality (e.g., resolution, clarity of labels, for ex. Figure 9) remains suboptimal. To strengthen the analysis of MEV-TLR4 interactions, I recommend performing a detailed interface characterization using COCOMAPS 2.0 (https://aocdweb.com/BioTools/cocomaps2, Bioinformatics, 2025, doi:10.1093/bioinformatics/btaf606), which provides atomic-level contact maps, interaction classifications (e.g., H-bonds, salt bridges, π-π stacks), and interactive 3D visualizations of protein complexes. The manuscript can then be accepted after these minor revisions and figure improvements.

**>>Response:** Okay. I have done it.
