# Supplementary material for: Pan-genome and reverse vaccinology for a multi-epitope vaccine against circulating post-2022 Monkeypox virus strains
Source: PLoS One. 2026 Jun 22;21(6):e0351881. doi: 10.1371/journal.pone.0351881 (PMC13286271; doi:10.1371/journal.pone.0351881)
Supplement: Peer review history

# Peer review: Decision Letter 0

**DOI:** 10.1371/journal.pone.0351881.r001

**Authors:** Salman Sadullah Usmani

7 Oct 2025

PONE-D-25-33735

Pan-Genome and Reverse Vaccinology for a Multi-Epitope Vaccine Against Circulating Post-2022 Monkeypox Virus Strains

PLOS ONE

Dear Dr. Hasan (Ph.D),

Thank you for submitting your manuscript to PLOS ONE. After careful consideration, we feel that it has merit but does not fully meet PLOS ONE’s publication criteria as it currently stands. Therefore, we invite you to submit a revised version of the manuscript that addresses the points raised during the review process.

Please submit your revised manuscript by Nov 21 2025 11:59PM. If you will need more time than this to complete your revisions, please reply to this message or contact the journal office at plosone@plos.org. When you're ready to submit your revision, log on to https://www.editorialmanager.com/pone/ and select the 'Submissions Needing Revision' folder to locate your manuscript file.

Please include the following items when submitting your revised manuscript:

- A rebuttal letter that responds to each point raised by the academic editor and reviewer(s). You should upload this letter as a separate file labeled 'Response to Reviewers'.
- A marked-up copy of your manuscript that highlights changes made to the original version. You should upload this as a separate file labeled 'Revised Manuscript with Track Changes'.
- An unmarked version of your revised paper without tracked changes. You should upload this as a separate file labeled 'Manuscript'.

If you would like to make changes to your financial disclosure, please include your updated statement in your cover letter. Guidelines for resubmitting your figure files are available below the reviewer comments at the end of this letter.

If applicable, we recommend that you deposit your laboratory protocols in protocols.io to enhance the reproducibility of your results. Protocols.io assigns your protocol its own identifier (DOI) so that it can be cited independently in the future. For instructions see: https://journals.plos.org/plosone/s/submission-guidelines#loc-laboratory-protocols. Additionally, PLOS ONE offers an option for publishing peer-reviewed Lab Protocol articles, which describe protocols hosted on protocols.io. Read more information on sharing protocols at https://plos.org/protocols?utm_medium=editorial-email&utm_source=authorletters&utm_campaign=protocols.

We look forward to receiving your revised manuscript.

Kind regards,

Salman Sadullah Usmani, Ph.D.

Academic Editor

PLOS ONE

Journal Requirements:

When submitting your revision, we need you to address these additional requirements.

1. Please ensure that your manuscript meets PLOS ONE's style requirements, including those for file naming. The PLOS ONE style templates can be found at

https://journals.plos.org/plosone/s/file?id=wjVg/PLOSOne_formatting_sample_main_body.pdf and https://journals.plos.org/plosone/s/file?id=ba62/PLOSOne_formatting_sample_title_authors_affiliations.pdf

2. Please note that PLOS One has specific guidelines on code sharing for submissions in which author-generated code underpins the findings in the manuscript. In these cases, we expect all author-generated code to be made available without restrictions upon publication of the work. Please review our guidelines at https://journals.plos.org/plosone/s/materials-and-software-sharing#loc-sharing-code and ensure that your code is shared in a way that follows best practice and facilitates reproducibility and reuse.

3. We note that your Data Availability Statement is currently as follows: [All relevant data are within the manuscript and its Supporting Information files.]

Please confirm at this time whether or not your submission contains all raw data required to replicate the results of your study. Authors must share the “minimal data set” for their submission. PLOS defines the minimal data set to consist of the data required to replicate all study findings reported in the article, as well as related metadata and methods (https://journals.plos.org/plosone/s/data-availability#loc-minimal-data-set-definition).

For example, authors should submit the following data:

- The values behind the means, standard deviations and other measures reported;

- The values used to build graphs;

- The points extracted from images for analysis.

Authors do not need to submit their entire data set if only a portion of the data was used in the reported study.

If your submission does not contain these data, please either upload them as Supporting Information files or deposit them to a stable, public repository and provide us with the relevant URLs, DOIs, or accession numbers. For a list of recommended repositories, please see https://journals.plos.org/plosone/s/recommended-repositories.

If there are ethical or legal restrictions on sharing a de-identified data set, please explain them in detail (e.g., data contain potentially sensitive information, data are owned by a third-party organization, etc.) and who has imposed them (e.g., an ethics committee). Please also provide contact information for a data access committee, ethics committee, or other institutional body to which data requests may be sent. If data are owned by a third party, please indicate how others may request data access.

4. Please amend the manuscript submission data (via Edit Submission) to include author Saifullah.

5. Please amend your authorship list in your manuscript file to include author Saifullah Saifullah.

6. Please upload a new copy of Figures 1, 2, 5, 6, 8, and 9 as the details are not clear. Please follow the link for more information:  https://journals.plos.org/plosone/s/figures

If the reviewer comments include a recommendation to cite specific previously published works, please review and evaluate these publications to determine whether they are relevant and should be cited. There is no requirement to cite these works unless the editor has indicated otherwise.

Additional Editor Comments:

The manuscript presents a potentially valuable immunoinformatics-based approach for designing a multi-epitope vaccine against the monkeypox virus. However, the reviewers have raised several critical issues that must be addressed before the manuscript can be considered for publication. These include improvements in novelty justification, methodological rigor, figure quality, computational validation depth, and overall presentation.

In my opinion, the manuscript would greatly benefit from a more thorough discussion of existing computational pipelines (PMID: 29680238), resources, and methodological frameworks that have significantly contributed to the field of epitope prediction and multi-epitope vaccine design. In particular, it would strengthen the Introduction and Discussion sections to reference prior work describing web-based vaccine design platforms (PMID: 27633273, 30356876), peptide prediction algorithms — especially those assessing interleukin-inducing potential (PMID: 33034338, 28211521) — and immunoinformatics or therapeutic peptide databases (PMID: 28759605, 30753476, 29688365, etc.). These studies provide important context for understanding how the current approach builds upon and differs from previous pipelines and will help clarify the methodological novelty of this work. A thorough revision addressing all points below is therefore required.

[Note: HTML markup is below. Please do not edit.]

Reviewers' comments:

Reviewer's Responses to Questions

**Comments to the Author**

1. Is the manuscript technically sound, and do the data support the conclusions?

The manuscript must describe a technically sound piece of scientific research with data that supports the conclusions. Experiments must have been conducted rigorously, with appropriate controls, replication, and sample sizes. The conclusions must be drawn appropriately based on the data presented.

Reviewer #1: Partly

Reviewer #2: Partly

Reviewer #3: Yes

**********

2. Has the statistical analysis been performed appropriately and rigorously?

Reviewer #1: N/A

Reviewer #2: N/A

Reviewer #3: N/A

**********

3. Have the authors made all data underlying the findings in their manuscript fully available?

The PLOS Data policy requires authors to make all data underlying the findings described in their manuscript fully available without restriction, with rare exception (please refer to the Data Availability Statement in the manuscript PDF file). The data should be provided as part of the manuscript or its supporting information, or deposited to a public repository. For example, in addition to summary statistics, the data points behind means, medians and variance measures should be available. If there are restrictions on publicly sharing data—e.g. participant privacy or use of data from a third party—those must be specified.

Reviewer #1: Yes

Reviewer #2: Yes

Reviewer #3: Yes

**********

4. Is the manuscript presented in an intelligible fashion and written in standard English?

PLOS ONE does not copyedit accepted manuscripts, so the language in submitted articles must be clear, correct, and unambiguous. Any typographical or grammatical errors should be corrected at revision, so please note any specific errors here.

Reviewer #1: No

Reviewer #2: No

Reviewer #3: No

**********

5. Review Comments to the Author

Please use the space provided to explain your answers to the questions above. You may also include additional comments for the author, including concerns about dual publication, research ethics, or publication ethics. (Please upload your review as an attachment if it exceeds 20,000 characters)

Reviewer #1: The study entitled “Pan-Genome and Reverse Vaccinology for a Multi-Epitope Vaccine Against Circulating Post-2022 Monkeypox Virus Strains” designed a multi-epitope vaccine against Monkeypox virus was designed using pan-genome and immunoinformatics approaches, showing strong immune response potential and stability in computational analyses targeting key viral proteins.

Therefore, this study can be recommended for publication if the following comments are addressed.

1. When the authors are aiming to construct a vaccine, they should include more number of strains to avoid any biasness. Exactly how many strains were considered in this study?

2. What is the cytoplasmic location of the core protein which are non-homologous to human proteins?

3. Why does the authors considered only TLR-4 for docking with the different vaccine construct?

4. Why particularly OPLS3e force field has been used during MD simulation?

5. 50ns MD simulation is not sufficient to prove the stability of the vaccine construct. The authors are advised to extend the simulation to at least 200ns and do it in triplicates.

6. The authors should also perform the MD simulation of the vaccine-target protein complex to check the stability of the complex.

7. The authors should provide the figures for immune simulation result in main text.

8. What are the next steps for experimental validation, and have any specific animal models or immunoassays been considered or recommended?

9. Authors are advised to rewrite the discussion and make the findings of your research clear to the reader.

10. Authors should improve the figure quality and provide a schematic representation of the vaccine construct.

11. The discussion section is not satisfactory. The authors should use other studies that have been done in this field in this section and compare their results with the results of those studies. It is suggested that you use the following studies in this section:

https://doi.org/10.1016/j.biologicals.2024.101782

https://doi.org/10.1007/s13205-024-04022-6

https://doi.org/10.3390/v14112504

https://doi.org/10.1007/s12026-023-09403-2

https://doi.org/10.1016/j.vacun.2024.04.003

12. The manuscript has many typographical and grammatical errors. Please improve the manuscript by someone who is a native English speaker.

Reviewer #2: 1. Novelty and Literature Context:

Several studies have employed immunoinformatics approaches for developing multi-epitope vaccines against monkeypox virus, such as the recent publication [refer DOI: 10.3390/v14112374]. The authors should clearly articulate the novelty and unique contributions of this work relative to prior research. This includes specifying innovative aspects of epitope selection, vaccine construct design, adjuvant/linker usage, molecular docking strategies, or molecular dynamics parameters used.

2. Language and Grammar:

The manuscript requires thorough proofreading and correction of spelling and grammatical errors. For instance, the sentence “Our goal was to Computationally creat and evaluate…” should be revised to “Our goal was to computationally create and evaluate…”. Ensure consistent use of capitalization and verb forms throughout the text.

3. Consistency in Terminology:

The receptor name should be uniformly presented as either “TLR4” or “TLR-4” throughout the manuscript.

4. Methodological Details and References:

In Section 2.9, provide appropriate references for all computational tools, software, and force fields used. For example, the sentence “The OPLS3e force field was used to describe atomic interactions and replicate how the vaccine models would behave in a biological setting” should be supported with the relevant citation for OPLS3e.

5. Technical Accuracy in Descriptions:

Revise the sentence “..each vaccine model was solvated in an orthorhombic periodic border box 244 that was filled with TIP3P water molecules” to “..each vaccine model was solvated in an orthorhombic periodic boundary box filled with TIP3P water molecules,” correcting terminology and removing extraneous numbers or formatting errors.

6. Figure Quality and Presentation:

Several figures (e.g., Figures 8 and 9) suffer from low resolution, which compromises clarity. These should be replaced with high-resolution versions to enhance visual quality.

For Figure 9, provide separate plots for the vaccine and TLR4 elements, and ensure that axis labels, scales, and legends are clearly visible and interpretable by readers.

7. Quantitative Interaction Analysis:

It is important to calculate and report the interaction energy (binding free energy) between the vaccine construct and TLR4 to quantitatively evaluate complex stability. Please include these results with appropriate discussion.

8. Figure Interpretation:

The manuscript should include a detailed explanation of the data shown in Figure 10, specifically clarifying what biological insights or conclusions can be drawn from the plotted results.

9. Additional Structural Analysis:

To deepen the analysis of vaccine-TLR4 interactions, perform co-variation and contact map analysis for the first and last snapshots of the molecular dynamics simulations using the tool available at https://aocdweb.com/BioTools/cocomaps2/. Present these analyses as Supporting Information and describe their relevance to binding stability and interaction dynamics.

10. Major drawback is novelty of vaccine. It should be clearly stated. In addition, it is totally computational work. So in discussion, write about limitation of work and future direction.

11. General Recommendations:

- Include a discussion on the potential immunogenic implications of the selected epitopes in the context of current monkeypox viral variants and clades.

- Provide comparative insight on how the adjuvants and linkers chosen improve vaccine efficacy relative to previous designs.

- Ensure all abbreviations are defined at first use and used consistently thereafter.

Reviewer #3: The current work presents relevant and valuable findings and is generally satisfactory in terms of scientific content. However, there are several critical issues that must be addressed before the manuscript can be considered for publication in PLOS ONE:

English Language and Grammar:

The manuscript contains numerous grammatical errors and awkward sentence constructions. The language needs significant improvement to meet the standards of PLOS ONE. It is strongly recommended that the authors seek assistance from a professional English language editor or a native speaker with experience in scientific writing.

methodology and resulrt section.

comments is added in the manuscript in the methodology and result section

Figure Quality:

Some figures in the manuscript are not clearly visible or are of low resolution. Please ensure that all figures are of high quality and adequately labeled to allow readers to interpret them correctly.

Overall Presentation:

In its current form, the manuscript does not meet the formatting and quality standards expected by PLOS ONE. A thorough revision of the text, figures, and overall presentation is necessary to bring the manuscript up to the required level

**********

6. PLOS authors have the option to publish the peer review history of their article (what does this mean?). If published, this will include your full peer review and any attached files.

If you choose “no”, your identity will remain anonymous but your review may still be made public.

**Do you want your identity to be public for this peer review?**    For information about this choice, including consent withdrawal, please see our Privacy Policy.

Reviewer #1: No

Reviewer #2: **Yes:**   Abdul Rajjak Shaikh

Reviewer #3: No

**********

[NOTE: If reviewer comments were submitted as an attachment file, they will be attached to this email and accessible via the submission site. Please log into your account, locate the manuscript record, and check for the action link "View Attachments". If this link does not appear, there are no attachment files.]

While revising your submission, please upload your figure files to the Preflight Analysis and Conversion Engine (PACE) digital diagnostic tool, https://pacev2.apexcovantage.com/. PACE helps ensure that figures meet PLOS requirements. To use PACE, you must first register as a user. Registration is free. Then, login and navigate to the UPLOAD tab, where you will find detailed instructions on how to use the tool. If you encounter any issues or have any questions when using PACE, please email PLOS at figures@plos.org. Please note that Supporting Information files do not need this step.

Attachment Submitted filename: PONE-D-25-33735.pdf

# Author comment: Author response to Decision Letter 1

**DOI:** 10.1371/journal.pone.0351881.r002

20 Feb 2026

Reviewer #1:

Thank you for the opportunity to revise our manuscript. we appreciate the reviewers' thoughtful comments and suggestions, which have helped us improve the quality and clarity of our work. We have addressed each comment in the point-by-point response below and updated the manuscript accordingly.

1. When the authors are aiming to construct a vaccine, they should include more number of strains to avoid any biasness. Exactly how many strains were considered in this study?

Thank you for your feedback. The study included 16 different strains based on different locations retrieved from the NCBI database, which were downloaded unbiasedly.

2. What is the cytoplasmic location of the core protein which are non-homologous to human proteins?

The cytoplasmic location of the core proteins that are non-homologous to human proteins was given in the supplementary file 3.

3. Why does the authors considered only TLR-4 for docking with the different vaccine construct?

Specifically, TLR-4 is chosen because it recognizes lipopolysaccharides and other microbial components, making it a key trigger for innate immunity and an ideal target to predict vaccine-induced immune activation.

4. Why particularly OPLS3e force field has been used during MD simulation?

The OPLS3e force field is used during MD simulation because it provides highly accurate and optimized parameterization for both proteins and ligands, ensuring reliable modelling of biomolecular interactions and conformational stability.

5. 50ns MD simulation is not sufficient to prove the stability of the vaccine construct. The authors are advised to extend the simulation to at least 200ns and do it in triplicates.

We appreciate the reviewer’s insightful comment. The vaccine model and the receptor protein used in our study are large biomolecules composed of multiple interacting subunits, and therefore, extending the molecular dynamics simulation (MDS) to 200 ns would require substantial computational resources. Since the vaccine-receptor complex simulation in this study primarily aims to evaluate the potential immune response and overall stability of the construct, a 50 ns simulation was initially considered adequate.

6. The authors should also perform the MD simulation of the vaccine-target protein complex to check the stability of the complex.

Thank you for the suggestion; we appreciate the reviewer’s comment and would like to note that the MD simulation of the vaccine-target protein complex has already been performed to evaluate and confirm the stability of the complex

7. The authors should provide the figures for immune simulation result in main text.

We thank the reviewer for this valuable suggestion. The immune simulation result figures are currently included in the supplementary file; however, we will include them in the main text to improve the clarity and accessibility of the findings.

8. What are the next steps for experimental validation, and have any specific animal models or immunoassays been considered or recommended?

The next steps for experimental validation include obtaining funding to synthesize the vaccine construct, followed by in vitro immunoassays to assess immunogenicity, and subsequently conducting animal trials to evaluate immune response and safety in an appropriate model.

9. Authors are advised to rewrite the discussion and make the findings of your research clear to the reader.

We appreciate the reviewer’s suggestion and will revise the discussion to clearly highlight and interpret the key findings of our research for the reader.

10. Authors should improve the figure quality and provide a schematic representation of the vaccine construct.

We thank the reviewer for the suggestion and will improve the figure quality in our revised manuscript.

11. The discussion section is not satisfactory. The authors should use other studies that have been done in this field in this section and compare their results with the results of those studies. It is suggested that you use the following studies in this section:

https://doi.org/10.1016/j.biologicals.2024.101782

https://doi.org/10.1007/s13205-024-04022-6

https://doi.org/10.3390/v14112504

https://doi.org/10.1007/s12026-023-09403-2

https://doi.org/10.1016/j.vacun.2024.04.003

We appreciate the reviewer’s suggestion to incorporate relevant studies into our discussion. We have reviewed the following articles and will cite those findings into our revised discussion.

12. The manuscript has many typographical and grammatical errors. Please improve the manuscript by someone who is a native English speaker.

We thank the reviewer for this comment and will have the manuscript carefully proofread to correct all typographical and grammatical errors.

Reviewer #2:

1. Novelty and Literature Context:

Several studies have employed immunoinformatics approaches for developing multi-epitope vaccines against monkeypox virus, such as the recent publication [refer DOI: 10.3390/v14112374]. The authors should clearly articulate the novelty and unique contributions of this work relative to prior research. This includes specifying innovative aspects of epitope selection, vaccine construct design, adjuvant/linker usage, molecular docking strategies, or molecular dynamics parameters used.

Answer:

Most existing studies, including Kar et al. (2022), were conducted using either a single reference genome (NC_063383.1) or early outbreak sequences from mid-2022. In contrast, our study utilizes 16 distinct whole genomic sequences sourced from diverse geographical regions, specifically including isolates from the ongoing post-2022 outbreak up to August 2024. This ensures that our vaccine targets are based on the most current evolutionary trajectory of the virus. Unlike standard reverse vaccinology that relies on a single proteome, we employed a dual-tool pan-genome approach using Roary and BPGA to identify 80-170 core genes conserved across 100% of the selected strains. This methodology "removes the constraints and biases" of secondary/curated data and ensures the global homogeneity of our vaccine targets, making the resulting vaccine potentially effective against all circulating MPXV clades.

2. Language and Grammar:

The manuscript requires thorough proofreading and correction of spelling and grammatical errors. For instance, the sentence “Our goal was to Computationally creat and evaluate…” should be revised to “Our goal was to computationally create and evaluate…”. Ensure consistent use of capitalization and verb forms throughout the text.

Answer:

We thank the reviewer for this comment and will have the manuscript carefully proofread to correct all typographical and grammatical errors.

3. Consistency in Terminology:

The receptor name should be uniformly presented as either “TLR4” or “TLR-4” throughout the manuscript.

Answer:

We have standardized the terminology and used “TLR4” consistently throughout the manuscript, including Section 2.8 and Figure legends.

4. Methodological Details and References:

In Section 2.9, provide appropriate references for all computational tools, software, and force fields used. For example, the sentence “The OPLS3e force field was used to describe atomic interactions and replicate how the vaccine models would behave in a biological setting” should be supported with the relevant citation for OPLS3e.

Answer:

We have added the required citations in Section 2.9. Specifically, we have included the reference for the OPLS3e force field (Harder et al., 2016) and relevant software documentation for Schrödinger's Desmond.

5. Technical Accuracy in Descriptions:

Revise the sentence “..each vaccine model was solvated in an orthorhombic periodic border box 244 that was filled with TIP3P water molecules” to “..each vaccine model was solvated in an orthorhombic periodic boundary box filled with TIP3P water molecules,” correcting terminology and removing extraneous numbers or formatting errors.

Answer: This has been corrected.

6. Figure Quality and Presentation:

Several figures (e.g., Figures 8 and 9) suffer from low resolution, which compromises clarity. These should be replaced with high-resolution versions to enhance visual quality.

For Figure 9, provide separate plots for the vaccine and TLR4 elements, and ensure that axis labels, scales, and legends are clearly visible and interpretable by readers.

Answer: This has been corrected.

7. Figure Interpretation:

The manuscript should include a detailed explanation of the data shown in Figure 10, specifically clarifying what biological insights or conclusions can be drawn from the plotted results.

Answer: We have expanded the interpretation of Figure 10 in the Results section. We now explicitly discuss the Secondary Structure Element (SSE) stability, explaining how the persistence of alpha-helices and beta-strands (represented by steady horizontal bands) confirms that the vaccine candidates maintain their local folds and do not denature under simulated physiological conditions

8. Major drawback is novelty of vaccine. It should be clearly stated. In addition, it is totally computational work. So, in discussion, write about limitation of work and future direction.

Answer: We have clarified the novelty of the vaccine in the Introduction and Discussion, specifically highlighting the pan-genomic selection of core targets conserved across the 2022 outbreak strains. Furthermore, we have added "Limitations and Future Directions" paragraph to the Discussion, where we acknowledge that this study is based on in silico predictions and that experimental validation through in vitro and in vivo immunological assays is a necessary next step.

11. General Recommendations:

Immunogenic Implications: We added a discussion on how the selected epitopes target conserved regions of the virus, potentially offering protection against various MPXV clades.

Adjuvants/Linkers: We have clarified the roles of the EAAAK stiff linker (connecting the bL12 adjuvant) and the GPGPG/KK flexible linkers in enhancing epitope presentation and vaccine efficacy. Abbreviations: We have ensured all abbreviations (e.g., MPXV, TLR4, RMSD) are defined at first use.

Reviewer #3:

1. English Language and Grammar:

Our Response: We acknowledge that the initial draft had linguistic shortcomings. To address this, the entire manuscript has been rigorously proofread and edited by a team of experienced researchers with expertise in scientific communication. We have corrected awkward phrasing (e.g., changing "computationally creat" to "computationally develop"), fixed subject-verb agreement issues, and ensured a formal academic tone throughout the text. We believe the clarity and flow of the revised manuscript now meet the high standards of PLOS ONE.

2. Comments Methodology and Result section:

Our Response: We have carefully addressed every annotation and comment provided within the manuscript file.

3. Figure Quality:

Our Response: We have replaced the figures to improve the quality.

4. Overall Presentation:

Our Response: Thank you for the feedback. We have improved the overall presentation of the manuscript.

Reviewer #1:

Thank you for the opportunity to revise our manuscript. we appreciate the reviewers' thoughtful comments and suggestions, which have helped us improve the quality and clarity of our work. We have addressed each comment in the point-by-point response below and updated the manuscript accordingly.

1. When the authors are aiming to construct a vaccine, they should include more number of strains to avoid any biasness. Exactly how many strains were considered in this study?

Thank you for your feedback. The study included 16 different strains based on different locations retrieved from the NCBI database, which were downloaded unbiasedly.

2. What is the cytoplasmic location of the core protein which are non-homologous to human proteins?

The cytoplasmic location of the core proteins that are non-homologous to human proteins was given in the supplementary file 3.

3. Why does the authors considered only TLR-4 for docking with the different vaccine construct?

Specifically, TLR-4 is chosen because it recognizes lipopolysaccharides and other microbial components, making it a key trigger for innate immunity and an ideal target to predict vaccine-induced immune activation.

4. Why particularly OPLS3e force field has been used during MD simulation?

The OPLS3e force field is used during MD simulation because it provides highly accurate and optimized parameterization for both proteins and ligands, ensuring reliable modelling of biomolecular interactions and conformational stability.

5. 50ns MD simulation is not sufficient to prove the stability of the vaccine construct. The authors are advised to extend the simulation to at least 200ns and do it in triplicates.

We appreciate the reviewer’s insightful comment. The vaccine model and the receptor protein used in our study are large biomolecules composed of multiple interacting subunits, and therefore, extending the molecular dynamics simulation (MDS) to 200 ns would require substantial computational resources. Since the vaccine-receptor complex simulation in this study primarily aims to evaluate the potential immune response and overall stability of the construct, a 50 ns simulation was initially considered adequate.

6. The authors should also perform the MD simulation of the vaccine-target protein complex to check the stability of the complex.

Thank you for the suggestion; we appreciate the reviewer’s comment and would like to note that the MD simulation of the vaccine-target protein complex has already been performed to evaluate and confirm the stability of the complex

7. The authors should provide the figures for immune simulation result in main text.

We thank the reviewer for this valuable suggestion. The immune simulation result figures are currently included in the supplementary file; however, we will include them in the main text to improve the clarity and accessibility of the findings.

8. What are the next steps for experimental validation, and have any specific animal models or immunoassays been considered or recommended?

The next steps for experimental validation include obtaining funding to synthesize the vaccine construct, followed by in vitro immunoassays to assess immunogenicity, and subsequently conducting animal trials to evaluate immune response and safety in an appropriate model.

9. Authors are advised to rewrite the discussion and make the findings of your research clear to the reader.

We appreciate the reviewer’s suggestion and will revise the discussion to clearly highlight and interpret the key findings of our research for the reader.

10. Authors should improve the figure quality and provide a schematic representation of the vaccine construct.

We thank the reviewer for the suggestion and will improve the figure quality in our revised manuscript.

11. The discussion section is not satisfactory. The authors should use other studies that have been done in this field in this section and compare their results with the results of those studies. It is suggested that you use the following studies in this section:

https://doi.org/10.1016/j.biologicals.2024.101782

https://doi.org/10.1007/s13205-024-04022-6

https://doi.org/10.3390/v14112504

https://doi.org/10.1007/s12026-023-09403-2

https://doi.org/10.1016/j.vacun.2024.04.003

We appreciate the reviewer’s suggestion to incorporate relevant studies into our discussion. We have reviewed the following articles and will cite those findings into our revised discussion.

12. The manuscript has many typographical and grammatical errors. Please improve the manuscript by someone who is a native English speaker.

We thank the reviewer for this comment and will have the manuscript carefully proofread to correct all typographical and grammatical errors.

Reviewer #2:

1. Novelty and Literature Context:

Several studies have employed immunoinformatics approaches for developing multi-epitope vaccines against monkeypox virus, such as the recent publication [refer DOI: 10.3390/v14112374]. The authors should clearly articulate the novelty and unique contributions of this work relative to prior resear

Attachment Submitted filename: Response to Reviewers.docx

# Peer review: Decision Letter 1

**DOI:** 10.1371/journal.pone.0351881.r003

**Authors:** Salman Sadullah Usmani

5 Apr 2026

PONE-D-25-33735R1

Pan-Genome and Reverse Vaccinology for a Multi-Epitope Vaccine Against Circulating Post-2022 Monkeypox Virus Strains

PLOS One

Dear Dr. Hasan (Ph.D),

Thank you for submitting your manuscript to PLOS ONE. After careful consideration, we feel that it has merit but does not fully meet PLOS ONE’s publication criteria as it currently stands. Therefore, we invite you to submit a revised version of the manuscript that addresses the points raised during the review process.

Please submit your revised manuscript by May 20 2026 11:59PM. If you will need more time than this to complete your revisions, please reply to this message or contact the journal office at plosone@plos.org. When you're ready to submit your revision, log on to https://www.editorialmanager.com/pone/ and select the 'Submissions Needing Revision' folder to locate your manuscript file.

Please include the following items when submitting your revised manuscript:

- A letter that responds to each point raised by the academic editor and reviewer(s). You should upload this letter as a separate file labeled 'Response to Reviewers'.
- A marked-up copy of your manuscript that highlights changes made to the original version. You should upload this as a separate file labeled 'Revised Manuscript with Track Changes'.
- An unmarked version of your revised paper without tracked changes. You should upload this as a separate file labeled 'Manuscript'.

If you would like to make changes to your financial disclosure, please include your updated statement in your cover letter. Guidelines for resubmitting your figure files are available below the reviewer comments at the end of this letter.

If applicable, we recommend that you deposit your laboratory protocols in protocols.io to enhance the reproducibility of your results. Protocols.io assigns your protocol its own identifier (DOI) so that it can be cited independently in the future. For instructions see: https://journals.plos.org/plosone/s/submission-guidelines#loc-laboratory-protocols. Additionally, PLOS ONE offers an option for publishing peer-reviewed Lab Protocol articles, which describe protocols hosted on protocols.io. Read more information on sharing protocols at https://plos.org/protocols?utm_medium=editorial-email&utm_source=authorletters&utm_campaign=protocols.

As the corresponding author, your ORCID iD is verified in the submission system and will appear in the published article. PLOS supports the use of ORCID, and we encourage all coauthors to register for an ORCID iD and use it as well. Please encourage your coauthors to verify their ORCID iD within the submission system before final acceptance, as unverified ORCID iDs will not appear in the published article. *Only*    the individual author can complete the verification step; PLOS staff *cannot*    verify ORCID iDs on behalf of authors.

We look forward to receiving your revised manuscript.

Kind regards,

Salman Sadullah Usmani, Ph.D.

Academic Editor

PLOS One

Journal Requirements:

If the reviewer comments include a recommendation to cite specific previously published works, please review and evaluate these publications to determine whether they are relevant and should be cited. There is no requirement to cite these works unless the editor has indicated otherwise.

Please review your reference list to ensure that it is complete and correct. If you have cited papers that have been retracted, please include the rationale for doing so in the manuscript text, or remove these references and replace them with relevant current references. Any changes to the reference list should be mentioned in the rebuttal letter that accompanies your revised manuscript. If you need to cite a retracted article, indicate the article’s retracted status in the References list and also include a citation and full reference for the retraction notice.

[Note: HTML markup is below. Please do not edit.]

Reviewers' comments:

Reviewer's Responses to Questions

**Comments to the Author**

1. If the authors have adequately addressed your comments raised in a previous round of review and you feel that this manuscript is now acceptable for publication, you may indicate that here to bypass the “Comments to the Author” section, enter your conflict of interest statement in the “Confidential to Editor” section, and submit your "Accept" recommendation.

Reviewer #1: All comments have been addressed

Reviewer #2: All comments have been addressed

**********

2. Is the manuscript technically sound, and do the data support the conclusions?

The manuscript must describe a technically sound piece of scientific research with data that supports the conclusions. Experiments must have been conducted rigorously, with appropriate controls, replication, and sample sizes. The conclusions must be drawn appropriately based on the data presented.

Reviewer #1: Yes

Reviewer #2: Yes

**********

3. Has the statistical analysis been performed appropriately and rigorously?

Reviewer #1: N/A

Reviewer #2: N/A

**********

4. Have the authors made all data underlying the findings in their manuscript fully available?

The PLOS Data policy requires authors to make all data underlying the findings described in their manuscript fully available without restriction, with rare exception (please refer to the Data Availability Statement in the manuscript PDF file). The data should be provided as part of the manuscript or its supporting information, or deposited to a public repository. For example, in addition to summary statistics, the data points behind means, medians and variance measures should be available. If there are restrictions on publicly sharing data—e.g. participant privacy or use of data from a third party—those must be specified.

Reviewer #1: Yes

Reviewer #2: Yes

**********

5. Is the manuscript presented in an intelligible fashion and written in standard English?

PLOS ONE does not copyedit accepted manuscripts, so the language in submitted articles must be clear, correct, and unambiguous. Any typographical or grammatical errors should be corrected at revision, so please note any specific errors here.

Reviewer #1: Yes

Reviewer #2: Yes

**********

6. Review Comments to the Author

Please use the space provided to explain your answers to the questions above. You may also include additional comments for the author, including concerns about dual publication, research ethics, or publication ethics. (Please upload your review as an attachment if it exceeds 20,000 characters)

Reviewer #1: The authors have addressed all the comments appropriately and revised the manuscript accordingly. The manuscript can be accepted and recommended for publication.

Reviewer #2: The authors have successfully addressed my previous comments. However, the figure quality (e.g., resolution, clarity of labels, for ex. Figure 9) remains suboptimal. To strengthen the analysis of MEV-TLR4 interactions, I recommend performing a detailed interface characterization using COCOMAPS 2.0 (https://aocdweb.com/BioTools/cocomaps2, Bioinformatics, 2025, doi:10.1093/bioinformatics/btaf606), which provides atomic-level contact maps, interaction classifications (e.g., H-bonds, salt bridges, π-π stacks), and interactive 3D visualizations of protein complexes. The manuscript can then be accepted after these minor revisions and figure improvements.

**********

7. PLOS authors have the option to publish the peer review history of their article (what does this mean?). If published, this will include your full peer review and any attached files.

If you choose “no”, your identity will remain anonymous but your review may still be made public.

**Do you want your identity to be public for this peer review?**    For information about this choice, including consent withdrawal, please see our Privacy Policy.

Reviewer #1: No

Reviewer #2: No

**********

[NOTE: If reviewer comments were submitted as an attachment file, they will be attached to this email and accessible via the submission site. Please log into your account, locate the manuscript record, and check for the action link "View Attachments". If this link does not appear, there are no attachment files.]

To ensure your figures meet our technical requirements, please review our figure guidelines: https://journals.plos.org/plosone/s/figures

You may also use PLOS’s free figure tool, NAAS, to help you prepare publication quality figures: https://journals.plos.org/plosone/s/figures#loc-tools-for-figure-preparation.

NAAS will assess whether your figures meet our technical requirements by comparing each figure against our figure specifications.

# Author comment: Author response to Decision Letter 2

**DOI:** 10.1371/journal.pone.0351881.r004

8 May 2026

Special gratitude to you for allowing us to resubmit a revised version of our manuscript entitled as “Pan-Genome and Reverse Vaccinology for a Multi-Epitope Vaccine Against Circulating Post-2022 Monkeypox Virus Strains”, (Manuscript ID: PONE-D-25-33735R1) to the PLOS One as a research article. We also appreciate the time and effort you and your reviewers have given for this meaningful feedback, which we believe has greatly helped us to enhance the clarity of our article significantly. We have made changes based on your and the reviewers' suggestions, and the revised sections are highlighted in yellow color throughout the revised manuscript. We hope that our response, which is included below, will deal successfully with each reviewer's specific points. Please let us know if you have any additional questions and comments.

Therefore, we want to express our special gratitude to you for your consideration of this manuscript.

Sincerely Yours,

Md. Nazmul Hasan, PhD,

Professor,

Department of Genetic Engineering and Biotechnology,

Jashore University of Science and Technology,

Jashore-7408, Bangladesh.

E-mail: mn.hasan@just.edu.bd

Google Scholar Profile: https://scholar.google.com/citations?user=SP1f9JEAAAAJ&hl=en

ResearchGate Profile: https://www.researchgate.net/profile/Dr-Md-Nazmul-Hasan

Responses of Reviewer Comments

Comments of Reviewer 1:

Comment 1). The authors have addressed all the comments appropriately and revised the manuscript accordingly. The manuscript can be accepted and recommended for publication.

>>Response: We thank the reviewer for their careful evaluation of our revised manuscript and for recommending it for publication. We appreciate the constructive feedback provided during the review process

Comments of Reviewer 2:

Comment 1). The authors have successfully addressed my previous comments. However, the figure quality (e.g., resolution, clarity of labels, for ex. Figure 9) remains suboptimal. To strengthen the analysis of MEV-TLR4 interactions, I recommend performing a detailed interface characterization using COCOMAPS 2.0 (https://aocdweb.com/BioTools/cocomaps2, Bioinformatics, 2025, doi:10.1093/bioinformatics/btaf606), which provides atomic-level contact maps, interaction classifications (e.g., H-bonds, salt bridges, π-π stacks), and interactive 3D visualizations of protein complexes. The manuscript can then be accepted after these minor revisions and figure improvements.

>>Response: Okay. I have done it.

Attachment Submitted filename: Response_to_Reviewers_auresp_2.docx

# Peer review: Decision Letter 2

**DOI:** 10.1371/journal.pone.0351881.r005

**Authors:** Salman Sadullah Usmani

2 Jun 2026

Pan-Genome and Reverse Vaccinology for a Multi-Epitope Vaccine Against Circulating Post-2022 Monkeypox Virus Strains

PONE-D-25-33735R2

Dear Dr. Hasan (Ph.D),

We’re pleased to inform you that your manuscript has been judged scientifically suitable for publication and will be formally accepted for publication once it meets all outstanding technical requirements.

Within one week, you’ll receive an e-mail detailing the required amendments. When these have been addressed, you’ll receive a formal acceptance letter and your manuscript will be scheduled for publication.

An invoice will be generated when your article is formally accepted. Please note, if your institution has a publishing partnership with PLOS and your article meets the relevant criteria, all or part of your publication costs will be covered. Please make sure your user information is up-to-date by logging into Editorial Manager at Editorial Manager® and clicking the ‘Update My Information' link at the top of the page. For questions related to billing, please contact billing support.

If your institution or institutions have a press office, please notify them about your upcoming paper to help maximize its impact. If they’ll be preparing press materials, please inform our press team as soon as possible -- no later than 48 hours after receiving the formal acceptance. Your manuscript will remain under strict press embargo until 2 pm Eastern Time on the date of publication. For more information, please contact onepress@plos.org.

Kind regards,

Salman Sadullah Usmani, Ph.D.

Academic Editor

PLOS One

Additional Editor Comments (optional):

I congratualte the authors for this piece of work.

Reviewers' comments:

Reviewer's Responses to Questions

**Comments to the Author**

1. If the authors have adequately addressed your comments raised in a previous round of review and you feel that this manuscript is now acceptable for publication, you may indicate that here to bypass the “Comments to the Author” section, enter your conflict of interest statement in the “Confidential to Editor” section, and submit your "Accept" recommendation.

Reviewer #2: All comments have been addressed

**********

2. Is the manuscript technically sound, and do the data support the conclusions?

The manuscript must describe a technically sound piece of scientific research with data that supports the conclusions. Experiments must have been conducted rigorously, with appropriate controls, replication, and sample sizes. The conclusions must be drawn appropriately based on the data presented.

Reviewer #2: Yes

**********

3. Has the statistical analysis been performed appropriately and rigorously?

Reviewer #2: N/A

**********

4. Have the authors made all data underlying the findings in their manuscript fully available?

The PLOS Data policy requires authors to make all data underlying the findings described in their manuscript fully available without restriction, with rare exception (please refer to the Data Availability Statement in the manuscript PDF file). The data should be provided as part of the manuscript or its supporting information, or deposited to a public repository. For example, in addition to summary statistics, the data points behind means, medians and variance measures should be available. If there are restrictions on publicly sharing data—e.g. participant privacy or use of data from a third party—those must be specified.

Reviewer #2: No

**********

5. Is the manuscript presented in an intelligible fashion and written in standard English?

PLOS ONE does not copyedit accepted manuscripts, so the language in submitted articles must be clear, correct, and unambiguous. Any typographical or grammatical errors should be corrected at revision, so please note any specific errors here.

Reviewer #2: Yes

**********

6. Review Comments to the Author

Please use the space provided to explain your answers to the questions above. You may also include additional comments for the author, including concerns about dual publication, research ethics, or publication ethics. (Please upload your review as an attachment if it exceeds 20,000 characters)

Reviewer #2: All my comments have been satisfactorily addressed in the revised manuscript. I recommend that it be accepted for publication in its current form.

**********

7. PLOS authors have the option to publish the peer review history of their article (what does this mean?). If published, this will include your full peer review and any attached files.

If you choose “no”, your identity will remain anonymous but your review may still be made public.

**Do you want your identity to be public for this peer review?**    For information about this choice, including consent withdrawal, please see our Privacy Policy.

Reviewer #2: No

**********

# Editor report: Acceptance letter

**DOI:** 10.1371/journal.pone.0351881.r006

**Authors:** Salman Sadullah Usmani

PONE-D-25-33735R2

PLOS One

Dear Dr. Hasan (Ph.D),

I'm pleased to inform you that your manuscript has been deemed suitable for publication in PLOS One. Congratulations! Your manuscript is now being handed over to our production team.

At this stage, our production department will prepare your paper for publication. This includes ensuring the following:

* All references, tables, and figures are properly cited

* All relevant supporting information is included in the manuscript submission,

* There are no issues that prevent the paper from being properly typeset

You will receive further instructions from the production team, including instructions on how to review your proof when it is ready. Please keep in mind that we are working through a large volume of accepted articles, so please give us a few days to review your paper and let you know the next and final steps.

Lastly, if your institution or institutions have a press office, please let them know about your upcoming paper now to help maximize its impact. If they'll be preparing press materials, please inform our press team within the next 48 hours. Your manuscript will remain under strict press embargo until 2 pm Eastern Time on the date of publication. For more information, please contact onepress@plos.org.

You will receive an invoice from PLOS for your publication fee after your manuscript has reached the completed accept phase. If you receive an email requesting payment before acceptance or for any other service, this may be a phishing scheme. Learn how to identify phishing emails and protect your accounts at https://explore.plos.org/phishing.

If we can help with anything else, please email us at customercare@plos.org.

Thank you for submitting your work to PLOS ONE and supporting open access.

Kind regards,

PLOS ONE Editorial Office Staff

on behalf of

Dr. Salman Sadullah Usmani

Academic Editor

PLOS One
